# Global Inequality and Future Burden of Autism Spectrum Disorders: A Frontier and Projection Analysis Based on GBD 2021

**DOI:** 10.1002/brb3.71365

**Published:** 2026-04-06

**Authors:** Jiayu Liu, Aoxi Xu, Zhifeng Zhao, Tao Li, Yuanyuan Dang, Meijing Liu, Yuxin Wang, Hulin Zhao, Yaohua Dai, Jianning Zhang

**Affiliations:** ^1^ Senior Department of Neurosurgery Chinese PLA General Hospital Beijing China; ^2^ Department of General Surgery, Beijing Tsinghua Changgung Hospital, School of Clinical Medicine Tsinghua University Beijing China; ^3^ Department of General Surgery, the First Medical Centre Chinese PLA General Hospital Beijing China; ^4^ Department of Hepatopancreatobiliary Surgery, the First Medical Center Chinese PLA General Hospital Beijing China; ^5^ Capital Center for Children's Health, Capital Medical University Capital Institute of Pediatrics Beijing China; ^6^ Medical School of Chinese PLA Beijing China; ^7^ Department of Integrated Early Childhood Development Capital Institute of Pediatrics Beijing China

**Keywords:** autism spectrum disorder, epidemiology, forecasting, global burden of disease, temporal trend

## Abstract

**Background:**

Autism spectrum disorder (ASD) comprises a group of persistent neurodevelopmental conditions characterized by impairments in communication, restricted behavioral patterns, and social dysfunction. In severe cases, ASD can lead to self‐injury or suicide, imposing a significant burden on individuals, families, and society. This study aimed to comprehensively assess the temporal, demographic, and regional patterns of ASD burden from 1990 to 2021; project future trends; and provide insights into potential causes and public health strategies.

**Methods:**

Data on the incidence, prevalence, and disability‐adjusted life years (DALYs) of ASD were extracted from the Global Burden of Disease (GBD) Study 2021. Temporal trends were evaluated using estimated annual percentage change (EAPC) and Bayesian age‐period‐cohort (BAPC) modeling. We further examined burden distribution by age, sex, region, and sociodemographic index (SDI).

**Results:**

In 2021, an estimated 61.82 million individuals were living with ASD globally, with 1.16 million new cases and 11.54 million DALYs. The global age‐standardized point prevalence, incidence, and DALY rates were 788.3, 18.8, and 147.6 per 100,000 population, respectively—representing increases of 2.0%, 5.2%, and 2.1% since 1990. Regionally, the highest age‐standardized prevalence rate was observed in the High‐income Asia Pacific region, while tropical Latin America had the lowest. At the national level, Japan recorded the highest age‐standardized prevalence, whereas the Republic of Mauritius had the lowest.

**Conclusion:**

ASD poses a rising global public health challenge, with persistent regional disparities and underrecognized burden in adults and females. Current gaps in epidemiological surveillance, etiological understanding, and treatment capacity highlight the urgent need for greater governmental investment in ASD‐related research, early detection strategies, and inclusive care systems.

## Graphical abstract

Using Global Burden of Disease (GBD) 2021 data (1990–2021), we quantified autism spectrum disorder (ASD) prevalence, incidence, and disability‐adjusted life years (DALYs) by age, sex, region, and SDI; assessed temporal trends (EAPC); and projected future burden (BAPC). In 2021, ASD affected 61.82 million people, with marked regional inequality and an underrecognized burden in adults and females, highlighting needs for improved surveillance, early identification, and inclusive care systems.

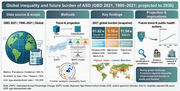

## 1 Introduction

Autism spectrum disorder (ASD) encompasses a group of complex neurodevelopmental conditions characterized by persistent deficits in social communication and interaction, as well as restricted and repetitive behaviors and interests (Ebrahimi Meimand et al. 2023) (Baird et al. 2003). Beyond these core features, individuals with ASD often experience significant challenges throughout their lives, including educational barriers, employment difficulties, and poor integration into society, which can lead to an increased risk of depression, self‐harm, and suicide (Graham et al. 2024; Reid et al. 2024; Hao et al. 2025). The burden of ASD extends beyond the affected individuals to their families and communities, imposing long‐term psychological, social, and financial pressures (Howlin et al. 2004; Howlin et al. 2013).

Despite growing awareness, the global epidemiology of ASD remains poorly characterized. Current estimates suggest that both the prevalence and health burden of ASD are substantial and increasing, especially among children, where the impact is compounded by lifelong care needs and lost developmental opportunities (Marini et al. 2023; Qin et al. 2024). The lifetime economic burden of ASD is estimated to range between $1.4 million and $2.4 million per patient in high‐income countries, underscoring the urgency of effective public health planning (Zhang et al. 2024; Lyall et al. 2017, Buescher et al. 2014).

The Global Burden of Disease (GBD) framework has provided a consistent methodology for estimating disease burden across regions and over time. Previous studies using GBD data, including the GBD 2010, 2019, and early GBD 2021 summaries, have reported stable or slightly increasing trends in ASD prevalence, with relatively limited regional variation and scarce data on adult cases (Baxter et al. 2015, Li et al. 2022). However, these earlier studies often focused solely on descriptive statistics, lacking cross‐country efficiency comparisons, long‐term projections, and multi‐level interpretation of disparities across socio‐demographic settings. Moreover, the COVID‐19 pandemic has introduced additional challenges for individuals with ASD, such as service disruptions, increased psychological stress, and social isolation, which may exacerbate symptoms and alter healthcare‐seeking behaviors (Fernandes and Kwak 2022). Yet, few GBD‐based global are analysed with integrated with probabilistic projection models to examine inequities alongside future trends.

In this study, we address these critical gaps by leveraging the most recent data from the Global Burden of Disease Study 2021 to comprehensively assess the global, regional, and national burden of ASD from 1990 to 2021. Beyond providing updated estimates of prevalence, incidence, and disability‐adjusted life years (DALYs), we introduce three methodological innovations: (1) frontier analysis, to identify countries that underperform or outperform relative to their development level; (2) Bayesian age‐period‐cohort (BAPC) modeling, to forecast future ASD burden trends by sex; and (3) inequality assessment using the Socio‐demographic Index (SDI), to explore U‐shaped relationships and non‐linear trends. Together, these tools provide not only a descriptive portrait of ASD burden but also actionable insights into inequity, health system performance, and future trends, offering a policy‐relevant extension of existing GBD‐based studies.

## 2 Methods

### 2.1 Data Source and Case Definition

The Global Burden of Disease (GBD) 2021 study estimated the burden of 371 diseases and injuries and 88 risk factors from 1990 to 2021 across seven super‐regions, 21 regions, 204 countries and territories (including 21 with subnational locations), and 811 subnational units. Detailed methodologies are available on the GBD website and associated publications (GBD 2021 Diseases and Injuries Collaborators 2024; GBD 2021 Risk Factors Collaborators 2024; GBD 2021 Appendicitis Collaborator Group 2024; GBD 2021 Forecasting Collaborators 2024). Detailed methodologies are available through the IHME GBD Results visualization portal (https://vizhub.healthdata.org/gbd‐results/) and the GBD data‐tools webpage (https://www.healthdata.org/data‐tools‐practices/interactive‐visuals/gbd‐results). (vizhub.healthdata.org)

Our assessment of autism spectrum disorder (ASD) followed the GBD 2021 analytical framework. ASD was defined based on the **International Classification of Diseases, 10th Revision (ICD‐10)**, the **Diagnostic and Statistical Manual of Mental Disorders, Fourth Edition, Text Revision (DSM‐IV‐TR)**, and **DSM‐5** criteria (Santomauro et al. 2024). Standardized GBD methods were used to estimate incidence, prevalence, and disability‐adjusted life years (DALYs) by age, sex, location, and year (GBD 2021 Diseases and Injuries Collaborators 2024; GBD 2021 Risk Factors Collaborators 2024; GBD 2021 Appendicitis Collaborator Group 2024; GBD 2021 Forecasting Collaborators 2024).

### 2.2 Burden Estimation Framework

We used **DisMod‐MR**, a Bayesian meta‐regression tool, to estimate incidence, prevalence, and DALYs of ASD. DALYs were calculated by summing years lived with disability (YLDs) and years of life lost (YLLs) (GBD 2017 Disease and Injury Incidence and Prevalence Collaborators 2018; Xu et al. 2024). Uncertainty was addressed by performing 1,000 model iterations and incorporating errors from multiple sources, including sampling, measurement, and model specification (Xu et al. 2024). Uncertainty intervals (UIs) are presented as the 25th to 975th values of the ordered posterior distribution. A **smoothed spline model** was employed to evaluate the relationship between the burden of ASD and the **Socio‐demographic Index (SDI)** across 21 regions and 204 countries/territories (Xu et al. 2024).

### 2.3 Socio‐demographic Index (SDI) Classification

SDI is a composite measure reflecting development level, incorporating lag‐distributed income per capita, mean educational attainment among individuals aged ≥15 years, and total fertility rate among women <25 years (Rezaei et al. 2023; Lv et al. 2024). Countries and regions were classified into five categories based on SDI values: high, high‐middle, middle, low‐middle, and low SDI (Lv et al. 2024).

### 2.4 Trend Analysis Using EAPC

We calculated **estimated annual percentage changes (EAPCs)** to describe trends in age‐standardized incidence rates (ASIR), prevalence rates (ASPR), and DALYs (ASDR) over time. EAPCs were derived from the log‐linear regression model:

Y=α+βX+ε

where ***Y*** is the natural logarithm of the age‐standardized rate, ***X*** is the calendar year, and ***β*** represents the slope (Zhang et al. 2024). EAPC was calculated as 100 × (exp(*β*) − 1), with 95% confidence intervals (CIs) obtained from the linear model (Ding et al. 2022). If both the EAPC and the lower bound of its 95% CI were >0, the trend was considered increasing; if both were <0, decreasing; otherwise, stable (Ding et al. 2022).

### 2.5 Frontier Performance Analysis

To evaluate **national efficiency in reducing ASD burden** relative to development status, we conducted a **frontier analysis** based on age‐standardized DALYs (ASDD) and SDI values from 1990 to 2021 (Xie et al. 2018; Han et al. 2024). This approach quantified the “effective gap” between observed burden and the optimal frontier, identifying countries with relatively high or low performance in ASD prevention and control at each SDI level.

### 2.6 Future Projection Using BAPC Model

We employed a **Bayesian age‐period‐cohort (BAPC)** model with **integrated nested Laplace approximations** to forecast sex‐specific global ASD burden trends up to the year 2036. This model is known to outperform traditional time‐series or regression approaches in coverage and predictive accuracy (Knoll et al. 2020; Li et al. 2021; Liu et al. 2022; Wu et al. 2024). Forecasted rates include future ASIR, ASPR, and ASDR by sex.

### 2.7 Data Availability and Ethics

All data used in this study were obtained from publicly accessible GBD 2021 repositories via the **Global Health Data Exchange (GHDx)** platform (https://ghdx.healthdata.org/gbd‐results‐tool). No individual‐level or identifiable data were used; thus, ethics approval and informed consent were not required. This study complies with GBD data use policies.

### 2.8 Patient and Public Involvement

No patients or members of the public were involved in the design, conduct, reporting, or dissemination of this research.

### 2.9 Statistical Tools

All statistical analyses and data visualizations were conducted in **R software** (version 4.3.2; R Foundation for Statistical Computing, Vienna, Austria). Packages used included ggplot2 for plotting and BAPC and INLA for Bayesian modeling. *p*‐values < 0.05 were considered statistically significant.

## 3 Results

### 3.1 Global Level

In 2021, 61.82 million prevalent cases of ASD were reported globally, with an age‐standardized point prevalence rate (ASPR) of 788.3 per 100,000 population, representing a 2.0% increase since 1990. That same year, 1.16 million incident cases were recorded, corresponding to an age‐standardized incidence rate (ASIR) of 18.8 per 100,000, an increase of 5.2% since 1990. The total number of disability‐adjusted life years (DALYs) attributed to ASD reached 11.54 million globally, with an age‐standardized DALY rate (ASDR) of 147.6 per 100,000, up by 2.1% compared to 1990 (Table 1).

**TABLE 1:** Prevalence, incidence, and, disability‐adjusted life years (DALYs) of autism spectrum disorders (ASDs) in 2021, and percentage change in age‐standardized rates (ASRs) per 100,000 population by Global Burden of Disease (GBD) region from 1990 to 2021.

		Incidence			Prevalence			DALYs	
Location	Counts 2021 (95% UI)	Age standardized rate 2021 (95% UI)	Percentage change in ASRs from 1990 to 2021	Counts 2021 (95% UI)	Age standardized rate 2021 (95% UI)	Percentage change in ASRs from 1990 to 2021	Counts 2021 (95% UI)	Age standardized rate 2021 (95% UI)	Percentage change in ASRs from 1990 to 2021
Global	1,163,706 (981,645.4 to 1,371,347.3)	18.8 (15.9 to 22.2)	5.2 (3.8 to 6.4)	61,823,539.6 (52,067,672.5 to 72,711,237.7)	788.3 (663.8 to 927.2)	2 (0.4 to 3.1)	11,544,038.1 (7,842,314.7 to 16,288,865.2)	147.6 (100.2 to 208.2)	2.1 (0.6 to 3.4)
Low SDI	354,224.2 (298,241.1 to 417,801.5)	20.5 (17.3 to 24.2)	2.4 (0.6 to 4.5)	9,543,180 (8,011,930.2 to 11,232,296.9)	809.1 (679.5 to 951.2)	2.5 (0.4 to 4.8)	1,795,739.2 (1,229,370.5 to 2,535,526.6)	150.9 (103.4 to 212.1)	3.3 (0.9 to 5.8)
Low‐middle SDI	332,081.2 (279,474.4 to 390,234.6)	17.8 (15 to 20.9)	5.5 (3.5 to 7.9)	14,051,074.9 (11803669.8to16488636.5)	716.9 (601.9 to 841.5)	2.6 (0.4 to 5)	2,633,268.9 (1,788,417.8 to 3,697,132.1)	133.9 (91 to 188)	3.1 (0.7 to 5.4)
Middle SDI	257,391.8 (216,237 to 303,496.5)	16.8 (14.1to19.8)	4.2 (2.2 to 5.9)	17,078 127.9 (14,382,175.5 to 20,164,787.1)	704 (592.9 to 831.2)	5.2 (2.7 to 7.2)	3,196,075.8 (2,163,935.7 to 4,515,251.1)	132.2 (89.5 to 186.7)	5.3 (2.9 to 7.3)
High‐middle SDI	103,433.8 (86,789.8 to 121,573.1)	18.4 (15.4 to21.6)	3.7 (1.1 to 6.2)	10,045,485.9 (8,419,994.9 to 11,895,393.8)	796.5 (668 to 941.4)	2.5 (−0.4 to 5.1)	1,870,636.2 (1,283,602.4 to 2,637,309.1)	150 (102.8 to 211.4)	2.8 (−0.2 to 5.6)
High SDI	115,682.7 (97,297.8 to 136,203.7)	23.4 (19.7 to 27.5)	0.4 (−1.9 to 2.2)	11,056,986 (9,259,241.9 to 13,087,467.7)	1057 (887.2 to 1248.8)	1.8 (−0.6 to 3.6)	2,039,244.2 (1,402,969.9 to 2,848,880.9)	197.9 (136.1 to 276.3)	1.5 (−0.9 to 3.5)
Oceania	3294.5 (2776.8 to 3920.8)	16 (13.5 to 19.1)	−0.8 (−6.6 to 5.8)	96,966.1 (81,551.2 to 116,331.1)	673.2 (566.1 to 807)	−0.8 (−6.2 to 5.7)	18,287.5 (12,475.4to25,630.5)	126.1 (85.7 to 176.8)	−0.5 (−7.2 to 6.4)
Central Europe	10,875 (9142.4 to 12,816)	21.6 (18.2 to 25.5)	0.4 (−3.4 to 3.7)	1,055,437.3 (879,990.7 to 1,249,997.4)	964.4 (810 to 1140.6)	3.2 (−0.7 to 6.8)	194,782.8 (134,724.6 to 272,380)	181 (125.2 to 253)	3.4 (−0.4 to 7.2)
High‐income Asia Pacific	19,606.7 (16,563.7 to 23,002.6)	34.3 (29 to 40.3)	5.1 (1.7 to 8.5)	2,681,977.5 (2,253,494.7 to 3,157,800.9)	1559.5 (1311.3 to 1832.4)	8.1 (4.7 to 11.4)	495,600.7 (343,281.5 to 695,132.4)	293.9 (203.2 to 413)	8.2 (4.7 to 11.2)
Eastern Europe	18,523.1 (15,562.3 to 22035.5)	21.5 (18 to 25.6)	1.9 (−1.3 to 5)	1,8283,89.1 (1,532,202.7 to 2,170,231.7)	928.5 (779.8 to 1102.3)	2.5 (−1 to 5.5)	337,178.8 (231,682.5 to 471,166.8)	173.9 (119.2 to 243.4)	2.6 (−1 to 6.2)
Southeast Asia	85,469.5 (71,881.4 to 101,172.9)	15.8 (13.3 to 18.8)	0.2 (−2.2 to 2.6)	4,784,649.9 (4,024,215.2 to 5,673,821.9)	683 (574.6 to 809.7)	2.9 (0.1 to 5.6)	900,292.7 (612,065.7 to 1,272,739.7)	128.6 (87.5 to 181.7)	3.4 (0.4 to 6.2)
Central Asia	20,660.2 (17,342.5 to 24,272.2)	21 (17.7 to 24.7)	0.8 (−3.3 to 4.6)	858,328.1 (720,725.3 to 1,011,955.9)	886 (744.2 to 1044.4)	1 (−2.7 to 4.8)	161,330.6 (109,690.4 to 228,532.7)	166.4 (113.2 to 235.6)	1.2 (−2.8 to 5.2)
East Asia	81,323.3 (67,880.4 to 96,443.2)	14.7 (12.3 to 17.5)	1.3 (−2.4 to 5)	9,470,370.3 (7,884.683.4 to 11,279,497.2)	660.7 (549.4 to 785.9)	6.9 (2.9 to10.9)	1,774,089.4 (1,197,771.2 to 2,499,467)	125.1 (84.5 to 176.3)	7.1 (3 to 11.1)
Australasia	4,540.5 (3,784.4 to 5,428.4)	26.4 (22 to 31.6)	2.2 (−5.9 to 9.2)	357,327.3 (297,947.5 to 426,205.1)	1191 (993 to 1422.7)	2.5 (−5.9 to 9.4)	66,005.7 (45,662.5 to 93,211.9)	222.6 (154 to 314.3)	2.8 (−5.4 to 10.1)
Southern Latin America	9,010.5 (7563.9 to 10,629.1)	24.2 (20.3 to 28.6)	1.6 (−5.2 to 7.6)	700,574.7 (586,987.3 to 826,295.7)	1056.5 (885.9 to 1245.4)	2.1 (−4.6 to 7.8)	130,654.2 (88,772.8 to 184,968.3)	198.2 (134.7 to 280.3)	2.1 (−5.5 to 8.3)
Western Europe	39,611.4 (33,374.8 to 46,430.1)	20.1 (16.9 to 23.6)	0.8 (−2.2 to 3.8)	3,672,369 (3,082,109.3 to 4,334,849.5)	896.6 (751.6 to 1054.5)	2.3 (−0.9 to 5.2)	678,956.5 (466,623.4 to 944,266.1)	168.3 (115.5 to 234.8)	2.2 (−1.2 to 5.5)
Andean Latin America	9,549.6 (8,001.9 to 11,344.6)	16.1 (13.5 to 19.1)	−0.7 (−5.3 to 4.7)	455,159.8 (380,330.1 to 541,452.5)	684.3 (571.6 to 814.1)	3.1 (−1.5 to 8.8)	85,530.3 (57,762.5 to 120,400.4)	128.5 (86.8 to180.9)	3.4 (−2.5 to 9.6)
Central Latin America	33,120.2 (27.901.7 to 39,001.2)	17.7 (14.9 to 20.8)	−1.3 (−3.8 to 1.4)	1,917,270.6 (1,614,924.3 to 2,268,960.3)	758.6 (639 to897.8)	1.7 (−1 to 4.5)	358,882.5 (242,903.3 to 507,854.8)	142.2 (96.2 to 201.3)	1.9 (−0.9 to 4.9)
Caribbean	6,099.6 (5,120.9 to 7230.2)	16 (13.4 to 19)	−2.1 (−5.5 to 1.8)	320,814.5 (268,946.9 to 382,573.9)	682.5 (572.1 to 813.8)	−0.7 (−3.6 to 2.8)	59,774.1 (40,541.2 to 83,419.7)	127.5 (86.5 to 178.1)	−0.9 (−4.6 to 3.4)
Tropical Latin America	24,144.6 (20,209.9 to 28,596.5)	14.6 (12.2 to 17.3)	0.8 (−2.4 to 3.2)	1,381,082.9 (1,156,543.6 to 1,646,421.9)	614.5 (514.7 to 732.3)	1 (−2.3 to 3.6)	255,967.8 (172,572.6 to 358,107.5)	114.4 (77.1 to 160.3)	1.1 (−2.3 to 4.1)
High‐income North America	47,969.5 (40,550 to 56,638.3)	24.6 (20.8 to 29.1)	1.1 (−1.4 to 3.2)	3,892,297 (3,248,390.9 to 4,605,298.7)	1097.2 (919.2 to 1296.7)	2.3 (−0.4 to 4.5)	713,184.1 (493,087.9 to 997,383.3)	204 (141.1 to 285.5)	1.7 (−1.2 to 4.2)
Central Sub‐Saharan Africa	46,459.6 (39,131 to 55,047.3)	21.7 (18.3 to 25.8)	−0.7 (−5.9 to 6.5)	1,281,660.8 (1,073,126.4 to 1,516,828.4)	885.4 (739.7 to 1046.5)	2.3 (−3.2 to 9.8)	241,038 (164,256.9 to 340,305.3)	164.7 (112.4 to 231.9)	3.1 (−2.5 to 11.1)
North Africa and Middle East	102,934.8 (86,590 to 120,978)	18 (15.1 to 21.1)	−0.8 (−3.7 to 2.2)	4,884,140.3 (4,104,186.4 to 5,765,658.2)	771.8 (648.5 to 910.7)	2.2 (−1.1 to 5.4)	914,908.2 (616,065.8 to 1,286,313.4)	144.2 (97.2 to 202.5)	2.1 (−1.5 to 5.7)
South Asia	252,179.4 (211,753.1 to 296,624.1)	16.7 (14 to 19.6)	5.7 (3.5 to 8.2)	12,848,945 (10,802,916.3 to 15,015,893.1)	686.2 (576.6 to 802)	0.6 (−1.5 to 3.1)	2,399,065.6 (1,637,235.2 to 3,382,018.2)	127.9 (87.3 to 180.3)	1.1 (−1.4 to 3.9)
Eastern Sub‐Saharan Africa	144,955.6 (122,420.6 to 169,985.1)	22.1 (18.7 to 25.9)	−0.1 (−2.7 to 3.2)	4,016,784.6 (3,385,222.5 to 4,713,345.5)	893.5 (752.4 to 1045.2)	2.2 (−0.5 to 5.4)	757,537.3 (516,796 to 1,069,115.5)	166.8 (114.1 to 234.9)	2.8 (−0.1 to 5.8)
Western Sub‐Saharan Africa	186,231.2 (157,399.7 to 218,999.4)	21.9 (18.5 to 25.7)	−0.7 (−2.5 to 1.2)	4,577,312.3 (3,847,537 to 5,399,714.6)	886.2 (745.1 to 1045.6)	0.7 (−1.1 to 2.6)	862,884.2 (590,900.1 to 1,215,653.5)	165.5 (113.5 to 232.4)	1.4 (−0.6 to 3.3)
Southern Sub‐Saharan Africa	17,147.3 (14,437.3 to 20,231.1)	21.9 (18.5 to 25.9)	−0.4 (−3.5 to 2.7)	741,682.7 (623,700.5 to 878038.5)	903.6 (760.1 to 1069.2)	1.5 (−1.5 to 4.6)	138,087.1 (94,596.4 to 194,319.9)	167.7 (114.9 to 236.1)	1 (−2.5 to 4.3)

### 3.2 Regional Level

In 2021, the highest ASPRs were observed in High‐income Asia Pacific (1559.5 per 100,000), Australasia (1191.0), and High‐income North America (1097.2). The lowest ASPRs were reported in Tropical Latin America (614.5), East Asia (660.7), and Oceania (673.2). Similar patterns were seen for ASIR: the highest rates were found in High‐income Asia Pacific (34.3), Australasia (26.4), and High‐income North America (24.6); the lowest were in tropical Latin America (14.6), East Asia (14.7), and Southeast Asia (15.8). The highest ASDRs were reported in High‐income Asia Pacific (293.9), Australasia (222.6), and High‐income North America (204.0), while the lowest were seen in Tropical Latin America (114.4), East Asia (125.1), and Oceania (126.1).

From 1990 to 2021, the largest increases in ASPR were seen in High‐income Asia Pacific (+8.1%), East Asia (+6.9%), and Central Europe (+3.2%). In contrast, the greatest decreases occurred in Oceania (−0.8%) and the Caribbean (−0.7%). ASIR increased most in South Asia (+5.7%), High‐income Asia Pacific (+5.1%), and Australasia (+2.2%), while it decreased most in the Caribbean (−2.1%), Central Latin America (−1.3%), Oceania (−0.8%), and North Africa and the Middle East (−0.8%). ASDR showed the largest increases in High‐income Asia Pacific (+8.2%), East Asia (+7.1%), Central Europe (+3.4%), Southeast Asia (+3.4%), and Andean Latin America (+3.4%), while the greatest decreases occurred in the Caribbean (−0.9%) and Oceania (−0.5%).

The sex‐stratified ASPR, ASIR, and ASDR across 21 GBD regions in 2021 are presented in Supplementary Figures S1–S3. The percentage change in these indicators from 1990 to 2021 by sex is shown in Supplementary Figures S4–S6.

### 3.3 National Level

In 2021, the national ASPR ranged from 588.2 to 1586.9 per 100,000. The highest rates were seen in Japan (1586.9), the Republic of Korea (1506.8), and the Slovak Republic (1487.3); the lowest were in Mauritius (588.2), Angola (614.1), and Zambia (626.1). The ASIR ranged from 14.3 to 34.5 per 100,000, with Japan (34.5), the Republic of Korea (33.9), and Singapore (33.5) reporting the highest and Bangladesh (14.3), Paraguay (14.6), Brazil (14.6), China (14.6), and Fiji (14.8) the lowest. National ASDR ranged from 110.3 to 299.1 per 100,000, highest in Japan (299.1), the Republic of Korea (283.8), and Denmark (280.9); and lowest in Sudan (110.3), Madagascar (114.3), and Dominica (116.5).

The largest increases in ASPR from 1990 to 2021 were observed in Luxembourg (+9.7%), Oman (+9.2%), Egypt (+9.2%), and Japan (+8.8%), while the greatest decreases were noted in Algeria (−1.9%), Paraguay (−1.4%), and Guam (−1.4%). For ASIR, the highest increases occurred in Argentina (+17.1%), American Samoa (+12.6%), and Sudan (+11.0%); the largest declines occurred in Ghana (−19.8%), Greece (−10.7%), and Serbia (−8.5%). Belgium (+10.5%), Italy (+10.4%), and Egypt (+9.0%) had the greatest increases in ASDR, while Mozambique (−1.8%), Nauru (−1.7%), and Guam (−1.6%) had the largest decreases.

**FIGURE 1:**
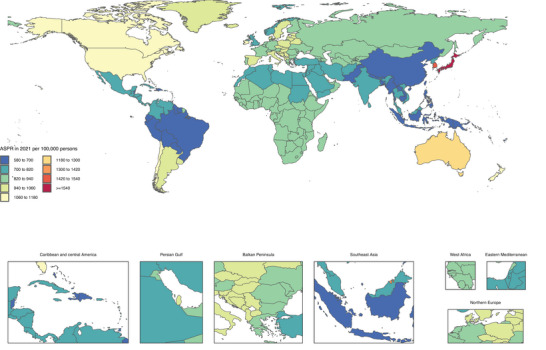
Age‐standardized point prevalence of autism spectrum disorders per 100,000 population in 2023, by country.

The national‐level ASPR for ASD is visualized in Figure 1, while ASIR and ASDR are shown in Supplementary Figures S7 and S8, respectively. Detailed numerical data and changes from 1990 to 2021 are provided in Supplementary Tables S1–S3.

### 3.4 Age and Sex Patterns

**FIGURE 2:**
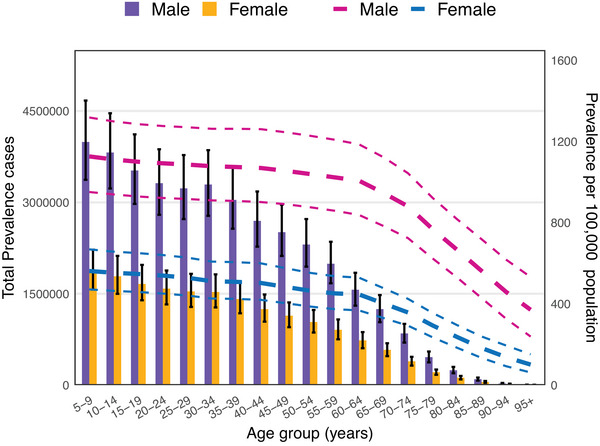
Global number of prevalent ASD cases and age‐ and sex‐specific prevalence rates per 100,000 population in 2023. Lines represent 95% uncertainty intervals for males and females.

**FIGURE 3:**
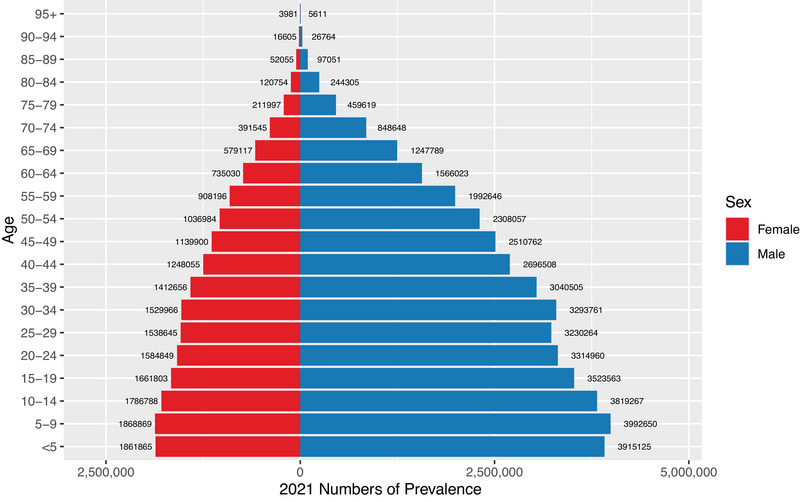
Age‐ and sex‐specific global prevalence rates of autism spectrum disorders per 100,000 population in 2023.

In 2021, the point prevalence of ASD declined with increasing age in both sexes. Males consistently exhibited higher prevalence than females across all age groups. The number of prevalent cases peaked in the 5–9 age group for both sexes. Incidence also peaked before the 5–9 age group, with 784,458 new cases in males and 379,248 in females under 5 years old; data for other age groups were incomplete. Similarly, DALY rates decreased with age and were consistently higher in males. The DALYs peaked in the 5–9 age group for both sexes and declined with advancing age thereafter. The age‐specific and sex‐specific prevalence patterns are shown in Figures 2 and 3. Additional visualizations for prevalence, incidence, and DALYs by age and sex are presented in Supplementary Figures S9–S13.

### 3.5 Association With Socio‐Demographic Index (SDI)

At the regional level, a U‐shaped association was observed between SDI and ASDR. The burden of ASD decreased with increasing SDI up to a value of ∼0.5, then rose as SDI increased further. Regions with higher‐than‐expected ASDRs relative to their SDI included High‐income Asia Pacific, Southern Latin America, Sub‐Saharan Africa (Southern, Central, and Western), and Central Asia. Conversely, regions such as Western Europe, East Asia, Tropical Latin America, Southeast Asia, South Asia, the Caribbean, and Andean Latin America showed lower‐than‐expected burdens.

At the national level, a similar U‐shaped pattern was observed. Countries such as Japan, the Republic of Korea, Singapore, Brunei, Australia, New Zealand, the United States, Canada, Sweden, and Ireland exhibited higher‐than‐expected burdens, while Switzerland, Norway, the United Kingdom, Bangladesh, Haiti, and China had lower‐than‐expected burdens given their SDI.

**FIGURE 4:**
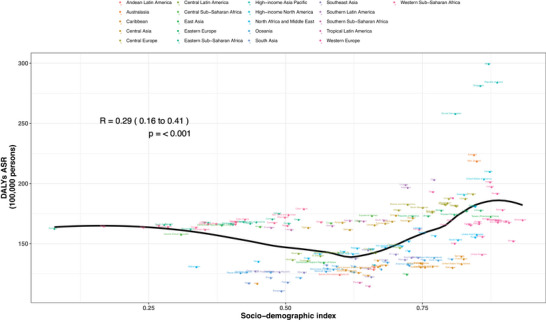
Age‐standardized DALY rates for autism spectrum disorders across 204 countries and territories by Socio‐demographic Index (SDI) in 2023. The solid black line indicates expected DALY values based on SDI and global disease patterns. Each point represents a country's observed age‐standardized DALY rate.

The relationship between ASD burden and SDI at the national level is shown in Figure 4. Supplementary Figures S14 and S15 further illustrate this association across regions, age groups, and SDI strata.

### 3.6 Trends in EAPC

From 1990 to 2021, global EAPCs were 0.07 (95% CI: 0.07–0.08) for ASPR, 0.13 (95% CI: 0.11–0.14) for ASIR, and 0.08 (95% CI: 0.08–0.09) for ASDR, indicating overall upward trends.

Regionally, the highest increase in ASPR occurred in High‐income Asia Pacific (+0.25, 95% CI: 0.23–0.27), while Oceania (−0.03) and the Caribbean (−0.01) showed declines. Middle SDI regions had a moderate rise (+0.17, 95% CI: 0.16–0.18). For ASIR, the Caribbean had the greatest decline (−0.06), while High‐income Asia Pacific again saw the largest increase (+0.17). Similar trends were observed for ASDR, with nearly all regions showing increases except Oceania (−0.02) and the Caribbean (−0.01). EAPC values for ASPR, ASIR, and ASDR across different SDI categories are detailed in Supplementary Table S4.

### 3.7 Frontier Analysis

**FIGURE 5:**
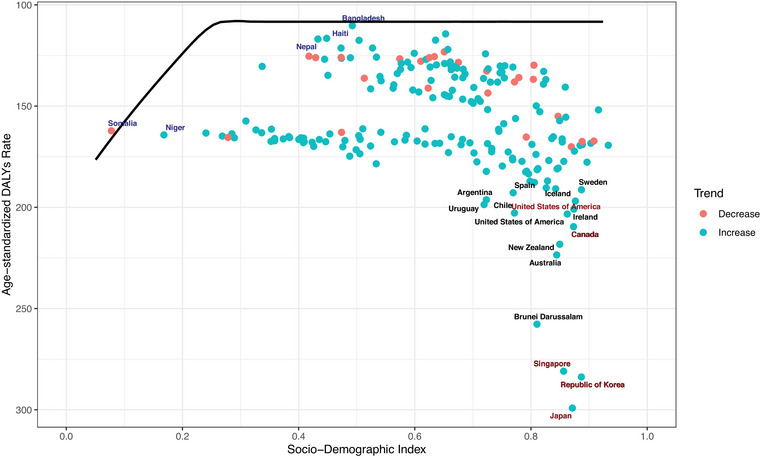
Frontier analysis comparing observed age‐standardized DALY rates for autism spectrum disorders in 2023 against expected values based on SDI. Abbreviations: DALY = disability‐adjusted life year; SDI = Socio‐demographic Index.

Frontier analysis using 2021 DALYs and SDI values revealed that countries with the greatest effective gaps (i.e., underperformers relative to their SDI level) included Japan, the Republic of Korea, Singapore, Brunei, Australia, and New Zealand. Conversely, countries with the smallest effective gaps included Somalia, Bangladesh, Niger, Brazil, Haiti, and Nepal. In this model, the black frontier line represents optimal efficiency, while blue and red dots indicate countries above and below the frontier, respectively. Countries like Bangladesh, Nepal, Haiti, and Somalia were identified as exemplars in ASD prevention and control despite resource limitations. Frontier efficiency analyses are visualized in Figure 5 and Supplementary Figure S16, with quantitative data provided in Supplementary Table S5.

### 3.8 Prediction Analysis

**FIGURE 6:**
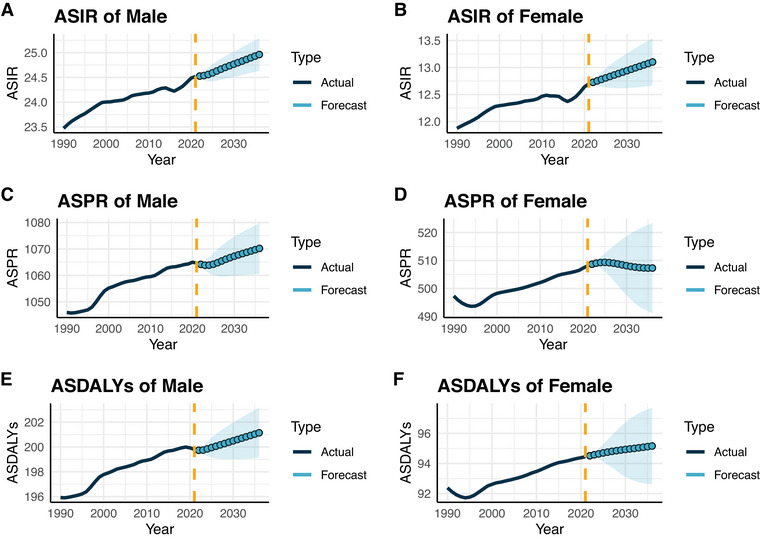
Forecasted trends of age‐standardized incidence (A, B), prevalence (C, D), and DALY rates (E, F) for autism spectrum disorders in males and females, based on Autoregressive Integrated Moving Average (ARIMA) modeling. Data generated from the GBD Results Tool (https://ghdx.healthdata.org/gbd‐results‐tool).

Future projections using the Bayesian age‐period‐cohort (BAPC) model suggest that by 2036, the global prevalence of ASD will rise to 754 per 100,000 among males and 356.6 per 100,000 among females. Meanwhile, incidence rates are projected to decrease to 19.7 per 100,000 for males and 10.7 for females. The DALY rate will remain largely stable for males (199.9 per 100,000) and slightly increase for females (95.7 per 100,000). To validate projections, we also applied the ARIMA model, which yielded consistent results across incidence, prevalence, mortality, and DALYs, supporting the robustness of future trend estimates. Predicted trends for ASD burden using both ARIMA and BAPC models are shown in Figure 6. Supplementary Figures S17–S22 provide detailed projections by sex and SDI for prevalence, incidence, and DALYs.

## 4 Discussion

Our study offers an updated, comprehensive assessment of the global burden of autism spectrum disorders (ASDs) using the latest data from the Global Burden of Disease (GBD) 2021 study. Compared with earlier reports—especially the GBD 2019 and previous studies based on GBD 2010 datasets—our findings capture important new patterns, including the influence of the COVID‐19 pandemic, emerging regional epidemiological shifts, and projections using Bayesian and ARIMA models. These updates provide enhanced value beyond earlier literature in several key areas.

First, our study reaffirms that ASDs represent a considerable and rising global burden, exceeding many other childhood‐onset psychiatric conditions, such as conduct disorder (CD) and attention‐deficit/hyperactivity disorder (ADHD) (Erskine et al. 2014). Notably, while symptoms of ADHD often remit or improve during adolescence (Faraone et al. 2006), ASD symptoms persist into adulthood and are often accompanied by somatic complaints—such as gastrointestinal or respiratory discomfort—that significantly impact long‐term mental health and quality of life (Williams and Gotham 2022). The chronic and pervasive nature of ASD‐related impairments across the life course distinguishes it from other developmental disorders.

Second, our findings provide new insights into sex differences in ASD burden. Males consistently show higher age‐standardized prevalence rates (ASPR) and disability‐adjusted life years (DALYs), which is consistent with the longstanding hypothesis of a female protective effect (Berry et al. 2024). Our study adds to the literature by emphasizing the under‐recognition of ASD in females, potentially due to camouflaging behaviors and differing symptom presentations (Lai and Baron‐Cohen 2015). Additionally, we provided sex‐stratified forecasts of ASD burden using BAPC (with ARIMA for sensitivity), based on GBD 2021 estimates, thereby complementing existing evidence on female under‐recognition and diagnostic camouflaging. Third, our study highlights regional disparities in ASD burden, with the highest age‐standardized incidence, prevalence, and DALY rates observed in high‐income Asia Pacific countries such as Japan, South Korea, and Singapore. We propose that this may be driven more by heightened awareness, active screening efforts, and greater access to healthcare services than by true increases in incidence (Roux et al. 2024). Our results suggest that high‐SDI regions report a higher burden largely due to stronger diagnostic infrastructure, whereas low‐SDI regions may suffer from under‐diagnosis and inadequate care access—despite potentially comparable underlying rates. An important addition in our analysis is the comparison between Japan and Bangladesh. While Japan excels in basic ASD research—often focused on genomics and animal models—Bangladesh has emphasized large‐scale epidemiological and clinical investigations, including socioeconomic and behavioral interventions (Choi et al. 2024; Cho and Talboys 2024) (Fernandes and Kwak 2022; Wong et al. 2024; Bernier et al. 2010; Siddiqi et al. 2023; Faruk et al. 2023; Shahid Khan et al. 2024; Pervin and Hagmayer 2022; Sajib et al. 2022; Blake et al. 2017). This contrast demonstrates that data‐driven public health strategies and real‐world observational studies may have greater near‐term utility for addressing ASD burden at a population level than basic research alone. Beyond the Asia‐Pacific context, high‐income regions such as North America and Western Europe also show comparatively higher reported burden, which may partly reflect stronger diagnostic infrastructure, service availability, and broader case ascertainment across the life course. In contrast, many low‐ and middle‐SDI settings in sub‐Saharan Africa and parts of Latin America may have substantial under‐detection due to limited specialist capacity, constrained access to developmental services, and sociocultural barriers to diagnosis. Regions undergoing rapid health‐system transitions may display heterogeneous patterns, where improvements in awareness and screening can increase detected prevalence without necessarily indicating a true rise in underlying incidence. These regional contrasts underscore the importance of interpreting cross‐national differences as a combined signal of epidemiology and ascertainment.

Fourth, we contextualize the COVID‐19 period and discuss potential indirect pathways (e.g., service disruptions and delayed diagnosis) that may influence observed burden estimates, while acknowledging that our study is not designed to infer causal effects of the pandemic. GBD 2021 reflects that individuals with ASD experienced compounded vulnerabilities, including reduced access to education, mental health services, and social support during lockdowns, as well as delayed diagnoses and treatments due to strained healthcare systems (Fernandes and Kwak 2022). Our study thus offers a timely and unique update on how global crises disproportionately affect neurodevelopmental conditions. Last, through frontier analysis and predictive modeling, we offer novel perspectives on where global ASD burden can be most effectively reduced. Countries like Bangladesh outperform their SDI‐predicted burden due to concentrated efforts in clinical screening, parental education, and targeted interventions. In contrast, some high‐SDI countries, despite advanced research capabilities, underperform due to gaps between basic science and applied care.

The findings of this study have several critical implications for global public health. First, the rising burden and regional inequities in ASD highlight the need for inclusive screening and early intervention programs, particularly in low‐ and middle‐income countries where diagnostic gaps are most profound. Second, the projected increase in female ASD prevalence reinforces the necessity of gender‐sensitive diagnostic criteria and training for healthcare professionals. Third, lessons from countries like Bangladesh underscore the value of scalable, community‐based interventions and policy‐driven public health responses. Last, the pandemic's disproportionate impact on ASD populations emphasizes the need for resilient, neurodiversity‐inclusive health systems capable of maintaining continuity of care during global crises. Taken together, our findings advocate for a globally harmonized, yet locally adaptive, ASD surveillance and intervention framework.

Despite the strengths of our study—including the use of a standardized global database, updated modeling techniques, and a life‐course perspective—several limitations warrant consideration. ASD diagnoses are highly sensitive to cultural, systemic, and temporal factors, leading to substantial heterogeneity across countries. Specifically, temporal changes in diagnostic criteria (e.g., the transition from DSM‐IV‐TR to DSM‐5) and cross‐cultural differences in clinical practice and service pathways may affect case ascertainment and comparability over time and geography. Although the Global Burden of Disease (GBD) framework harmonizes inputs through standardized modeling, residual heterogeneity in diagnostic thresholds and reporting systems may bias the interpretation of long‐term trends, particularly when comparing countries with varying timelines for adopting diagnostic standards. Consequently, the accuracy of national estimates may be compromised by under‐reporting in low‐resource settings or over‐diagnosis in regions with heightened awareness.​ Our analysis relied on secondary data modeled from the GBD 2021 study, which may involve uncertainties in input quality, case definitions, and data completeness. Moreover, while our projections employed robust statistical models such as ARIMA and Bayesian age‐period‐cohort models, these forecasts could still be influenced by unaccounted future shifts in healthcare policy, diagnostic criteria, and intervention access. Furthermore, our study did not examine specific subtypes of ASD or the impact of comorbidities, which could further refine burden estimates.

## 5 Conclusion

This study provides recent and comprehensive global estimates of the burden of autism spectrum disorders based on the GBD 2021 dataset. By integrating updated modeling techniques, cross‐national comparisons, and predictive forecasts, our findings extend beyond earlier research to offer new insights into sex disparities, regional inequalities, and the lasting effects of the COVID‐19 pandemic on individuals with ASD. The contrast between high‐SDI nations with advanced research output but clinical gaps and low‐SDI countries with innovative public health responses highlights the importance of balancing basic science with real‐world, population‐level solutions. As ASD continues to impose a lifelong burden, especially in underserved populations and underrecognized groups such as females and adults, this study underscores the urgency of coordinated global efforts to improve diagnosis, monitoring, and support services across the lifespan. Our results serve as both a call to action and a data‐driven guide for policymakers, clinicians, and researchers working toward equitable and effective ASD care systems worldwide.

## Author Contributions

J. L., Y. D., and J. Z. contributed equally as corresponding authors and jointly conceptualized and designed the study. A. X., J. L., and Z. Z. contributed to data curation, statistical analysis, and data visualization. J. L., A. X., Z. Z., T. L., Y. D., M. L, and Y. W. drafted the initial manuscript. J. L., Y. D., H. Z., and J. Z. served as study guarantors and provided critical revisions for intellectual content. All authors have read and approved the final version of the manuscript. The corresponding authors affirm that all listed authors meet the authorship criteria and that no individuals who meet the criteria have been omitted.

## AI Usage Declaration

AI‐assisted tools were used only for language polishing and grammar checking. All scientific content, analyses, and interpretations were produced by the authors, who take full responsibility for the integrity and accuracy of the work.

## Funding

The authors have nothing to report.

## Conflicts of Interest

The authors declare no conflicts of interest.

## Dissemination to Participants and the Public

Study findings will be disseminated through academic conferences, peer‐reviewed journal publication, and science communication platforms. As no participants were recruited, direct dissemination to individuals involved in the study is not applicable.

## Guarantor Statement

The guarantors of this manuscript (JYL) affirm that this report is an honest, accurate, and transparent account of the study. No important aspects of the study have been omitted, and any discrepancies from the original study plan have been clearly explained.

## Supporting information




**Supplementary Material**: brb371365‐sup‐0001‐SuppMat.pdf

## Data Availability

All data used in this study are publicly available from the Global Burden of Disease (GBD) 2021 database via the Global Health Data Exchange (https://ghdx.healthdata.org/gbd‐results‐tool). Additional processed datasets are available from the corresponding author upon reasonable request.
