# Supplementary material for: Global Inequality and Future Burden of Autism Spectrum Disorders: A Frontier and Projection Analysis Based on GBD 2021
Source: Brain Behav. 2026 Apr 6;16(4):e71365. doi: 10.1002/brb3.71365 (PMC13053662; doi:10.1002/brb3.71365)
Supplement: Supplementary file 1 — Supplementary Material: brb371365‐sup‐0001‐SuppMat.pdf [file BRB3-16-e71365-s001.pdf]

Supplementary table and figure legends

Figure S1: The age-standardized point prevalence of autism spectrum disorders in 2021 for the 21 Global Burden of Disease regions, by sex. (Generated from data available from http://ghdx.healthdata.org/gbd-results-tool).

Figure S2: The age-standardized incidence of autism spectrum disorders in 2021 for the 21 Global Burden of Disease regions, by sex. (Generated from data available from http://ghdx.healthdata.org/gbd-results-tool).

Figure S3: The age-standardized DALYs rates of autism spectrum disorders in 2021 for the 21 Global Burden of Disease regions, by sex. DALYs = disability adjusted life years. (Generated from data available from http://ghdx.healthdata.org/gbd-results-tool).

Figure S4: The percentage change in the age-standardized point prevalence of autism spectrum disorders from 1990 to 2021 for the 21 Global Burden of Disease regions, by sex. (Generated from data available from http://ghdx.healthdata.org/gbd-results-tool).

Figure S5: The percentage change in the age-standardized incidence of autism spectrum disorders from 1990 to 2021 for the 21 Global Burden of Disease regions, by sex. (Generated from data available from http://ghdx.healthdata.org/gbd-results-tool).

Figure S6: The percentage change in the age-standardized DLAYs of autism spectrum disorders from 1990 to 2021 for the 21 Global Burden of Disease regions, by sex. (Generated from data available from http://ghdx.healthdata.org/gbd-results-tool).

Figure S7: Age standardized incidence of autism spectrum disorders per 100 000 population in 2021, by country. (generated from data available at https://ghdx.healthdata.org/gbd-results-tool).

Figure S8: Age standardized DLAYs of autism spectrum disorders per 100 000 population in 2021, by country. DLAYs= disability adjusted life years. (generated from data available at https://ghdx.healthdata.org/gbd-results-tool).

Figure S9: Global prevalence rate of autism spectrum disorders, by age and sex, in 2021. Red and blue shadows indicate 95% upper and lower uncertainty intervals, respectively. (Generated from data available from http://ghdx.healthdata.org/gbd-results-tool).

Figure S10: Global number of incidence of autism spectrum disorders per 100,000 population, by age and sex, in 2021. (Generated from data available from

http://ghdx.healthdata.org/gbd-results-tool).

Figure S11: Global incidence rate of autism spectrum disorders per 100,000 population, by age and sex, in 2021; Red and blue shadows indicate 95% upper and lower uncertainty intervals, respectively. (Generated from data available from http://ghdx.healthdata.org/gbd-results-tool)

Figure S12: Global number of DALYs of autism spectrum disorders per 100,000 population, by age and sex, in 2021. (Generated from data available from http://ghdx.healthdata.org/gbd-results-tool).

Figure S13: Global DALYs rate of autism spectrum disorders per 100,000 population, by age and sex, in 2021; Red and blue shadows indicate 95% upper and lower uncertainty intervals, respectively. (Generated from data available from http://ghdx.healthdata.org/gbd-results-tool

Figure S14: Number of DALYs globally and DALYs rate of autism spectrum disorders per 100 000 population by age and sex in 2021. Dotted and dashed lines indicate 95% upper and lower uncertainty intervals, respectively. (Generated from data available from http://ghdx.healthdata.org/gbd-results-tool).

Figure S15: Disability adjusted life years (DALYs) rates of autism spectrum disorders for the 22 Global Burden of Disease regions by sociodemographic index,1990-2021. Thirty-two points are plotted for each region and show the observed age-standardized DALYs rates from 1990 to 2021 for that region. DLAYs= disability adjusted life years. Expected values, based on sociodemographic index and disease rates in all locations, are shown as a solid line. Regions above the solid line represent a higher than expected burden (e g, High−income Asia Pacific), and regions below the line show a lower than expected burden (e g, East Asia, Tropical Latin America)(generated from data available at https://ghdx.healthdata.org/gdb-results-tool).

Figure S16: Frontier analysis based on SDI and autism spectrum disorders DALYs rate from 1990 to 2021. DLAYs= disability adjusted life years. SDI= sociodemographic index

Figure S17: Projected number of new ASPR cases for autism spectrum disorders in male in 2036 according to the SDI. ASPR= age-standardized prevalence rate; SDI= sociodemographic index.

Figure S18: Projected number of new ASIR cases for autism spectrum disorders in male in 2036 according to the SDI. ASIR=age-standardized incidence rate; SDI= sociodemographic index.

Figure S19: Projected number of new age standardized DALYs cases for autism spectrum disorders in males in 2036 according to the SDI. DLAYs= disability adjusted life years. SDI=sociodemographic index.

Figure S20: Projected number of new ASPR cases for autism spectrum disorders in females in 2036 according to the SDI. ASPR=age-standardized prevalence rate; SDI= sociodemographic index.

Figure S21: Projected number of new ASIR cases for autism spectrum disorders in females in 2036 according to the SDI. ASIR= age-standardized incidence rate; SDI=sociodemographic index.

Figure S22: Projected number of new age standardized DALYs cases for autism spectrum disorders in females in 2036 according to the SDI. DLAYs= disability adjusted life years. SDI= sociodemographic index.

Table S1: Prevalent cases of autism spectrum disorders in 1990 and 2021 and the percentage change in the age-standardized rates (ASRs) per 100,000, by location(Generated from data available from http://ghdx.healthdata.org/gbd-results-tool)

Table S2: Incidence cases of autism spectrum disorders in 1990 and 2021 and the percentage change in the age-standardized rates (ASRs) per 100,000, by location(Generated from data available from http://ghdx.healthdata.org/gbd-results-tool)

Table S3: DLAYs due to autism spectrum disorders in 1990 and 2021 and the percentage change in the age-standardized rates (ASRs) per 100,000, by location (Generated from data available from http://ghdx.healthdata.org/gbd-results-tool)

Table S4: EAPC of ASPR, ASIR, and DLAYs for autism spectrum disorders in countries with five SDI levels from 1990 to 2021. ASPR age-standardized prevalence rate, ASIR age-standardized incidence rate, EAPC estimated annual percentage change, DLAYs= disability adjusted life years. (Generated from data available from http://ghdx.healthdata.org/gbd-results-tool)

Table S5: Frontier DLAYs, and effective difference by country or territory in 2021. DLAYs= disability adjusted life years. (Generated from data available from http://ghdx.healthdata.org/gbd-results-tool)

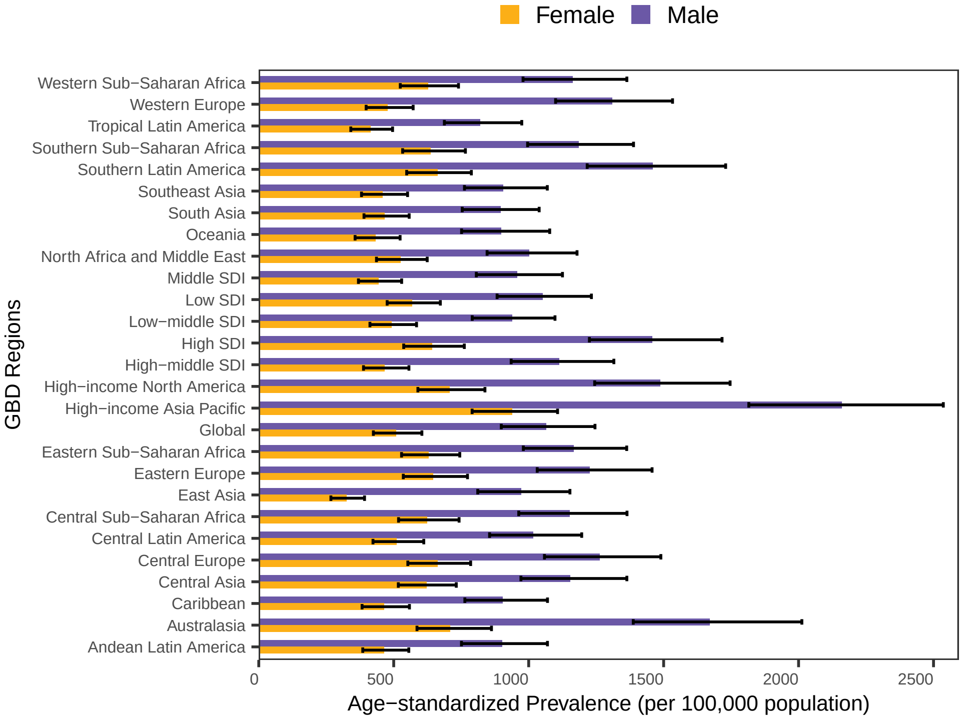
Female
Male
GBD Regions
Western Sub−Saharan Africa
Western Europe
Tropical Latin America
Southern Sub−Saharan Africa
Southern Latin America
Southeast Asia
South Asia
Oceania
North Africa and Middle East
Middle SDI
Low SDI
Low−middle SDI
High SDI
High−middle SDI
High−income North America
High−income Asia Pacific
Global
Eastern Sub−Saharan Africa
Eastern Europe
East Asia
Central Sub−Saharan Africa
Central Latin America
Central Europe
Central Asia
Caribbean
Australasia
Andean Latin America
0
500
1000
1500
2000
2500
Age−standardized Prevalence (per 100,000 population)

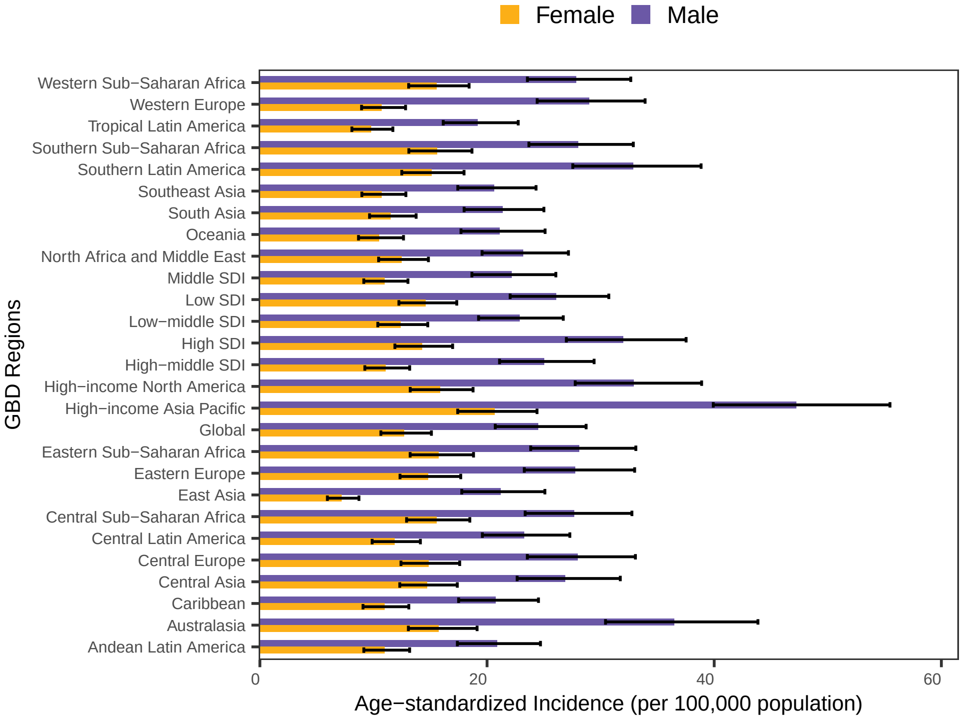

Female
Male
GBD Regions
Western Sub−Saharan Africa
Western Europe
Tropical Latin America
Southern Sub−Saharan Africa
Southern Latin America
Southeast Asia
South Asia
Oceania
North Africa and Middle East
Middle SDI
Low SDI
Low−middle SDI
High SDI
High−middle SDI
High−income North America
High−income Asia Pacific
Global
Eastern Sub−Saharan Africa
Eastern Europe
East Asia
Central Sub−Saharan Africa
Central Latin America
Central Europe
Central Asia
Caribbean
Australasia
Andean Latin America
0
20
40
60
Age−standardized Incidence (per 100,000 population)

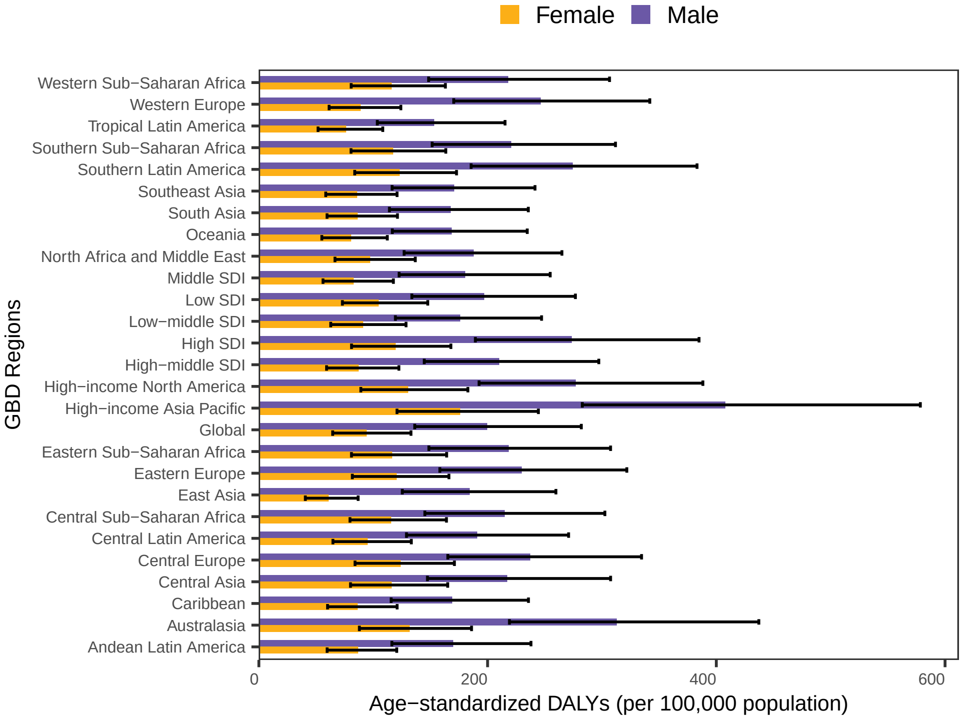
Female
Male
GBD Regions
Western Sub−Saharan Africa
Western Europe
Tropical Latin America
Southern Sub−Saharan Africa
Southern Latin America
Southeast Asia
South Asia
Oceania
North Africa and Middle East
Middle SDI
Low SDI
Low−middle SDI
High SDI
High−middle SDI
High−income North America
High−income Asia Pacific
Global
Eastern Sub−Saharan Africa
Eastern Europe
East Asia
Central Sub−Saharan Africa
Central Latin America
Central Europe
Central Asia
Caribbean
Australasia
Andean Latin America
0
200
400
600
Age−standardized DALYs (per 100,000 population)

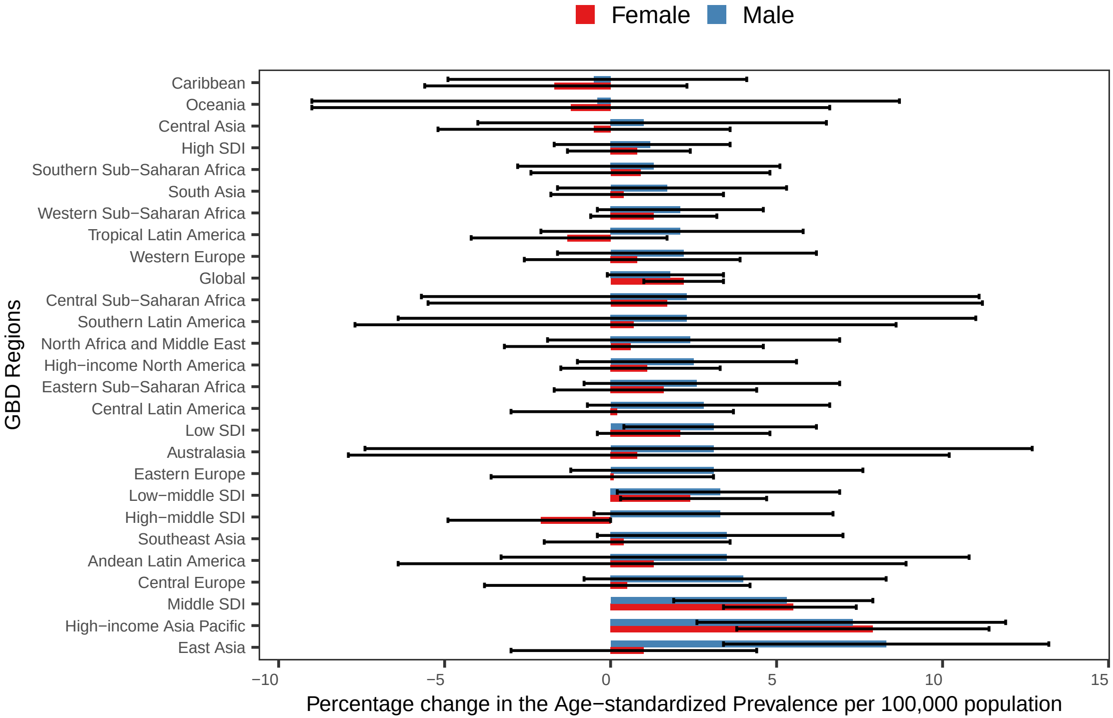

Female
Male
GBD Regions
Caribbean
Oceania
Central Asia
High SDI
Southern Sub−Saharan Africa
South Asia
Western Sub−Saharan Africa
Tropical Latin America
Western Europe
Global
Central Sub−Saharan Africa
Southern Latin America
North Africa and Middle East
High−income North America
Eastern Sub−Saharan Africa
Central Latin America
Low SDI
Australasia
Eastern Europe
Low−middle SDI
High−middle SDI
Southeast Asia
Andean Latin America
Central Europe
Middle SDI
High−income Asia Pacific
East Asia
−10
−5
0
5
10
15
Percentage change in the Age−standardized Prevalence per 100,000 population

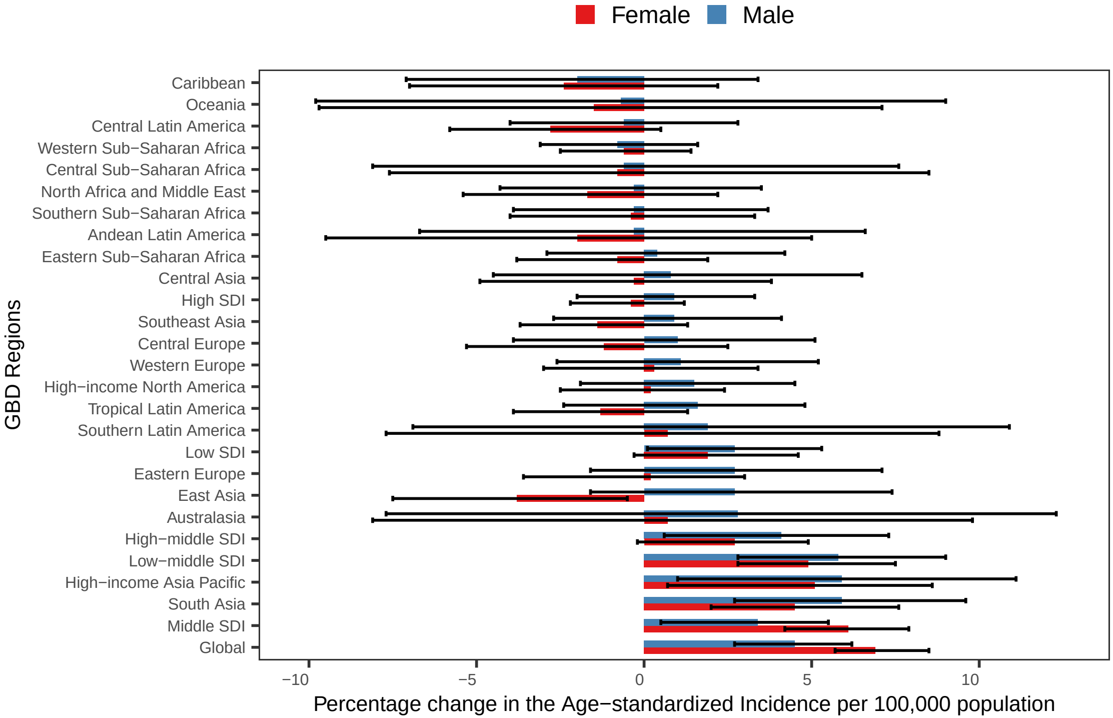

Female
Male
GBD Regions
Caribbean
Oceania
Central Latin America
Western Sub−Saharan Africa
Central Sub−Saharan Africa
North Africa and Middle East
Southern Sub−Saharan Africa
Andean Latin America
Eastern Sub−Saharan Africa
Central Asia
High SDI
Southeast Asia
Central Europe
Western Europe
High−income North America
Tropical Latin America
Southern Latin America
Low SDI
Eastern Europe
East Asia
Australasia
High−middle SDI
Low−middle SDI
High−income Asia Pacific
South Asia
Middle SDI
Global
−10
−5
0
5
10
Percentage change in the Age−standardized Incidence per 100,000 population

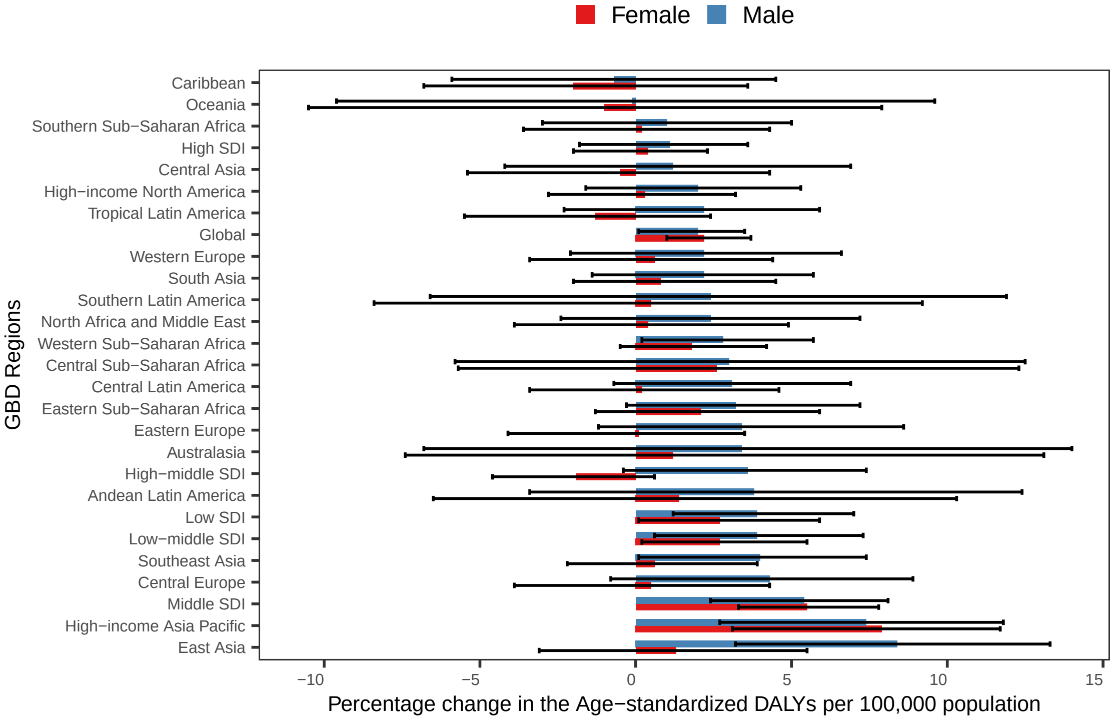

Female
Male
GBD Regions
Caribbean
Oceania
Southern Sub−Saharan Africa
High SDI
Central Asia
High−income North America
Tropical Latin America
Global
Western Europe
South Asia
Southern Latin America
North Africa and Middle East
Western Sub−Saharan Africa
Central Sub−Saharan Africa
Central Latin America
Eastern Sub−Saharan Africa
Eastern Europe
Australasia
High−middle SDI
Andean Latin America
Low SDI
Low−middle SDI
Southeast Asia
Central Europe
Middle SDI
High−income Asia Pacific
East Asia
−10
−5
0
5
10
15
Percentage change in the Age−standardized DALYs per 100,000 population

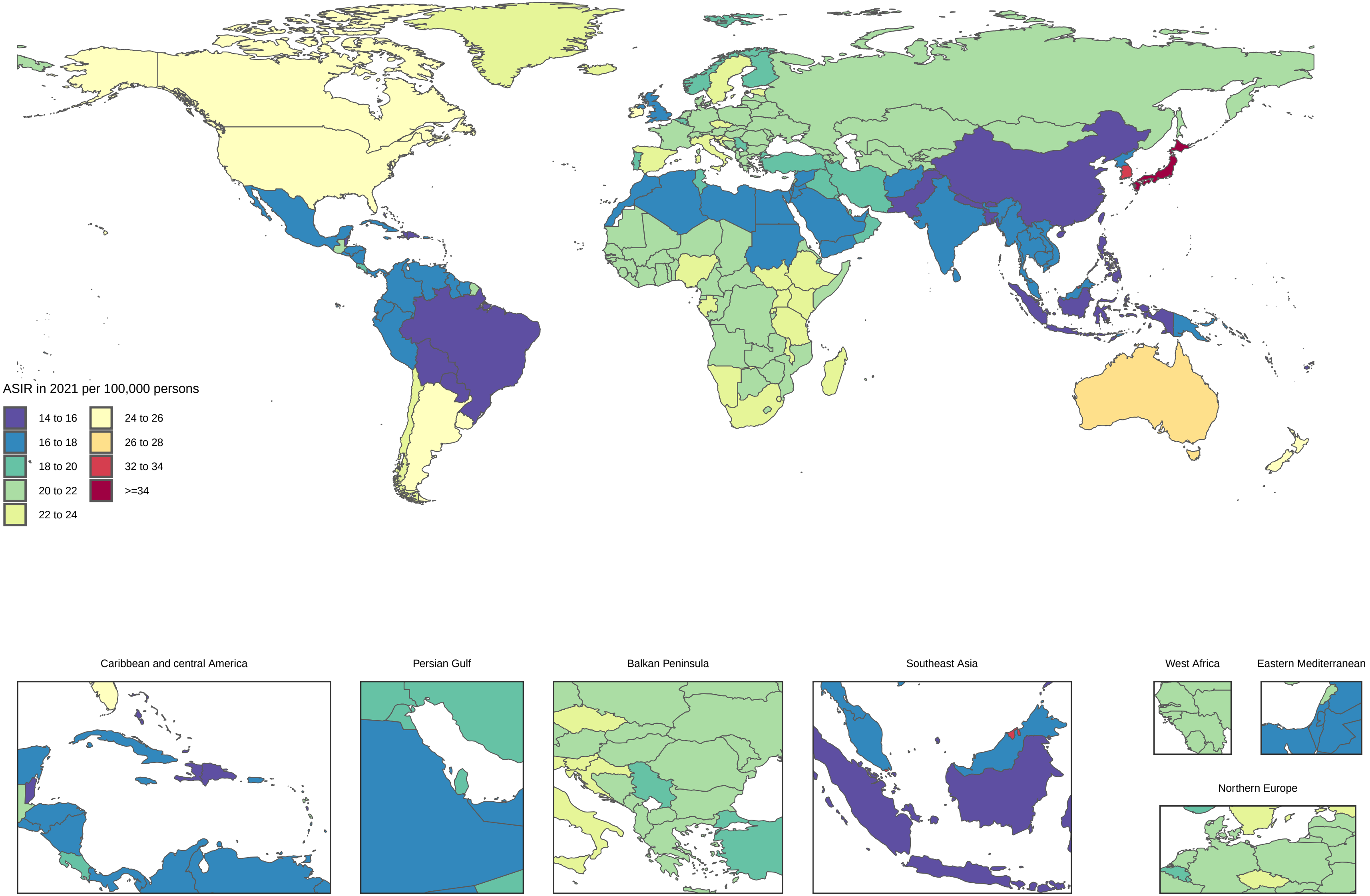

ASIR in 2021 per 100,000 persons
14 to 16
16 to 18
18 to 20
20 to 22
22 to 24
24 to 26
26 to 28
32 to 34
>=34
Caribbean and central America
Persian Gulf
Balkan Peninsula
Southeast Asia
West Africa
Eastern Mediterranean
Northern Europe

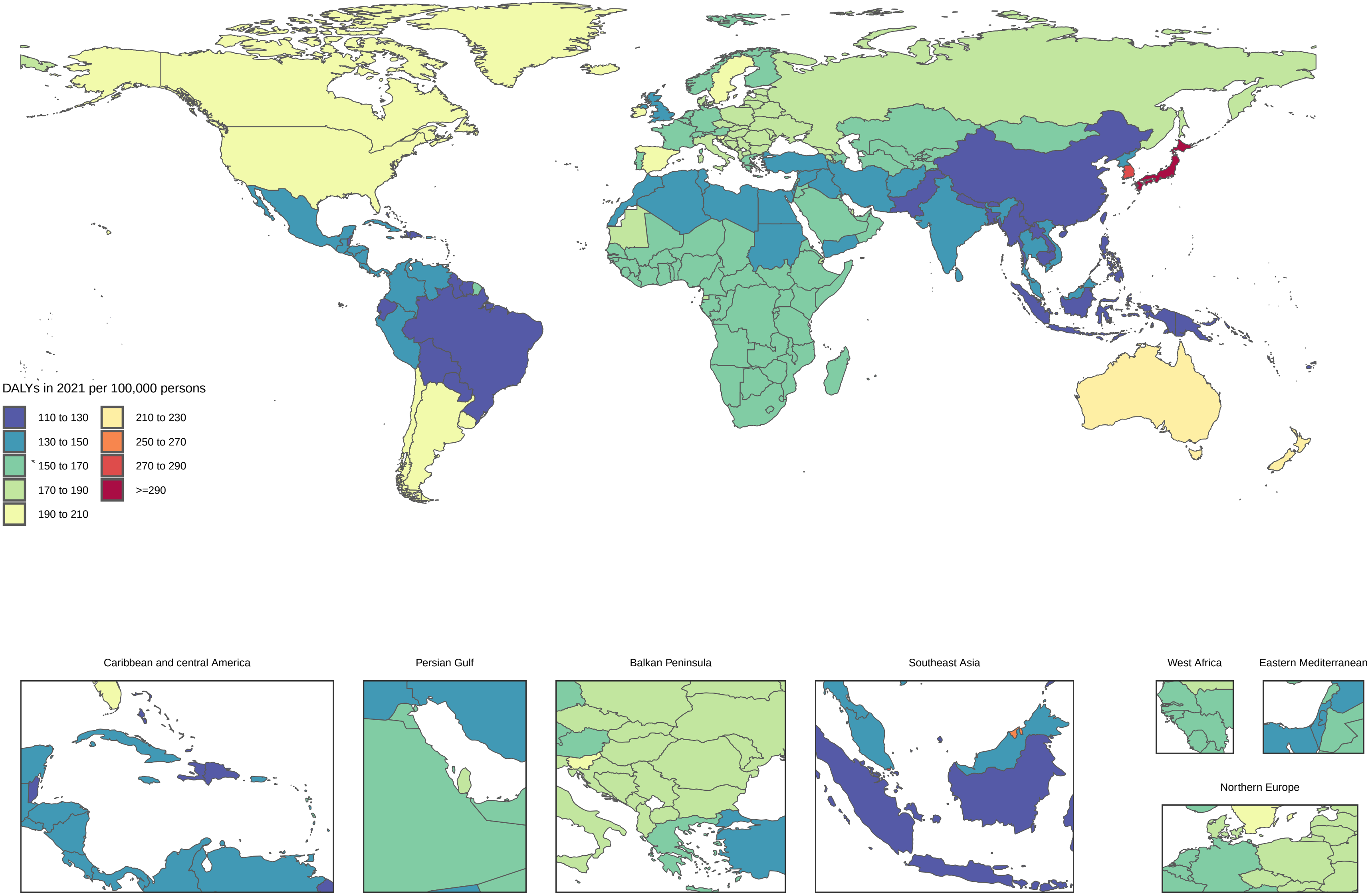
DALYs in 2021 per 100,000 persons
110 to 130
130 to 150
150 to 170
170 to 190
190 to 210
210 to 230
250 to 270
270 to 290
>=290
Caribbean and central America
Persian Gulf
Balkan Peninsula
Southeast Asia
West Africa
Eastern Mediterranean
Northern Europe

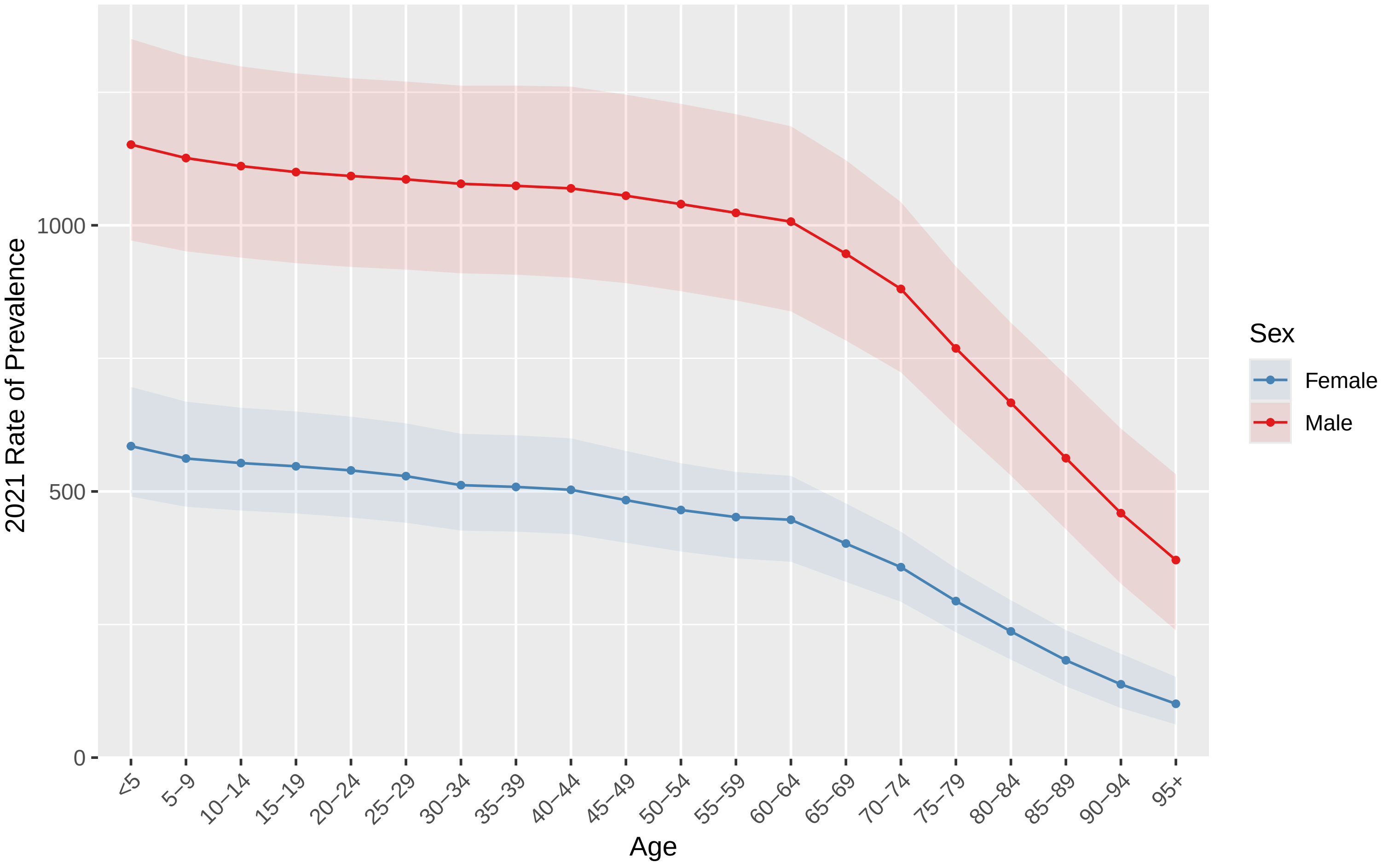

2021 Rate of Prevalence
0
500
1000
Age
<5
5–9
10–14
15–19
20–24
25–29
30–34
35–39
40–44
45–49
50–54
55–59
60–64
65–69
70–74
75–79
80–84
85–89
90–94
95+
Sex
Female
Male

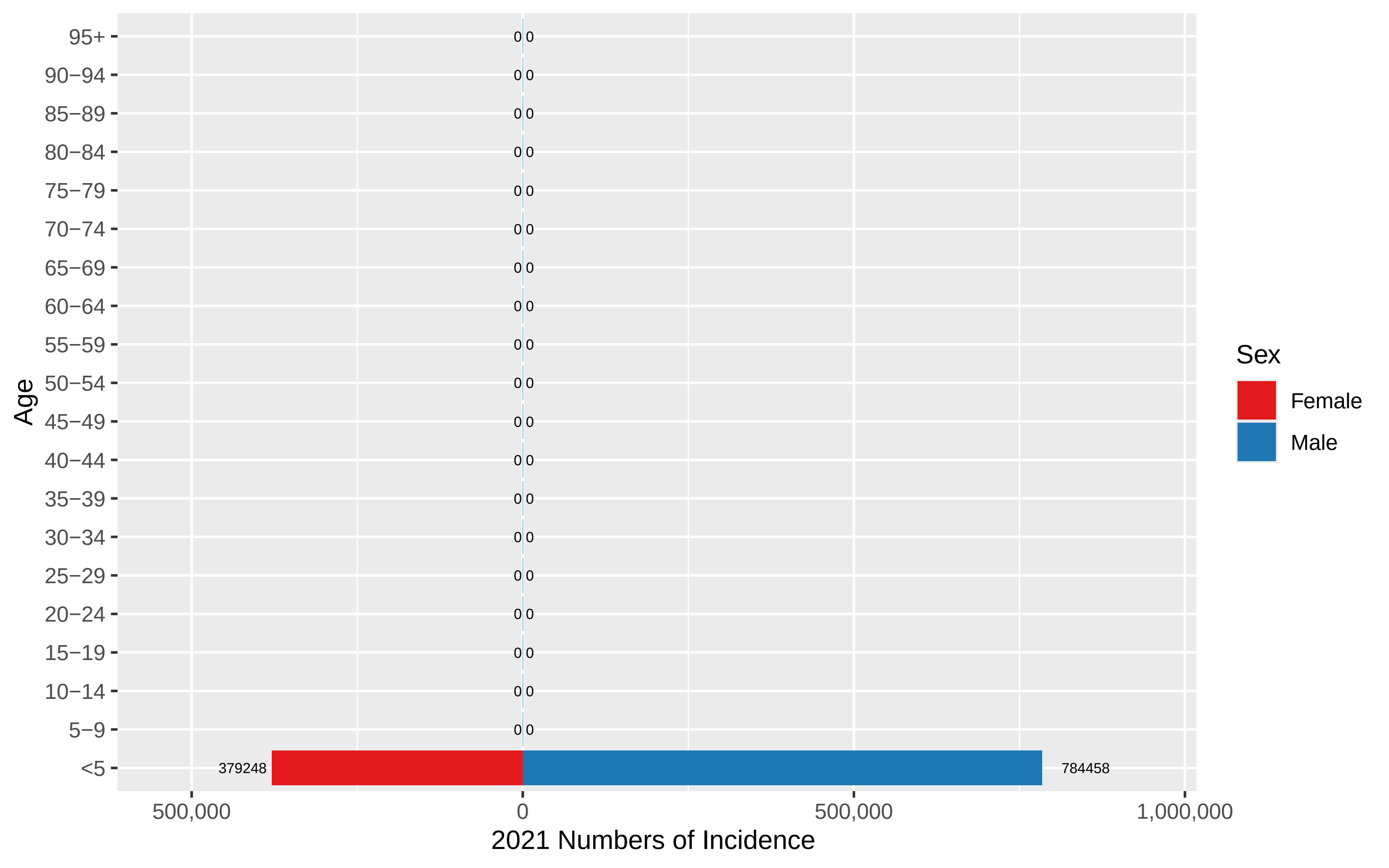

95+
90−94
85−89
80−84
75−79
70−74
65−69
60−64
55−59
50−54
45−49
40−44
35−39
30−34
25−29
20−24
15−19
10−14
5−9
<5
Age
0 0
379248
784458
500,000
0
500,000
1,000,000
2021 Numbers of Incidence
Sex
Female
Male

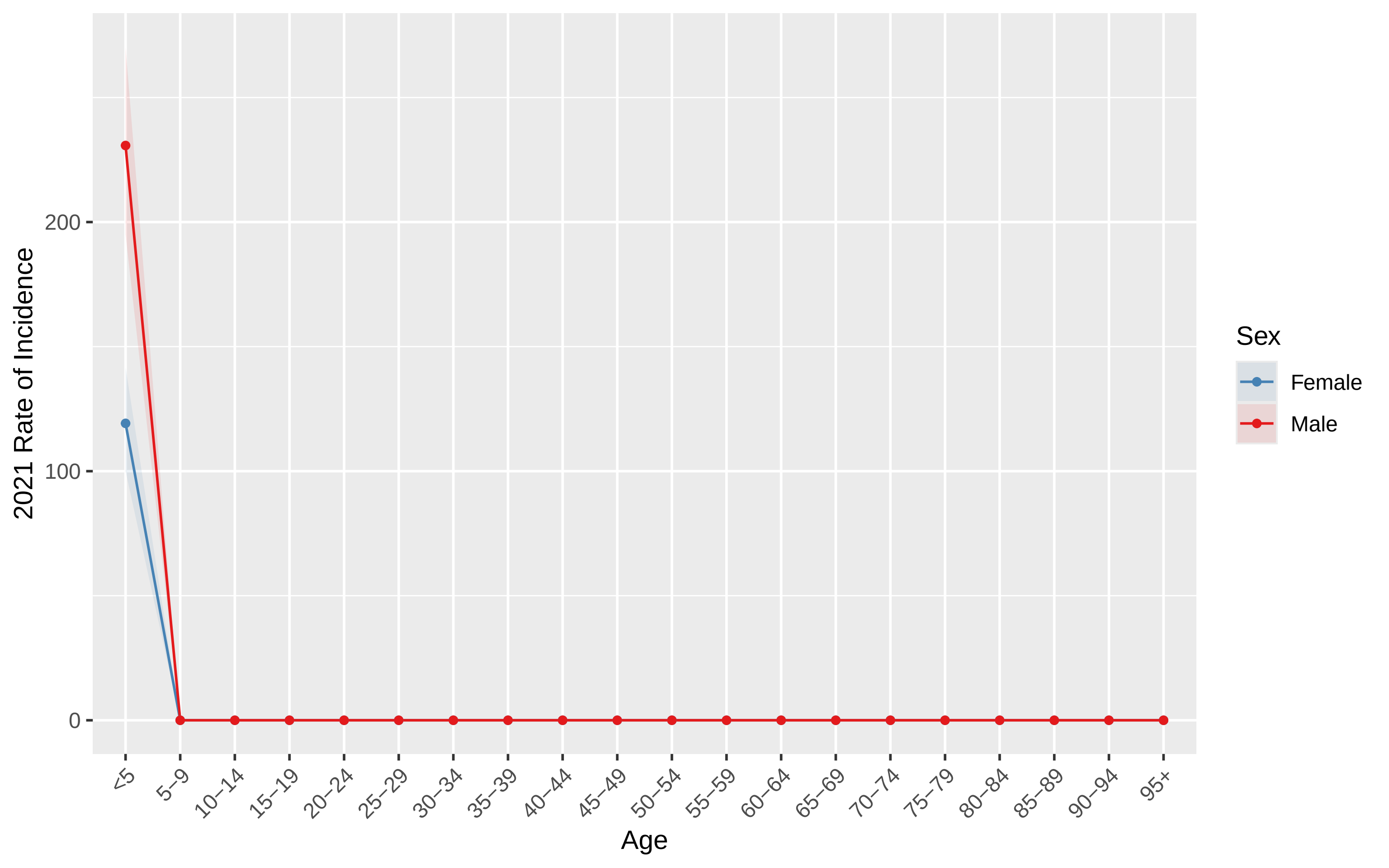
2021 Rate of Incidence
200
100
0
Age
<5
5–9
10–14
15–19
20–24
25–29
30–34
35–39
40–44
45–49
50–54
55–59
60–64
65–69
70–74
75–79
80–84
85–89
90–94
95+
Sex
Female
Male

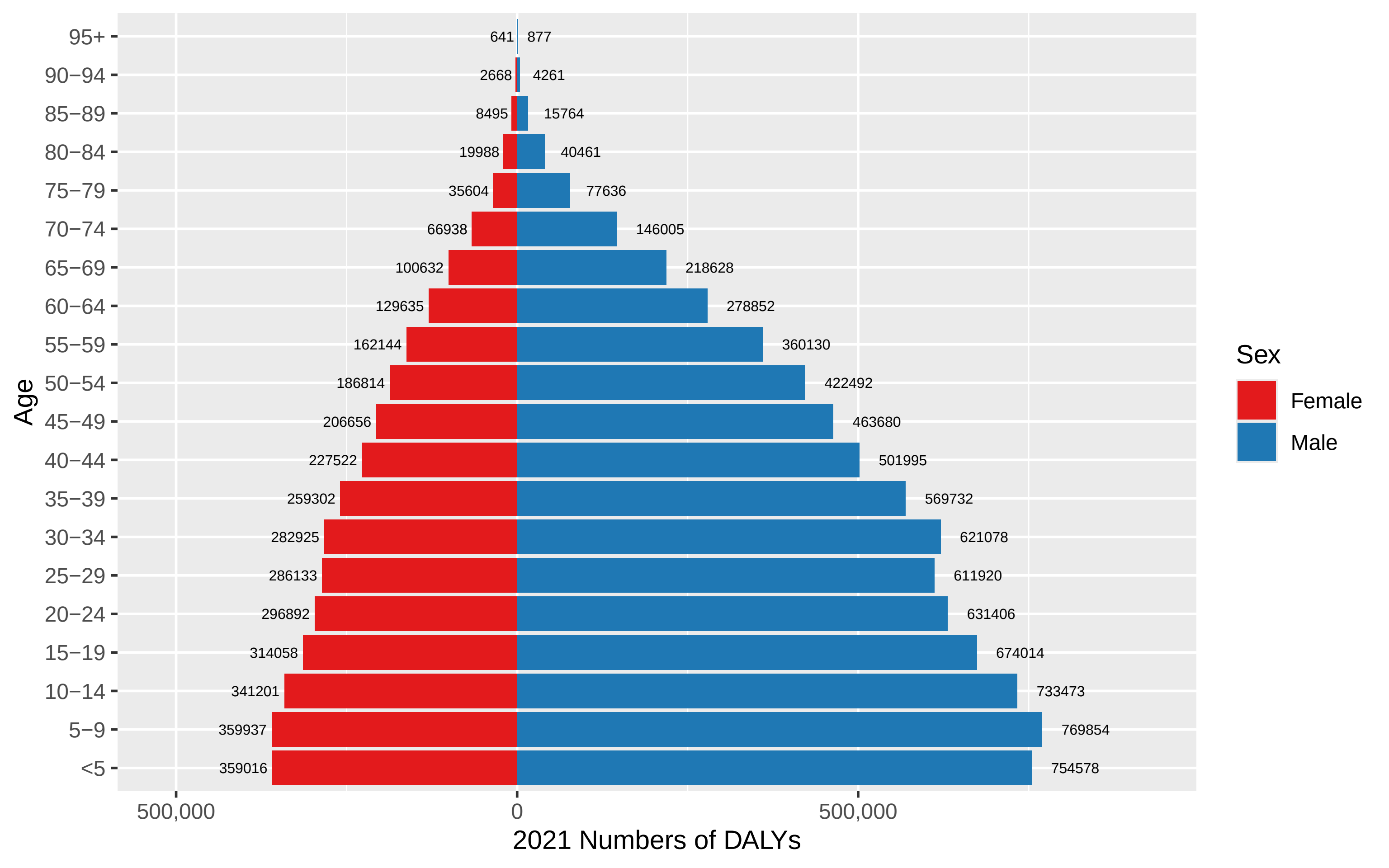

95+
90−94
85−89
80−84
75−79
70−74
65−69
60−64
55−59
50−54
45−49
40−44
35−39
30−34
25−29
20−24
15−19
10−14
5−9
<5
Age
641
877
2668
4261
8495
15764
19988
40461
35604
77636
66938
146005
100632
218628
129635
278852
162144
360130
186814
422492
206656
463680
227522
501995
259302
569732
282925
621078
286133
611920
296892
631406
314058
674014
341201
733473
359937
769854
359016
754578
500,000
0
500,000
2021 Numbers of DALYs
Sex
Female
Male

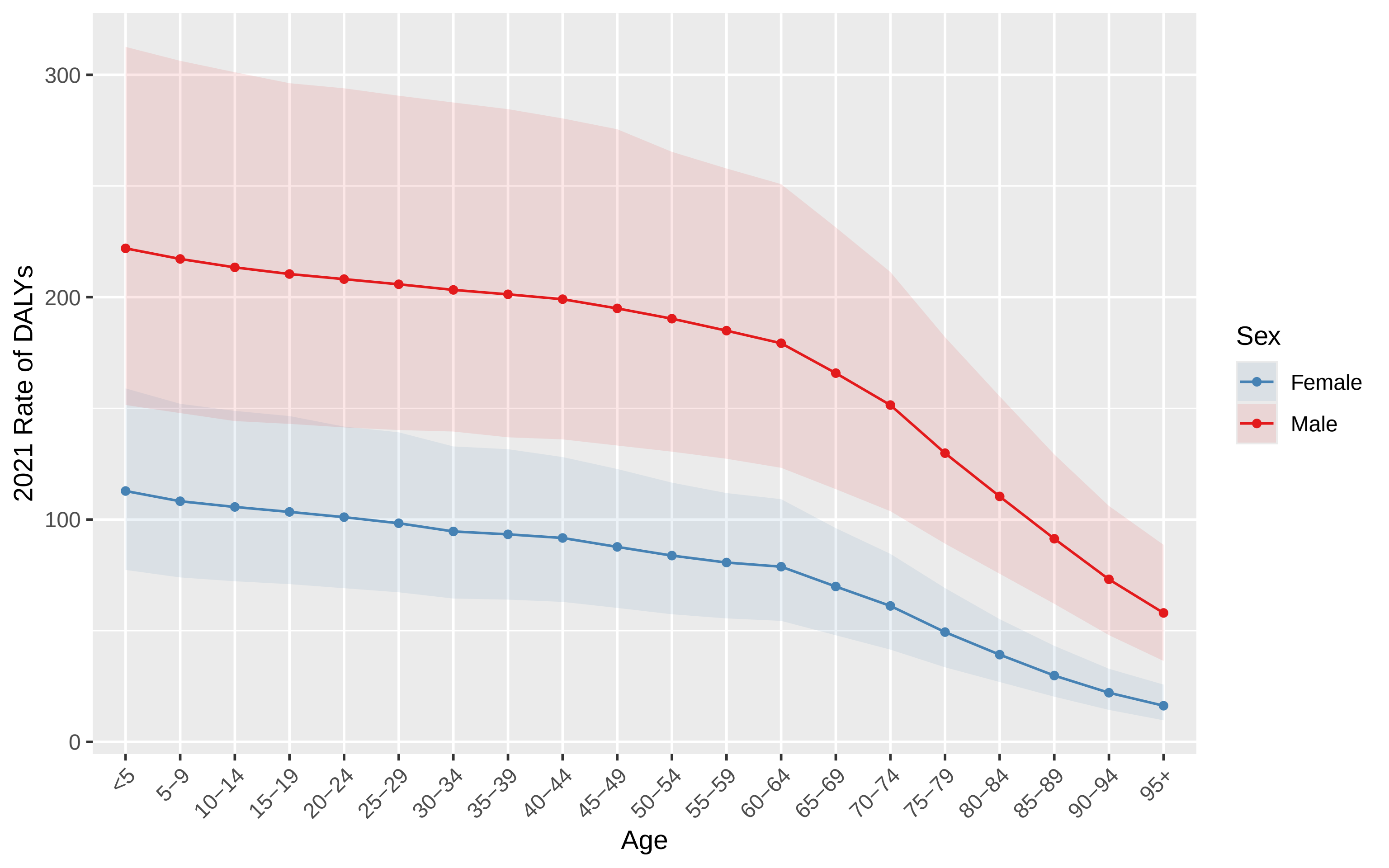

2021 Rate of DALYs
300
200
100
0
<5
5–9
10–14
15–19
20–24
25–29
30–34
35–39
40–44
45–49
50–54
55–59
60–64
65–69
70–74
75–79
80–84
85–89
90–94
95+
Age
Sex
Female
Male

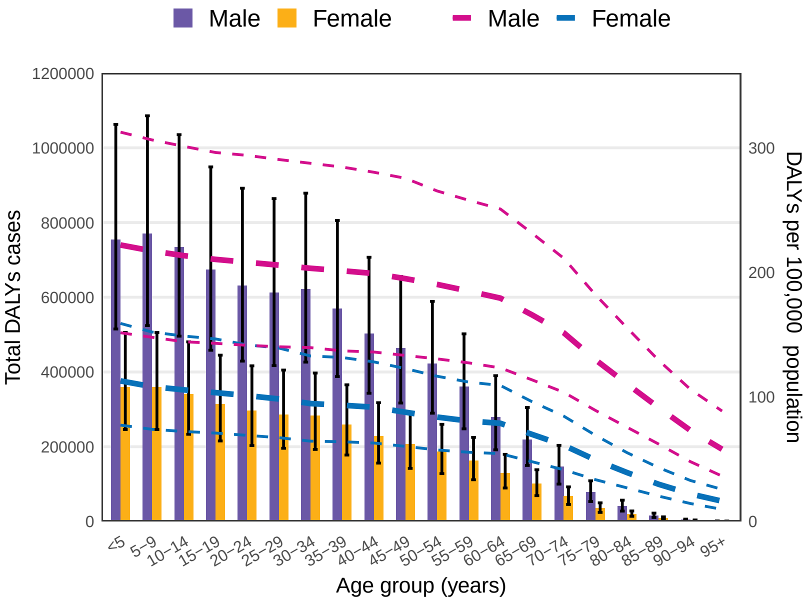

Male
Female
Male
Female
Total DALYs cases
DALYs per 100,000 population
Age group (years)
1200000
1000000
800000
600000
400000
200000
0
300
200
100
0
<5
5–9
10–14
15–19
20–24
25–29
30–34
35–39
40–44
45–49
50–54
55–59
60–64
65–69
70–74
75–79
80–84
85–89
90–94
95+

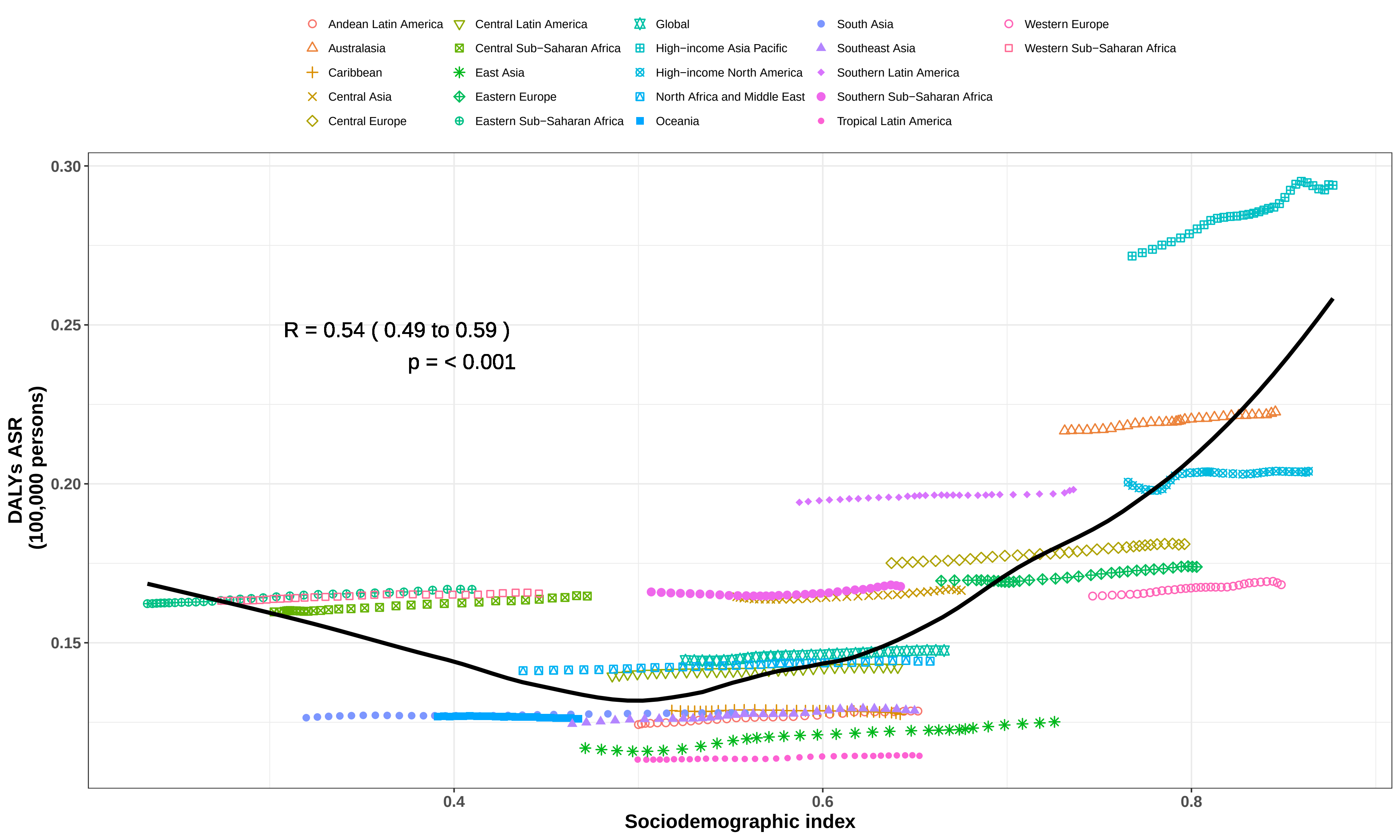

Andean Latin America
Australasia
Caribbean
Central Asia
Central Europe
Central Latin America
Central Sub−Saharan Africa
East Asia
Eastern Europe
Eastern Sub−Saharan Africa
Global
High−income Asia Pacific
High−income North America
North Africa and Middle East
Oceania
South Asia
Southeast Asia
Southern Latin America
Southern Sub−Saharan Africa
Tropical Latin America
Western Europe
Western Sub−Saharan Africa
R = 0.54 (0.49 to 0.59)
p = < 0.001
DALYs ASR
(100,000 persons)
0.30
0.25
0.20
0.15
0.4
0.6
0.8
Sociodemographic index

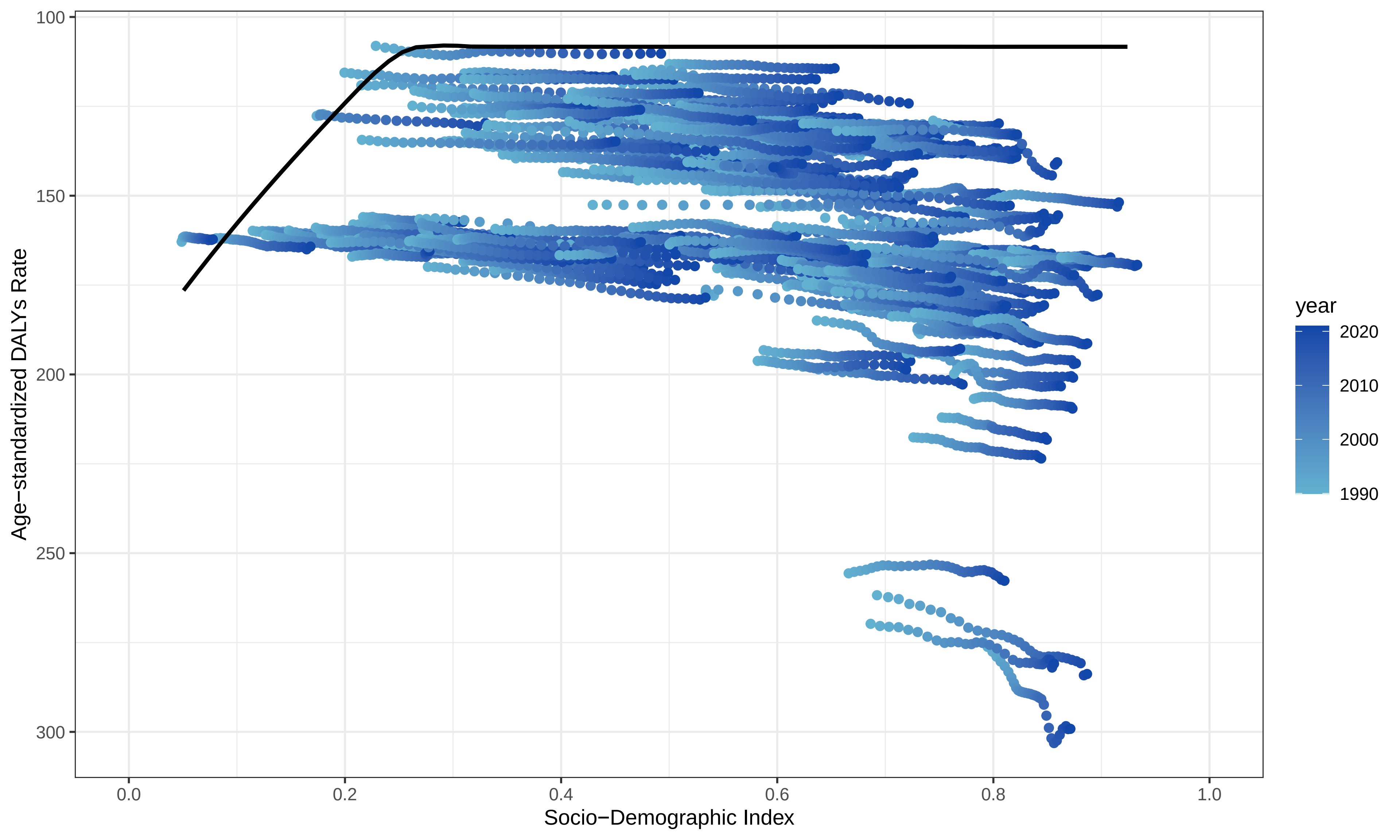

100
150
200
250
300
Age−standardized DALYs Rate
0.0
0.2
0.4
0.6
0.8
1.0
Socio−Demographic Index
year
2020
2010
2000
1990

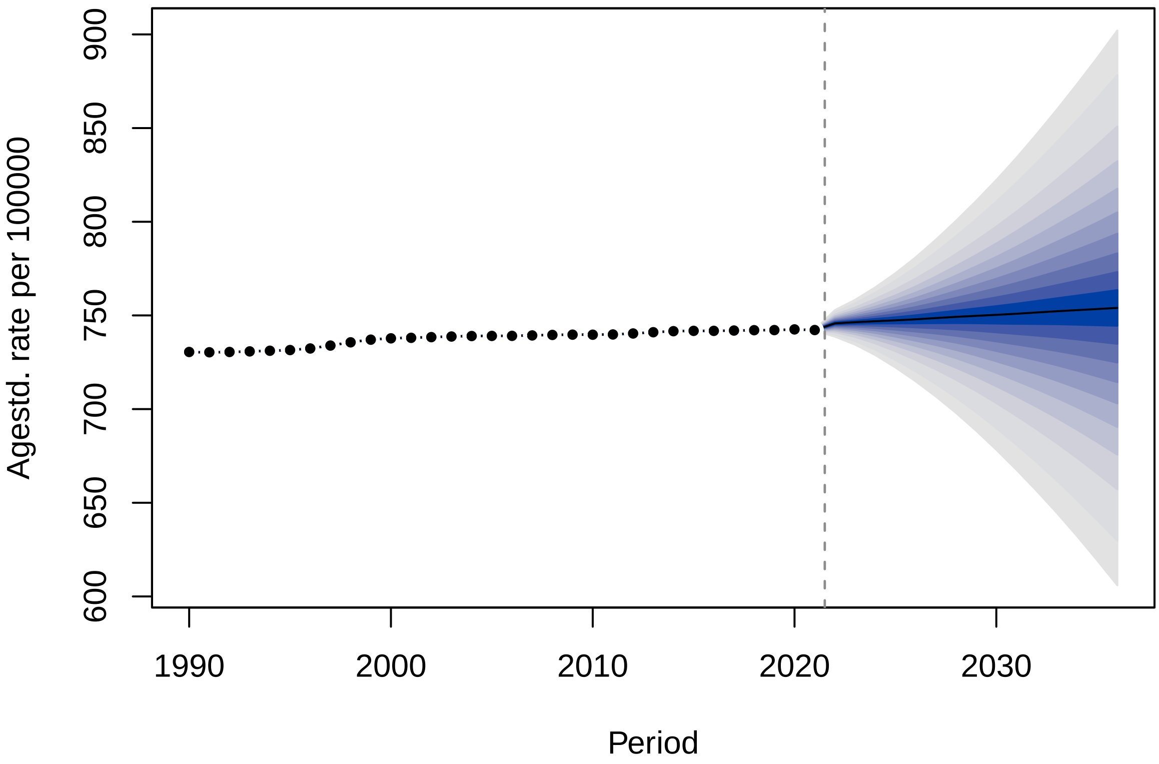

Agestd. rate per 100000
900
850
800
750
700
650
600
1990
2000
2010
2020
2030
Period

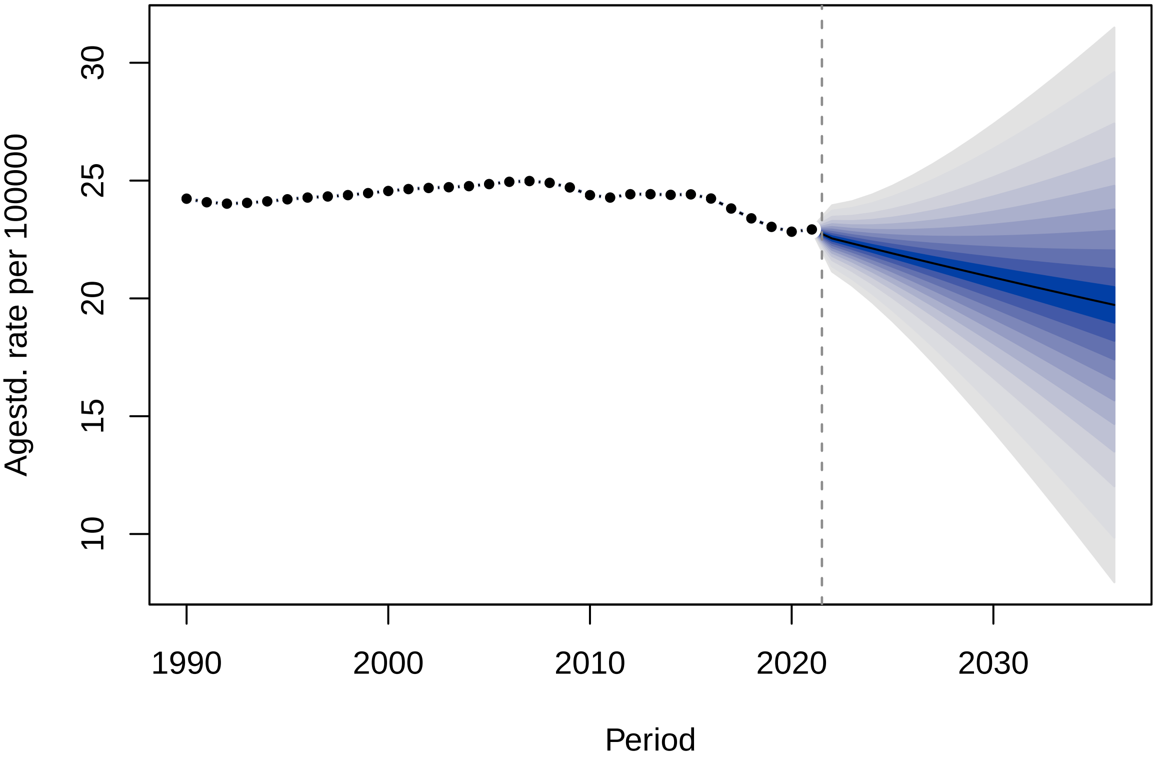

Agestd. rate per 100000
30
25
20
15
10
1990
2000
2010
2020
2030
Period

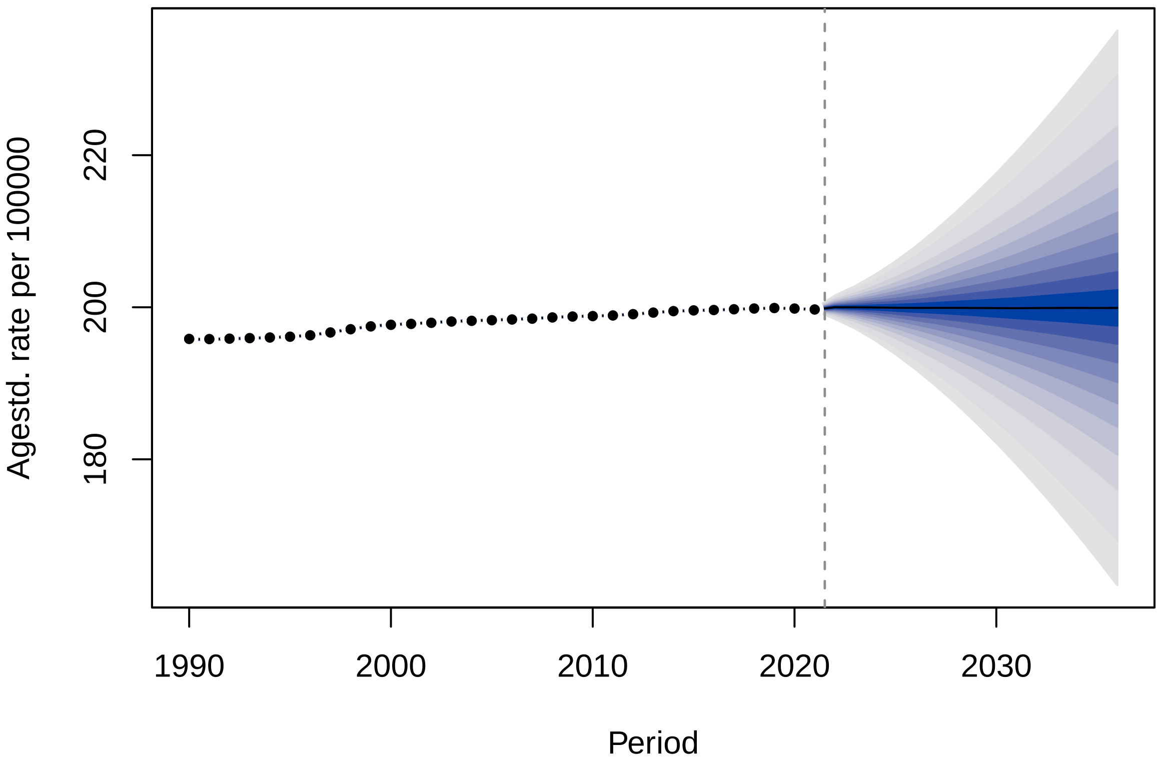

Agestd. rate per 1000000
180
200
220
1990
2000
2010
2020
2030
Period

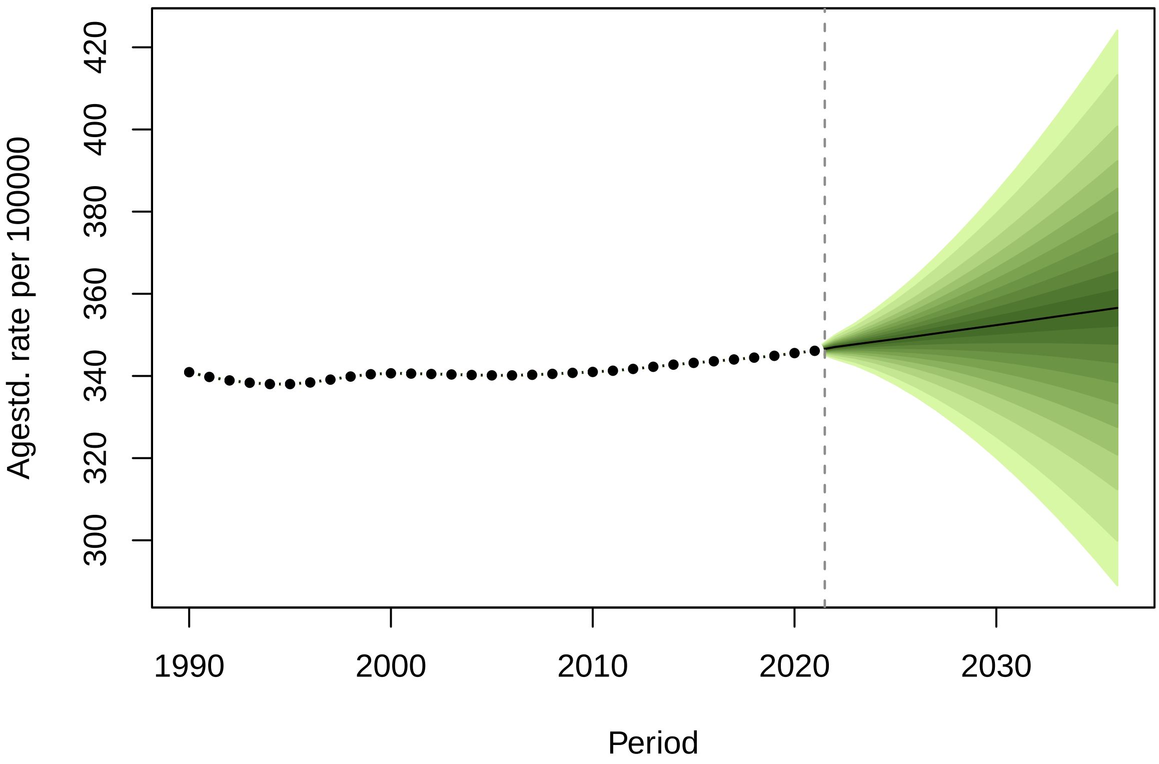

Agestd. rate per 1000000
420
400
380
360
340
320
300
1990
2000
2010
2020
2030
Period

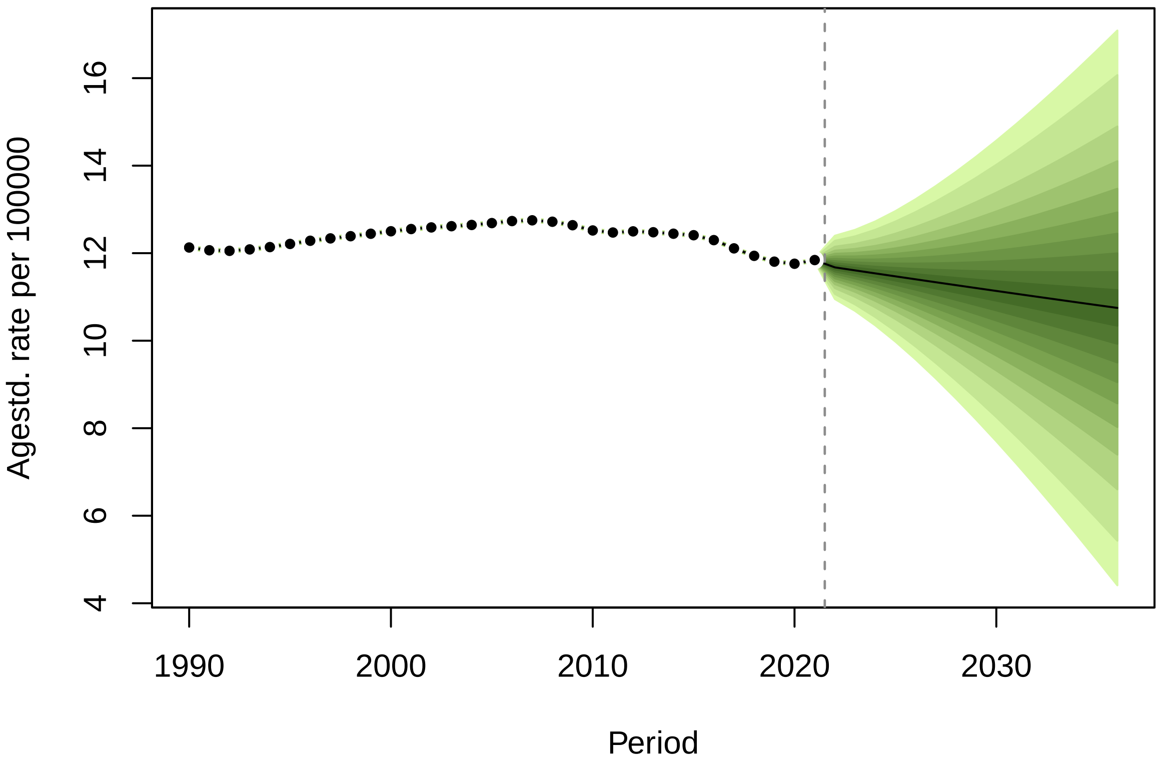

Agestd. rate per 1000000
4
6
8
10
12
14
16
1990
2000
2010
2020
2030
Period

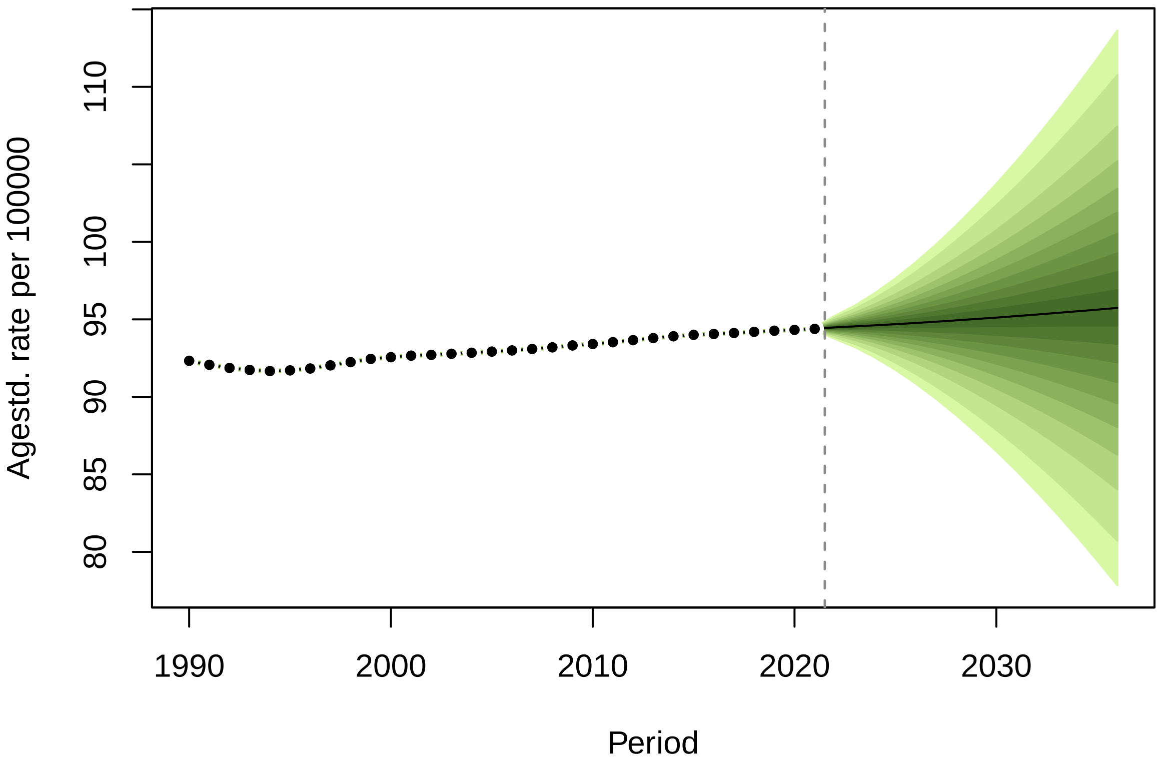

Agestd. rate per 1000000
80
85
90
95
100
110
1990
2000
2010
2020
2030
Period

Location	1990 No (95% UI)	1990 ASRs per 100, 000 (95% UI)	2021 No (95% UI)	2021 ASRs per 100, 000 (95% UI)	Percentage change in the ASRs per 100, 000
Australia	194956 (161736,231515)	1166.6 (968.2,1385.3)	298553 (248636,355954)	1195.4 (996.1,1428.7)	2.5 (-6.8,11.1)
New Zealand	38633 (32090,45962)	1140 (947.2,1356.2)	58774 (48727,70501)	1169.2 (969.9,1404.3)	2.6 (-4.8,9.1)
Antigua and Barbuda	428 (356,505)	705.1 (586.3,833)	627 (518,744)	711.6 (588,844.9)	0.9 (-6.9,9.5)
Barbados	1768 (1472,2086)	706.4 (588.3,833.5)	2029 (1683,2431)	712.4 (593.8,854.6)	0.8 (-6.7,10.4)
Belize	1330 (1100,1581)	684.3 (567.9,813.9)	2986 (2484,3592)	683 (567.1,821.9)	-0.2 (-7,6.5)
Bermuda	429 (355,513)	727.2 (602.1,868)	443 (366,534)	740.5 (611.9,893.6)	1.8 (-5.9,9.4)
Dominican Republic	49487 (41205,59262)	665.4 (553.3,799.7)	74959 (62615,89409)	677.1 (565.5,807.5)	1.7 (-7.5,9.1)
Grenada	606 (504,725)	684.1 (568.6,818)	712 (590,843)	697.9 (578.4,825.5)	2 (-5.7,9.8)
Jamaica	16866 (14158,19940)	700.1 (587.2,828.8)	19465 (16351,23130)	702.5 (590.2,834.7)	0.4 (-7.3,7)
Puerto Rico	26066 (21486,31117)	721.8 (594.8,861.6)	22419 (18496,26891)	731.1 (602.8,877.6)	1.3 (-7.3,10.7)
Saint Kitts and Nevis	287 (236,346)	683 (562.1,825.9)	403 (337,478)	694.9 (581.7,824.3)	1.7 (-7.2,11)
Saint Lucia	971 (807,1164)	689.1 (573.1,827.3)	1214 (1004,1452)	702.4 (581.6,837.4)	1.9 (-6.3,9.1)
Saint Vincent and the Grenadines	775 (645,919)	686 (570.5,816.4)	778 (646,927)	693.7 (575.6,825.8)	1.1 (-6.7,10.1)
United States Virgin Islands	756 (633,898)	702.4 (587.6,834.2)	567 (469,675)	709.9 (588.3,848.5)	1.1 (-6.6,9.3)
Georgia	49434 (41136,59053)	902.3 (751.2,1077.7)	31347 (26097,37274)	910.7 (760.4,1082.5)	0.9 (-6.3,8.8)
Mongolia	19165 (15935,22558)	847.2 (704.1,997.6)	29425 (24829,35121)	861.3 (726.4,1027.4)	1.7 (-6.2,8.5)
Turkmenistan	33429 (28076,39571)	864.9 (725.8,1024.7)	46417 (38815,54767)	884.6 (740.3,1043.2)	2.3 (-5.7,10.6)
Bosnia and Herzegovina	43123 (35887,50697)	951.1 (791.6,1118.6)	30578 (25322,36194)	972 (806.8,1150)	2.2 (-5.9,10.6)
Hungary	95236 (79699,112669)	942.2 (787.9,1115.4)	87803 (72633,104569)	971 (803.5,1153.1)	3.1 (-4.8,11)
Montenegro	6037 (5035,7210)	963.3 (803.5,1150.3)	5858 (4865,6893)	976.3 (811.1,1150)	1.4 (-5.1,9.6)
North Macedonia	18691 (15648,22053)	933.5 (781.4,1101)	20305 (17006,24110)	957.5 (803,1136.9)	2.6 (-5.8,11.4)
Romania	210327 (176039,249143)	910.1 (762,1077.3)	167952 (140239,198085)	939 (786,1105.1)	3.2 (-5.5,11.4)
Central African Republic	24766 (20659,29370)	846.6 (706.4,1003.1)	49603 (41732,60053)	848.7 (711.7,1026.1)	0.3 (-7.6,8)
Democratic Republic of the Congo	352737 (295645,419465)	865.4 (722,1028.8)	844114 (703189,1006901)	887.7 (738.2,1055.6)	2.6 (-4.7,13.1)
Democratic People's Republic of	143960	686.1 (571,815.3)	183546 (152523,219567)	706.1 (587.1,845)	2.9 (-5.4,11.6)

Korea	(119718,170949)				
Taiwan (Province of China)	179069 (149586,212375)	868.8 (725.4,1030)	208211 (171450,247999)	911.1 (752,1083.9)	4.9 (-4,13.4)
Republic of Moldova	39947 (33239,47513)	895.3 (744.8,1064.6)	31504 (26231,37071)	917.9 (766,1082.3)	2.5 (-3.9,10.9)
Russian Federation	1342411 (1129810,1588282)	902.2 (759,1067.8)	1286225 (1079417,1526745)	928.8 (780,1100.6)	3 (-0.2,6.1)
Ukraine	468573 (391469,553634)	912.9 (764,1078.5)	374354 (311842,446635)	922.3 (770.6,1101.3)	1 (-5.6,9)
United Republic of Tanzania	241370 (202090,288516)	879.7 (736.6,1049.7)	550748 (460151,654251)	897.5 (749.1,1067.5)	2 (-5.9,11.7)
Brunei Darussalam	3662 (3050,4305)	1359.1 (1129.1,1600.2)	6268 (5277,7473)	1370.5 (1153.1,1635.4)	0.8 (-8.9,8)
Japan	1797367 (1514035,2116209)	1458.6 (1229.7,1714.7)	1846133 (1544551,2177718)	1586.9 (1333.2,1864.1)	8.8 (6.1,11.9)
Republic of Korea	630083 (530085,741854)	1391.8 (1170.7,1640.4)	746098 (619301,882760)	1506.8 (1254,1779.2)	8.3 (-0.4,16.8)
Canada	295882 (246053,351690)	1099.6 (915.2,1307.2)	400293 (333013,471587)	1117.9 (930.4,1317.6)	1.7 (-6.8,9.7)
Greenland	577 (485,687)	1008.2 (847.2,1197.2)	565 (470,667)	1016.9 (848.6,1199.3)	0.9 (-7.3,9.2)
United States of America	2667576 (2253831,3150947)	1069.1 (904.5,1261.3)	3491378 (2919750,4128390)	1094.8 (920.2,1291.2)	2.4 (0,4.4)
Palestine	16507 (13809,19660)	766.9 (640.6,915.1)	41421 (34527,49407)	782.1 (651.4,934.1)	2 (-5.8,11.6)
Syrian Arab Republic	101374 (84015,121565)	761.4 (632.3,913.3)	107525 (89279,129371)	759 (630,913)	-0.3 (-7.8,11.4)
United Arab Emirates	16390 (13614,19767)	833.1 (690.2,1003.5)	85811 (71622,101887)	840.9 (701,999.6)	0.9 (-8.5,10.1)
American Samoa	358 (299,425)	711.1 (592.8,847.5)	355 (294,424)	708.9 (588.9,847.7)	-0.3 (-8.9,7.7)
Cook Islands	141 (117,169)	727.8 (604.7,874.2)	126 (104,151)	724.6 (601.4,867.4)	-0.4 (-7.5,9)
Guam	1035 (861,1241)	736.1 (611.8,884.1)	1134 (937,1363)	726 (600.3,871.4)	-1.4 (-9.2,6.8)
Northern Mariana Islands	339 (283,403)	733.4 (609,868.6)	356 (296,424)	734.2 (611.6,874.8)	0.1 (-5.8,8.8)
Solomon Islands	2410 (2007,2888)	675.8 (562.4,810.9)	4774 (4000,5681)	671.8 (562.7,801.6)	-0.6 (-8.5,6.9)
Tokelau	11 (9,14)	698.5 (583.4,833.6)	10 (8,12)	714.5 (598.7,855.6)	2.3 (-5.6,11.7)
Tuvalu	64 (54,77)	653.6 (544.9,783.7)	86 (71,103)	684.4 (566.4,820.7)	4.7 (-4.1,14.7)
Lao People's Democratic Republic	28274 (23548,33649)	643.4 (535.8,764.5)	50662 (42266,60801)	668.8 (557.4,802.7)	3.9 (-3.4,12)
Malaysia	129759 (108016,154305)	711.4 (592.4,846.4)	235032 (195861,280303)	734.3 (611.9,874.6)	3.2 (-5,11.6)
Ireland	36948 (30804,44058)	1034.3 (862.3,1234)	51255 (42768,60994)	1071.9 (894.8,1274.4)	3.6 (-5.7,13.3)
Burkina Faso	87805 (73835,104346)	863.6	211002 (177560,248825)	875.7 (736.9,1034)	1.4 (-6.1,9.2)

		(727.6,1027.3)			
People's Republic of China	7347264 (6120001,8699968)	612.6 (509.8,725)	9078613 (7559642,10816937)	655.7 (545.1,780.4)	3.4 (-5.9,12.5)
Democratic Republic of Timor-Leste	5503 (4605,6547)	677.5 (568.4,809.7)	9752 (8149,11610)	677.3 (562,808.1)	1.3 (-7.1,10.1)
Kingdom of Thailand	406111 (336913,487825)	650 (539.5,777.7)	464142 (384301,556047)	647.3 (537.6,772.5)	5.7 (-3.8,15.9)
Socialist Republic of Viet Nam	486268 (400306,578674)	685.8 (573.4,817.9)	728725 (608388,866947)	684.8 (566.4,818.4)	-0.3 (-8,9.3)
Democratic Socialist Republic of Sri Lanka	128014 (105636,152382)	687.4 (565.7,818.7)	165139 (136817,197366)	726.3 (606.3,863.6)	1.8 (-8.6,9.8)
Republic of Armenia	31561 (26458,37485)	698.5 (578.7,839)	26787 (22228,31693)	722.2 (597.6,864.5)	1 (-0.9,2.8)
Republic of the Philippines	446232 (375503,529658)	864 (719.9,1029)	792254 (665372,936397)	866.8 (726.9,1023.6)	2.7 (-5.9,10.5)
Republic of Vanuatu	1085 (897,1289)	668.4 (558.6,794)	2176 (1802,2605)	677.3 (566.1,807.2)	4.1 (-4.3,13.6)
Republic of the Union of Myanmar	273455 (227001,326755)	872.1 (729.4,1040.2)	381152 (316476,457387)	888.8 (746,1060.8)	-0.4 (-8,7)
Republic of Maldives	1615 (1342,1935)	880 (734.5,1057.8)	4089 (3364,4872)	897.7 (752.8,1064.9)	0.8 (-6.3,8.6)
Kingdom of Tonga	711 (588,846)	730.1 (602.3,870.3)	754 (624,899)	749.9 (620.3,896)	0.3 (-7.2,7.1)
Independent State of Samoa	1226 (1014,1459)	650.3 (540.2,778.5)	1524 (1271,1814)	668.8 (555.6,803)	0.8 (-5.9,8.1)
Republic of Uzbekistan	190129 (158446,225568)	872.2 (726.4,1035.7)	306208 (255218,361269)	893 (745.1,1051.2)	2.2 (-4.5,10.8)
Republic of Albania	31026 (26155,36829)	907.3 (761.6,1078.7)	24352 (20150,28733)	922 (766.6,1090.5)	3.6 (-5.3,11.9)
Republic of Indonesia	1213580 (1013694,1434441)	679.6 (573.4,806.9)	1820130 (1528694,2163015)	686.1 (576.4,811.2)	1.7 (-7.4,10.8)
Kingdom of Cambodia	70004 (58503,84033)	698.5 (580.6,838)	117092 (97585,140257)	765.9 (630,912.2)	3.8 (-3.2,12.6)
Federated States of Micronesia	737 (614,878)	631.2 (526.9,746.5)	717 (603,846)	647.3 (543.6,769)	0 (-7.9,7.3)
Republic of Bulgaria	78307 (65612,94198)	641.6 (536.7,770.1)	59412 (49351,70647)	672.4 (560.6,805.8)	0.6 (-6.4,8.2)
Independent State of Papua New Guinea	28911 (24145,34649)	696.3 (574.8,828)	72756 (61047,87244)	696.9 (580.5,829.9)	0.1 (-8.3,8.7)
Republic of Croatia	46323 (38654,54776)	672.7 (561.7,804.6)	39608 (32808,47258)	669.8 (562,804.3)	2.4 (-5.3,10.1)
Republic of Tajikistan	48444 (40372,57829)	680.3 (563,809.8)	90860 (76072,107163)	671.1 (555.5,802.4)	2 (-5.7,9.9)
Republic of the Marshall Islands	325 (272,388)	692.7 (572.4,823.2)	392 (326,467)	694.9 (575.6,829.3)	0.3 (-7.1,8.6)
Republic of Kiribati	509 (424,608)	932 (775,1093.2)	814 (677,973)	968.4 (801.4,1155.4)	0 (-7.8,7.9)
Republic of Kazakhstan	147271 (122840,176991)	976.3 (815.3,1150.3)	171145 (143571,203083)	1017.9 (845.7,1224.1)	0.7 (-6.5,10.5)
Republic of Fiji	5406 (4522,6438)	679.3 (566.3,809.4)	6422 (5310,7678)	683.2 (575,804)	-0.1 (-8.1,7.9)

Republic of Azerbaijan	65698 (54706,77962)	935.2 (787.6,1111.7)	93774 (78203,110267)	964.5 (806.7,1144.4)	0 (-8,7.9)
Kyrgyz Republic	40145 (33573,47838)	942.6 (781,1110.1)	62364 (52333,74525)	966.7 (809,1150.8)	3.5 (-3.4,12.6)
Republic of Belarus	94187 (78559,111908)	979.6 (823.7,1157.2)	84113 (69832,100487)	999.3 (839.6,1186)	0 (-7.3,8.8)
Republic of Estonia	14203 (11916,16881)	916 (772.4,1086.8)	11867 (9951,14118)	944 (781.6,1112.3)	0.9 (-7.3,10)
Republic of Slovenia	18961 (15814,22353)	872.7 (725.8,1036)	19987 (16579,24003)	879.4 (733.2,1035.4)	1 (-7.1,9.9)
Slovak Republic	49394 (40880,58194)	1429.2 (1199.7,1698.5)	50365 (42150,60085)	1487.3 (1250,1767.8)	2 (-1.2,5.6)
Republic of Serbia	89176 (74170,104678)	966.7 (806.6,1142.1)	82418 (67984,98177)	996 (824.9,1186.2)	3.1 (-1.1,6.9)
Republic of Poland	353459 (297907,420246)	925.1 (776.2,1114.3)	350686 (292313,416249)	936.7 (782.9,1115.1)	1.4 (-7,10.6)
Republic of Singapore	44192 (37095,52496)	962 (797.3,1138.4)	83478 (70079,99156)	996.7 (824.2,1172.9)	1.8 (-0.6,4.4)
Republic of Finland	40024 (33355,47868)	794.9 (664.1,943.6)	42267 (34965,50426)	795.1 (665.4,944)	1.6 (-6.5,10)
Principality of Andorra	496 (412,592)	887.5 (742.2,1060.3)	743 (617,885)	901.8 (749.7,1081.9)	1.6 (-7,9.3)
Republic of Austria	65473 (54497,77607)	762.8 (635.6,908.1)	75244 (62297,89954)	786.6 (652.9,931.7)	3 (-6.4,11.5)
Czech Republic	96907 (80255,114752)	918.4 (768.3,1095.6)	100751 (83247,118532)	943.2 (778.7,1117.1)	1.3 (-6.3,8.9)
Hellenic Republic	89003 (74727,105660)	928.8 (768,1098.8)	82360 (68115,98856)	944.8 (783.9,1115.8)	-0.4 (-8.1,8.5)
Federal Republic of Germany	671042 (562926,804755)	827.9 (691,989.2)	709083 (583887,846724)	828.7 (687.5,990.5)	1 (-8.2,10.3)
Portuguese Republic	83441 (69839,99799)	872.5 (729.2,1036.7)	83820 (69544,99210)	881.3 (734.9,1049.9)	1.7 (-6.8,10.2)
French Republic	487796 (407831,579420)	875.3 (737.9,1045.7)	547708 (455312,652227)	882.9 (733.7,1048)	0.6 (-7.8,9.4)
Republic of Malta	3240 (2696,3852)	776.7 (644.3,932.5)	3704 (3094,4423)	785 (650.9,932.3)	3 (-5.4,12.1)
Grand Duchy of Luxembourg	3273 (2738,3910)	724.6 (601.5,867.3)	5594 (4662,6713)	726.6 (608.1,859.7)	9.7 (-1.7,20.8)
Republic of Iceland	2590 (2178,3081)	914 (760.1,1092.8)	3547 (2960,4182)	905.9 (752.9,1077.5)	0.3 (-8.5,7)
Eastern Republic of Uruguay	32217 (27042,38370)	879.3 (737.7,1053.2)	34505 (28690,41437)	897.5 (738.7,1071)	2.1 (-6.7,11)
Kingdom of Belgium	84343 (70594,100897)	1028.7 (865.6,1223.8)	95502 (79179,113504)	1047.3 (875.1,1235)	4.3 (-6.9,16.4)
Republic of Chile	141563 (118050,166519)	884.3 (736.4,1050.7)	198698 (165530,234571)	898.1 (751.3,1069.8)	-0.1 (-3.6,3.9)

Kingdom of Denmark	45191 (38275,53453)	809.3 (682,954.4)	52193 (43800,61841)	808.3 (677.1,963.1)	1.9 (-6.5,11.1)
Republic of Latvia	23773 (19847,28378)	984 (813.5,1197.8)	16377 (13504,19373)	1016.7 (839.5,1216.8)	3.3 (-3.8,9.3)
Republic of Lithuania	33550 (27741,39695)	883.7 (742.1,1047)	23950 (19818,28268)	881.4 (732.5,1056.8)	2.6 (-6.2,11.9)
Republic of Haiti	42181 (35138,50624)	926.4 (777.8,1100.5)	83918 (69937,99848)	964.5 (811.2,1146.7)	4.1 (-4.6,12.8)
Republic of Cyprus	6793 (5724,8116)	687.6 (575.5,813.1)	11580 (9600,13763)	750.8 (631.6,890.3)	3 (-5.6,12.1)
Republic of Guyana	5414 (4469,6471)	1046.4 (871.6,1231.6)	5142 (4284,6116)	1082.6 (902.8,1277)	4.3 (-4.6,13.4)
Republic of Panama	18771 (15592,22289)	878.4 (732.4,1040.6)	33372 (28006,39445)	892.2 (741.1,1069.1)	2.7 (-5.2,10.6)
Argentine Republic	342394 (285559,409124)	849.2 (706,1010.9)	467333 (387115,549821)	927.4 (773.1,1096.9)	0.1 (-8.2,8)
Republic of Costa Rica	24663 (20404,29587)	886.8 (744.6,1048.7)	37148 (30730,44470)	905.4 (756.3,1079.4)	0.8 (-8.1,8.7)
Kingdom of Spain	370330 (309281,439125)	867.7 (725.6,1037.9)	435737 (366879,512824)	896.3 (750.2,1059.9)	0.8 (-7.2,8.8)
Swiss Confederation	59214 (49019,70607)	847.1 (709.2,1013.9)	75957 (62946,90332)	861.8 (718.3,1019.8)	2.8 (-4.4,11.3)
Republic of Italy	534487 (449992,631992)	1030.1 (859,1231)	548536 (460422,649272)	1045.7 (867.3,1230.9)	2 (-4.3,9.7)
Kingdom of Norway	32917 (27741,38806)	1043.4 (875.9,1243.3)	41725 (34854,49755)	1058.1 (880.2,1268.2)	1.8 (-6.1,10.3)
Republic of Peru	149507 (125268,177563)	709.2 (587.5,852.7)	251558 (210109,300501)	733.9 (607.9,873.3)	7 (3,11.1)
Kingdom of the Netherlands	130285 (107750,154426)	882 (730.5,1052.8)	146299 (120487,174361)	900.1 (749.7,1067.6)	-0.1 (-7.1,8)
Commonwealth of the Bahamas	1832 (1517,2201)	874.9 (727.8,1039.9)	2673 (2208,3176)	868.1 (726,1020.8)	0.9 (-7.6,10.1)
Republic of Cuba	78021 (64693,92298)	918.5 (778.1,1086.1)	77815 (64831,92479)	946.5 (797.2,1124.2)	-0.9 (-9.3,7.6)
Commonwealth of Dominica	509 (423,606)	879 (736.3,1050.8)	459 (384,548)	885.3 (734.8,1051.9)	2.6 (-5.4,11.1)
Islamic Republic of Afghanistan	72336 (60253,86235)	982.9 (821.9,1167.5)	234672 (193720,279519)	1024.7 (863.8,1204.3)	2.7 (-5,11.8)
Republic of Paraguay	26053 (21519,30936)	894.5 (740.6,1065.2)	45567 (37819,54286)	902.3 (749.2,1073.8)	-1.4 (-9.2,7.9)
Kingdom of Saudi Arabia	130756 (109553,155236)	884 (733.6,1059.2)	316319 (261846,377152)	894.6 (748,1064.2)	1.6 (-7.5,8.9)
State of Qatar	4319 (3606,5147)	894.6	30335 (25129,36018)	899.6	1.9 (-5.5,9.5)

		(740.7,1060.6)		(739.8,1073.2)	
Plurinational State of Bolivia	43394 (36216,51497)	888.6 (740.9,1051.8)	79586 (66690,95487)	909.1 (752.9,1083.9)	3 (-4.8,11.1)
Republic of Ecuador	69266 (58083,83089)	876.7 (727.5,1038.2)	124017 (102804,147821)	893.1 (744.4,1057.7)	4.8 (-4.2,14.5)
State of Israel	39570 (33067,46975)	893 (742.6,1059.7)	75028 (62800,88989)	898 (752.1,1063.5)	1.5 (-6.5,9)
Kingdom of Sweden	80246 (66356,97503)	917.7 (765,1090.1)	98896 (81105,118051)	945.2 (783.5,1128.1)	3.1 (-5.9,10.7)
United Kingdom of Great Britain and Northern Ireland	377328 (316088,445627)	904.3 (765.2,1080.9)	481779 (404014,573252)	951.6 (792.4,1131.9)	-0.4 (-8.7,6.4)
Federative Republic of Brazil	934492 (777483,1110612)	662.9 (546,791.9)	1335516 (1118732,1591670)	663.2 (552.8,789)	1.4 (-6.2,10.4)
Republic of Suriname	2686 (2231,3202)	692.1 (577.7,826.7)	3873 (3215,4627)	699.2 (585.7,835.9)	0.4 (-8.8,8.3)
Republic of Djibouti	3997 (3348,4783)	625.1 (519.6,749.9)	12141 (10115,14422)	627 (522.7,746.2)	0.2 (-4.7,4.9)
State of Eritrea	31859 (26703,38227)	692.5 (574.1,829.3)	61870 (51571,73194)	692.9 (572.2,823.3)	3.5 (-5.1,12.7)
Republic of Trinidad and Tobago	8538 (7121,10201)	871.8 (724.8,1040.3)	9479 (7921,11319)	887.3 (744.1,1059.9)	2.3 (-5.5,10.2)
Republic of Honduras	36200 (30073,43348)	674.3 (560,803.7)	75743 (63116,89760)	673.7 (559.5,804.7)	-0.8 (-7.8,6.9)
Republic of Nicaragua	30344 (25373,36406)	681.7 (567.8,807.2)	51312 (42553,60565)	672.3 (560.1,802.9)	-1.3 (-9.4,7)
Bolivarian Republic of Venezuela	146652 (122250,175830)	893.2 (746.2,1056.6)	199838 (167417,237716)	890.6 (744.5,1062.5)	2.5 (-6,9.5)
Hashemite Kingdom of Jordan	30936 (25822,36773)	695.4 (577.2,827.4)	101898 (84712,120872)	701.6 (584,827.2)	3.1 (-6.3,12.5)
State of Libya	33671 (28062,39958)	793.5 (663.2,943.9)	53424 (44282,63557)	817.8 (677.7,975.7)	2.4 (-5.1,10.4)
Federal Republic of Somalia	75133 (62671,89365)	689.5 (572.2,822)	203174 (170536,238936)	695 (582.7,829.6)	0.9 (-2.2,3.6)
State of Kuwait	14869 (12312,17791)	741.6 (620.3,889.8)	38953 (32200,46278)	756.1 (627.4,892.4)	3 (-4.6,11.8)
Republic of Guatemala	63386 (52476,75397)	766.1 (635.9,910.2)	117068 (97641,138674)	779.7 (654.4,921.4)	2 (-6.8,10.4)
Kingdom of Morocco	196172 (162992,235172)	621.1 (513.2,738.3)	282540 (237025,335302)	628.1 (521.4,748.2)	3.9 (-0.4,8.9)
Republic of Colombia	252558 (212456,300644)	666.2 (556.1,793.7)	374548 (311161,447535)	691.7 (577.7,826.2)	0.3 (-7.9,9.4)
Republic of Cabo Verde	3339 (2831,3982)	646.6 (538.7,766.9)	5388 (4489,6409)	663.1 (555.1,795.5)	2.2 (-5.4,11.9)
Republic of El Salvador	40417 (33789,48158)	751.4 (632,895.4)	48189 (40115,57745)	771.8 (640.9,921.5)	1.1 (-7.7,10.3)
Republic of Iraq	133932 (109988,159429)	788.6 (652.1,947.3)	300186 (250707,360994)	792 (655.2,946.8)	3.2 (-5.3,11.4)
People's Republic of Bangladesh	666537 (556520,794367)	731.7 (610.1,872.1)	977775 (809418,1157419)	745.6 (620.6,893.8)	0.8 (-7.9,8.1)
Arab Republic of Egypt	431931 (360259,514118)	713.1 (592,849.1)	827877 (692545,982173)	726 (605.5,861)	9.2 (0.3,18.7)
Lebanese Republic	25906 (21693,30733)	727.7 (604.5,873.3)	47951 (39803,56800)	727.8 (605.8,864.2)	1 (-0.6,2.5)

Islamic Republic of Iran	458913 (387004,544339)	745 (626.6,883.1)	677140 (567413,799868)	758.5 (641.3,902.8)	2.1 (-5.8,11)
Republic of Yemen	105404 (87801,126475)	771.1 (651.9,915.7)	255839 (214344,304154)	791.2 (662.9,934)	0.7 (-8.5,9.3)
Republic of Tunisia	66759 (55500,80208)	694.1 (568.7,826.5)	92257 (76510,109576)	709.2 (590.7,852.8)	2.4 (-4.5,10.7)
Sultanate of Oman	16825 (14020,20102)	791.5 (661,943.3)	40757 (33695,48903)	810 (672.7,960.5)	9.2 (6.9,11.5)
Federal Democratic Republic of Nepal	127977 (106473,152739)	846.5 (709,1003.6)	198854 (165182,238529)	872.4 (724.2,1033.2)	2.5 (-5.1,11.2)
Republic of Turkey	452114 (376966,537059)	771.8 (642.9,918.8)	652685 (541549,773512)	769.1 (636.7,915.3)	1.6 (-7.5,10.3)
Islamic Republic of Pakistan	736179 (616472,871153)	750.7 (624.5,900.5)	1547309 (1290751,1819389)	756.7 (634.2,899.5)	1.9 (-6.9,10)
Republic of Angola	95122 (79450,112839)	608.4 (506.1,722.6)	305831 (256806,359178)	614.1 (514.6,731.9)	5.2 (-3.8,14.4)
Republic of the Congo	22154 (18499,26424)	759.2 (632.5,913.8)	50251 (42039,59424)	774.6 (644.9,921.9)	0.1 (-7.4,8.8)
People's Democratic Republic of Algeria	200022 (166706,241034)	815.7 (678.5,964)	346139 (288435,412073)	848.4 (703.7,1009.1)	-1.9 (-8.8,4.7)
Kingdom of Bhutan	4255 (3551,5047)	746 (619.9,893.9)	4965 (4142,5901)	756.2 (634.1,897.5)	1.8 (-6.3,9.4)
Gabonese Republic	9105 (7640,10762)	692.1 (575.2,826.9)	16981 (14176,20233)	703.1 (581.7,839.5)	1.4 (-7.3,10.2)
Republic of Mauritius	8020 (6658,9561)	580.2 (484.4,691.1)	9036 (7492,10706)	588.2 (486.7,696.6)	3.2 (-5.3,12.5)
Republic of India	6260916 (5254094,7405450)	637.3 (532,757)	10120042 (8503425,11876619)	648.1 (540.6,770.4)	3.9 (-5.4,13)
United Mexican States	662985 (555619,785858)	630.1 (527.2,746.6)	980052 (828327,1166731)	631.1 (526.6,741.9)	1.6 (-6.1,9.1)
Republic of Kenya	221155 (185458,260230)	865.6 (722.7,1040.4)	474307 (398522,555166)	886.8 (738.3,1053.8)	4.1 (-5.7,13.9)
Republic of Burundi	51108 (42748,60772)	668 (560.3,803.2)	123763 (103230,145723)	682.7 (565.9,813.8)	1.8 (-7.3,10.5)
Republic of Mozambique	122539 (102315,146678)	717.9 (595.1,849.4)	289253 (241019,343910)	723.4 (603.2,859.9)	2.6 (-1.3,6.3)
Republic of Seychelles	525 (435,630)	747.7 (621.7,890.3)	771 (639,917)	763.7 (639.6,905.8)	1.7 (-6.2,10.1)
Republic of Rwanda	66448 (55421,78980)	840.6 (694.2,1006.7)	123933 (103559,146886)	829.5 (685.6,984.3)	4 (-5.4,14.2)
Republic of Namibia	13093 (10931,15560)	926.7 (773.1,1106.2)	22796 (19064,27185)	964.6 (798,1145.1)	0.4 (-7.1,8.6)
Union of the Comoros	4357 (3638,5121)	814.1 (677.2,974.1)	6943 (5795,8222)	832.9 (687.4,998.6)	2.5 (-0.4,5.9)
Republic of Equatorial Guinea	3846 (3206,4559)	867.8 (724.4,1030.3)	14881 (12415,17591)	879.2 (741,1029.7)	2.1 (-4.8,10.3)
Republic of Madagascar	111477 (93176,132636)	702.6 (589.7,830.5)	267842 (223705,317606)	708.1 (595.1,831.2)	2.1 (-1.5,5.6)
Federal Democratic Republic of Ethiopia	467650 (394757,550491)	887.7 (739.2,1042.1)	1026009 (860805,1209152)	906.9 (757.5,1074.5)	1.3 (-6.5,10.4)
Republic of Malawi	91231 (75905,109257)	897.6 (755.2,1057)	182220 (152822,215383)	906.3 (761.4,1061)	2 (-6.9,10.6)

Republic of Zimbabwe	97489 (81522,115928)	861.8 (720.4,1030.3)	143825 (120090,171167)	872.6 (725,1039)	2.3 (-5.8,11.4)
Republic of the Niger	74797 (62273,89565)	890.7 (742.2,1057.6)	235100 (195619,278660)	873.9 (728.9,1041.4)	1.7 (-6.4,9.9)
Republic of Zambia	74097 (61614,88699)	623.7 (518.1,743.8)	184523 (153717,218566)	626.1 (519.4,750.6)	1.3 (-6.2,11.1)
Republic of Uganda	162738 (135098,194245)	897.6 (744,1075.2)	415361 (347108,492485)	923.5 (767.8,1089.2)	2.8 (-4.3,10.6)
Kingdom of Lesotho	13825 (11562,16586)	860.6 (719.8,1024.9)	17023 (14232,20353)	886.5 (737.5,1042.9)	2.3 (-4.8,11.8)
Republic of South Africa	344036 (289552,404948)	909.2 (759.3,1089.9)	524999 (440584,623272)	931.6 (775.9,1106.1)	1.2 (-6.4,9.9)
Republic of Mali	79881 (67185,94928)	871.2 (723.5,1047.1)	226328 (189443,269355)	886 (741.6,1048.2)	-0.3 (-2.1,1.5)
Republic of Guinea-Bissau	9188 (7660,11066)	861.3 (718.9,1024.9)	18975 (15913,22763)	890.7 (742.6,1054.9)	2.3 (-5.5,11.6)
Democratic Republic of Sao Tome and Principe	1143 (950,1363)	851.6 (710.5,1020.3)	2070 (1721,2435)	871 (727.7,1041.3)	1.8 (-4.9,10)
Kingdom of Bahrain	4316 (3591,5102)	867.2 (722.2,1030.1)	13388 (11123,15891)	889 (736.6,1059.5)	3.1 (-6,13.6)
Republic of Chad	55234 (46126,65881)	850.5 (707.3,1023.7)	166040 (138215,198286)	862.5 (721,1036.5)	2.3 (-6.5,13.4)
Republic of Côte d'Ivoire	115096 (96423,136282)	868 (728.3,1030)	265435 (219442,316288)	885 (739.1,1054.3)	0.9 (-7.5,9)
Republic of Botswana	12325 (10301,14677)	870.4 (720.3,1047.3)	22308 (18648,26512)	877.2 (729.4,1038.9)	3.5 (-3.6,12.3)
Republic of Ghana	140039 (116549,167986)	875 (725.2,1047.8)	320038 (267854,380839)	896.1 (747.3,1063.8)	1.4 (-7.8,12.1)
Togolese Republic	34026 (28281,40578)	881.4 (735.9,1054.1)	78022 (65401,93122)	906 (757.3,1077.2)	2.4 (-5.8,10.9)
Islamic Republic of Mauritania	19191 (15817,22813)	883.3 (735.9,1047.4)	41862 (35113,49571)	905 (757.2,1079.1)	1.5 (-6.3,10)
Kingdom of Eswatini	7528 (6301,8964)	892.6 (751,1050.1)	10732 (8942,12795)	911.7 (765.2,1082.3)	1.1 (-7.5,9.7)
Republic of Benin	44949 (37432,53258)	877.6 (739.1,1043.6)	126908 (105326,150724)	905.2 (752.7,1072.2)	3.3 (-5.2,11.5)
Republic of Cameroon	97118 (81027,114905)	873.4 (728.4,1044.6)	298747 (250342,353675)	886.6 (735.8,1057.9)	0.8 (-1.3,3.2)
Republic of Guinea	54608 (45601,65516)	860.4 (716.6,1025.9)	124211 (103674,147569)	876.2 (727.5,1049.3)	1.8 (-4.7,10.3)
Republic of Liberia	22758 (19203,27013)	863.9 (721.4,1037.5)	51735 (43028,61381)	874.9 (729.5,1040.5)	3.4 (-4.9,11.8)
Federal Republic of Nigeria	832153	886.2	2150983	914.3	-1.1 (-9.9,6.8)

	(698504,974704)	(731.3,1052.2)	(1809589,2536050)	(763.1,1085.7)	
Republic of Sierra Leone	38180 (31962,45394)	884.3 (741.1,1039.2)	82630 (68797,98693)	881.9 (743.5,1040.4)	2.1 (-5.3,11.4)
Republic of Nauru	72 (60,86)	879.5 (735.9,1044.9)	77 (64,92)	899.7 (746.6,1068.3)	2.2 (-6.3,10)
Republic of Niue	16 (13,19)	874.7 (732.4,1039)	12 (10,14)	889.3 (739.9,1062.5)	-0.3 (-9.7,7.3)
Republic of Palau	109 (91,130)	895.5 (745.6,1070.3)	130 (108,153)	890.8 (739,1063.6)	0.6 (-7,9.7)
Republic of Senegal	71101 (59508,84368)	701.8 (583.4,835.1)	149225 (124110,176875)	726.7 (601.9,857.4)	2.9 (-4.6,11.7)
Republic of the Gambia	9262 (7725,10977)	901.4 (753.2,1067.8)	22558 (18921,26659)	891.2 (745.9,1060.4)	1.3 (-7.1,9.8)
Republic of South Sudan	55807 (46632,66212)	724.3 (599.4,862.5)	91201 (76433,108723)	737.5 (611.6,879.5)	1.7 (-5.3,8.7)
Republic of San Marino	206 (171,246)	860.9 (726.2,1019.2)	270 (223,322)	894.6 (751.9,1053.8)	1.8 (-6.1,9.6)
Principality of Monaco	247 (206,293)	883.2 (737.8,1048.3)	308 (255,367)	889.5 (742,1051.7)	-0.5 (-11,7.2)
Republic of Sudan	152596 (126295,181965)	715.1 (593.9,853.3)	332468 (276576,395890)	728.1 (603.1,862.5)	-1.4 (-8.7,6.7)

Location	1990 No (95% UI)	1990 ASRs per 100, 000 (95% UI)	2021 No (95% UI)	2021 ASRs per 100, 000 (95% UI)	Percentage change in the ASRs per 100, 000
Australia	3203 (2664,3793)	25.9 (21.6,30.7)	3811 (3173,4560)	26.8 (22.3,32)	3.3 (-6.1,11.9)
New Zealand	737 (615,875)	25.4 (21.2,30.2)	730 (608,877)	24.7 (20.6,29.6)	-3 (-9.7,2.6)
Antigua and Barbuda	9 (8,11)	16 (13.4,18.9)	8 (7,10)	16.4 (13.6,19.4)	2.1 (-6,11.2)
Barbados	33 (28,39)	16.5 (13.8,19.5)	21 (17,25)	16.3 (13.6,19.5)	-1.1 (-8.8,8.4)
Belize	49 (41,58)	16.3 (13.5,19.2)	58 (48,70)	15.8 (13.2,19)	-2.8 (-9.5,3.9)
Bermuda	7 (6,9)	16.9 (14.1,20.1)	4 (3,5)	16.8 (13.9,20.2)	-0.4 (-8.1,7)
Dominican Republic	1768 (1477,2113)	16.4 (13.7,19.6)	1634 (1364,1941)	15.9 (13.3,18.9)	-2.8 (-11.7,4.1)
Grenada	19 (16,23)	16.4 (13.7,19.6)	11 (9,13)	16.3 (13.7,19.3)	-0.5 (-7.9,7)
Jamaica	450 (378,530)	16.3 (13.7,19.2)	256 (215,302)	16.2 (13.6,19)	-0.7 (-8.2,5.9)
Puerto Rico	525 (431,623)	16.7 (13.7,19.8)	152 (126,182)	17.3 (14.3,20.6)	3.5 (-5.1,13.2)
Saint Kitts and Nevis	7 (6,9)	16.2 (13.4,19.4)	4 (4,5)	15.9 (13.4,18.8)	-1.4 (-10,7.3)
Saint Lucia	28 (23,33)	16.3 (13.5,19.4)	13 (11,16)	16.2 (13.5,19.3)	-0.5 (-8.5,6.9)
Saint Vincent and the Grenadines	20 (17,24)	16.2 (13.6,19.2)	10 (8,12)	16 (13.3,18.9)	-1.4 (-8.9,7.2)
United States Virgin Islands	19 (16,22)	16.8 (14.1,20)	6 (5,7)	17.7 (14.7,21.1)	5.4 (-2.4,14)
Georgia	866 (722,1030)	20.9 (17.4,24.8)	457 (384,542)	21.3 (17.9,25.2)	1.9 (-5.6,10.1)
Mongolia	705 (588,830)	20.3 (17,24)	786 (662,938)	21.4 (18.1,25.6)	5.4 (-3,12.7)
Turkmenistan	1233 (1040,1457)	20.5 (17.3,24.2)	1093 (918,1290)	20.7 (17.3,24.4)	0.9 (-6.8,9.1)
Bosnia and Herzegovina	698 (580,824)	21.5 (17.9,25.4)	282 (235,335)	21.1 (17.6,25.1)	-1.9 (-9.8,5.9)
Hungary	1304 (1093,1543)	21.6	926 (772,1097)	21.7	0.6 (-7,8.5)

		(18.1,25.5)		(18.1,25.7)	
Montenegro	101 (85,120)	21.1 (17.7,25.2)	74 (61,86)	21.8 (18.2,25.6)	3.3 (-3.1,11.9)
North Macedonia	361 (303,427)	22.1 (18.6,26.2)	194 (163,231)	21.4 (17.9,25.4)	-3.3 (-11.1,5)
Romania	3027 (2546,3586)	20.8 (17.5,24.7)	1822 (1530,2147)	21.2 (17.8,25)	1.9 (-6.5,10.1)
Central African Republic	1327 (1111,1573)	21.9 (18.3,26)	1979 (1654,2364)	21.8 (18.2,26)	-0.7 (-8.1,6.5)
Democratic Republic of the Congo	18850 (15799,22287)	21.9 (18.4,25.9)	29765 (24994,35402)	21.8 (18.3,25.9)	-0.7 (-7.8,9.8)
Democratic People's Republic of Korea	4341 (3634,5159)	16.8 (14.1,20)	2317 (1930,2759)	16.4 (13.7,19.5)	-2.5 (-10.5,5.8)
Taiwan (Province of China)	3019 (2524,3573)	19.3 (16.1,22.8)	1520 (1254,1803)	20.1 (16.6,23.8)	4 (-4.8,12.1)
Republic of Moldova	804 (671,951)	21.1 (17.6,25)	289 (241,341)	21.3 (17.8,25.1)	0.7 (-5.8,8.8)
Russian Federation	20017 (16880,23541)	21 (17.7,24.7)	13962 (11726,16532)	21.4 (18,25.4)	2 (-1.1,5.3)
Ukraine	6823 (5697,8048)	21.2 (17.7,25)	2858 (2390,3405)	21.6 (18.1,25.8)	2.2 (-4.6,10)
United Republic of Tanzania	12794 (10679,15257)	22.2 (18.5,26.5)	20011 (16786,23821)	22.2 (18.6,26.4)	-0.2 (-7.5,9.4)
Brunei Darussalam	105 (88,123)	31 (26.1,36.4)	95 (81,114)	32.4 (27.3,38.5)	4.4 (-5.4,11.6)
Japan	19442 (16409,22741)	32.5 (27.4,38)	14276 (12078,16816)	34.5 (29.2,40.7)	6.4 (3.8,9.3)
Republic of Korea	10600 (8949,12433)	32.8 (27.7,38.5)	4350 (3636,5124)	33.9 (28.3,39.9)	3.2 (-5.2,11.1)
Canada	4784 (3997,5688)	24.5 (20.5,29.2)	4346 (3626,5120)	24.5 (20.4,28.9)	-0.1 (-8.2,7.8)
Greenland	14 (12,17)	24.2 (20.3,28.5)	9 (7,10)	23.5 (19.8,27.7)	-2.8 (-10.4,5.1)
United States of America	48671 (41141,57117)	24.3 (20.6,28.5)	43614 (36846,51277)	24.6 (20.8,28.9)	1.2 (-1.1,3.3)
Palestine	762 (637,905)	18 (15.1,21.4)	1033 (861,1228)	17.9 (14.9,21.3)	-0.6 (-8.2,7.8)
Syrian Arab Republic	3931 (3271,4710)	17.8 (14.8,21.4)	1689 (1403,2026)	17.9 (14.9,21.5)	0.4 (-7.2,12.3)
United Arab Emirates	417 (347,501)	18 (14.9,21.6)	631 (529,752)	17.2 (14.4,20.5)	-4.2 (-12,3.6)
American Samoa	14 (12,17)	16.8	6 (5,7)	18.9	12.6 (2.7,21.5)

		(14.1,19.9)		(15.7,22.5)	
Cook Islands	3 (3,4)	16.3 (13.7,19.6)	2 (1,2)	16.5 (13.8,19.6)	0.9 (-6.2,10.5)
Guam	31 (26,37)	17 (14.1,20.2)	21 (18,26)	16.9 (14,20.3)	-0.2 (-7.7,7.9)
Northern Mariana Islands	10 (8,11)	16.3 (13.6,19.2)	5 (4,5)	16 (13.3,18.9)	-2 (-7.8,6.7)
Solomon Islands	108 (90,128)	16.4 (13.7,19.5)	157 (132,186)	16.1 (13.5,19)	-2.1 (-9.7,5.2)
Tokelau	0 (0,0)	16 (13.5,19.1)	0 (0,0)	16.5 (13.9,19.6)	2.8 (-5.2,12.4)
Tuvalu	3 (2,3)	17.9 (15,21.3)	2 (2,3)	16.7 (13.9,19.9)	-6.9 (-15,1.9)
Lao People's Democratic Republic	1323 (1106,1570)	15.9 (13.3,18.8)	1357 (1131,1621)	16.1 (13.4,19.2)	1.5 (-5.6,9.2)
Malaysia	3898 (3249,4596)	16.3 (13.6,19.2)	3781 (3148,4504)	16.5 (13.7,19.6)	1 (-6.9,9.1)
Ireland	617 (515,735)	23.6 (19.7,28.1)	673 (562,802)	24.8 (20.7,29.6)	5.3 (-4.1,15.2)
Burkina Faso	4804 (4031,5675)	21.6 (18.1,25.5)	9894 (8343,11610)	21.7 (18.3,25.5)	0.5 (-6.5,8)
Republic of the Marshall Islands	12 (10,14)	16.1 (13.5,19.2)	9 (7,11)	16.3 (13.6,19.4)	-4.1 (-11.8,4.9)
Republic of Armenia	783 (655,927)	21.6 (18,25.5)	359 (299,425)	21.6 (18,25.6)	-4.1 (-5.9,-2.5)
Democratic Socialist Republic of Sri Lanka	2920 (2417,3467)	16.9 (14,20.1)	2465 (2039,2927)	17 (14.1,20.2)	2.3 (-5.7,10.4)
Kingdom of Cambodia	3313 (2780,3959)	16.1 (13.5,19.3)	2865 (2393,3410)	16.7 (14,19.9)	2.1 (-6.6,11.7)
Democratic Republic of Timor-Leste	286 (241,338)	17.2 (14.5,20.3)	316 (264,378)	16.1 (13.5,19.3)	2.5 (-5.4,10.4)
Independent State of Papua New Guinea	1138 (949,1362)	16.1 (13.4,19.3)	2636 (2217,3142)	16.1 (13.5,19.2)	0.5 (-8.7,9.6)
Kyrgyz Republic	1327 (1112,1572)	20.9 (17.5,24.7)	1590 (1341,1896)	21.2 (17.9,25.2)	3.9 (-5,12.7)
Republic of Indonesia	34071 (28517,40210)	15.1 (12.7,17.9)	32184 (27084,38236)	15.2 (12.8,18.1)	-7.7 (-14.1,-0.1)
Independent State of Samoa	43 (36,51)	16.6 (13.8,19.8)	48 (40,58)	16.3 (13.6,19.4)	0.4 (-8.5,9.2)
Socialist Republic of Viet Nam	15161 (12488,18057)	16.3 (13.4,19.4)	12401 (10385,14794)	16.7 (13.9,19.9)	0.4 (-8.6,7.6)
Kingdom of Tonga	26 (21,31)	16.6 (13.7,19.7)	23 (19,28)	16.4 (13.6,19.4)	4.4 (-4.1,13.8)

Republic of Albania	804 (675,951)	20.7 (17.4,24.5)	288 (240,339)	21.5 (17.9,25.4)	2.1 (-5.9,10)
People's Republic of China	159475 (133375,188027)	14.4 (12.1,17)	77486 (64597,91917)	14.6 (12.2,17.3)	2.9 (-5.1,11.1)
Republic of Kazakhstan	3756 (3156,4510)	21.2 (17.8,25.4)	4274 (3594,5048)	21.8 (18.3,25.7)	2.6 (-7.7,13.2)
Republic of Maldives	68 (57,82)	16 (13.3,19.2)	48 (39,57)	16.5 (13.5,19.6)	1.1 (-7.8,9.1)
Republic of Fiji	145 (122,174)	16.1 (13.5,19.2)	129 (107,154)	14.8 (12.3,17.7)	1.3 (-7,10.4)
Republic of the Union of Myanmar	8549 (7121,10153)	16 (13.3,18.9)	8265 (6928,9902)	16.1 (13.5,19.2)	-6.9 (-14.3,1.3)
Republic of Bulgaria	1032 (867,1233)	21.6 (18.1,25.8)	595 (498,707)	21.3 (17.9,25.3)	0.4 (-2.3,3.4)
Republic of Kiribati	21 (18,25)	16.2 (13.6,19)	22 (19,26)	15.8 (13.2,18.8)	0 (-8.7,7.6)
Republic of Vanuatu	48 (40,57)	16.5 (13.7,19.5)	67 (56,79)	15.9 (13.2,18.9)	0 (-7.6,9)
Republic of the Philippines	15758 (13265,18610)	16.2 (13.7,19.2)	16998 (14273,20027)	15.6 (13.1,18.4)	0.7 (-6.7,9.3)
Republic of Austria	903 (754,1062)	20.2 (16.9,23.8)	831 (688,995)	20.3 (16.8,24.3)	-0.5 (-8.6,8.1)
Republic of Tajikistan	2100 (1750,2501)	21 (17.5,25)	2818 (2365,3335)	20.7 (17.4,24.5)	-1.2 (-8.4,5.5)
Republic of Finland	600 (505,716)	19 (16,22.7)	440 (366,525)	18.9 (15.7,22.6)	1.4 (-2.3,5.1)
Republic of Italy	6140 (5157,7242)	22.8 (19.2,26.9)	4330 (3628,5155)	22.3 (18.7,26.6)	-1.4 (-10.1,4.9)
Republic of Croatia	564 (473,665)	21.1 (17.7,24.9)	373 (309,445)	22.1 (18.3,26.3)	-1.9 (-9.7,5.3)
Kingdom of Thailand	8071 (6712,9661)	16.2 (13.5,19.4)	4557 (3780,5454)	17.7 (14.7,21.1)	3.9 (-5.3,14.2)
Federated States of Micronesia	24 (20,29)	16.1 (13.5,19.2)	14 (12,17)	16 (13.4,18.9)	-0.6 (-9,7)
Republic of Uzbekistan	7030 (5877,8339)	20.6 (17.2,24.5)	7888 (6609,9336)	20.6 (17.3,24.4)	1.3 (-6.4,9.3)
Republic of Peru	5061 (4253,5984)	16.4 (13.8,19.4)	5212 (4362,6172)	16.1 (13.5,19.1)	1 (-7.4,9.7)
People's Democratic Republic of Algeria	6673 (5575,7970)	18 (15.1,21.5)	7804 (6543,9290)	17.9 (15,21.3)	-0.8 (-7.7,6.6)
Kingdom of Morocco	6897 (5731,8213)	18.2 (15.1,21.7)	5492 (4611,6504)	17.9 (15.1,21.2)	4.1 (-5.1,12.8)

Federal Democratic Republic of Nepal	5886 (4938,7002)	15.6 (13.1,18.6)	4736 (3927,5655)	15.4 (12.8,18.4)	9.2 (-0.7,18.6)
Republic of Azerbaijan	1889 (1582,2248)	21.2 (17.8,25.3)	1395 (1164,1651)	21.7 (18.1,25.6)	-5.9 (-13.6,2.3)
Republic of Poland	5698 (4804,6740)	21.7 (18.3,25.7)	3595 (3019,4253)	21.9 (18.4,25.9)	-3 (-10.7,4)
Republic of Serbia	1478 (1236,1731)	22.4 (18.7,26.2)	644 (537,769)	20 (16.6,23.8)	-8.5 (-15.8,-1)
Republic of Estonia	217 (183,258)	21 (17.7,25)	138 (116,164)	22 (18.6,26.2)	2 (-7.1,12)
Principality of Andorra	6 (5,7)	23.9 (20,28.6)	5 (4,6)	24.1 (20.2,28.7)	-4.2 (-10.5,2.8)
Republic of Slovenia	234 (197,276)	21.9 (18.4,25.8)	203 (170,243)	22.7 (19,27.2)	0.1 (-6.4,9)
Kingdom of Belgium	1213 (1015,1446)	20.1 (16.8,24)	1092 (910,1295)	19.8 (16.5,23.5)	-1 (-9,6.8)
Czech Republic	1356 (1130,1602)	21.9 (18.3,25.9)	1132 (944,1340)	22.1 (18.5,26.2)	-2.6 (-10.5,5.4)
Republic of Singapore	845 (715,999)	34.9 (29.5,41.2)	885 (749,1051)	33.5 (28.3,39.7)	0.8 (-2.3,3.3)
Kingdom of Denmark	650 (553,766)	21.2 (18.1,25)	648 (547,768)	21.1 (17.8,25.1)	6.4 (4.1,8.6)
Republic of Lithuania	578 (480,685)	21.3 (17.7,25.3)	245 (204,290)	21.4 (17.9,25.3)	0.7 (-7.6,8.5)
Republic of Belarus	1432 (1196,1696)	21 (17.5,24.8)	857 (716,1022)	21.5 (17.9,25.6)	-0.7 (-9,8.1)
French Republic	7371 (6187,8799)	20 (16.8,23.9)	6673 (5613,7909)	20.1 (16.9,23.8)	2.3 (-5.8,10.1)
Republic of Malta	53 (45,63)	19.9 (16.7,23.6)	42 (35,50)	20.1 (16.9,24)	0.5 (-8.2,8.2)
Federal Republic of Germany	8403 (7070,10042)	19.8 (16.7,23.7)	7656 (6336,9125)	20.3 (16.8,24.1)	0.5 (-8.6,9.8)
Republic of Iceland	52 (44,62)	23.3 (19.7,27.6)	53 (44,62)	23.7 (19.9,28)	1 (-7.2,10.7)
Republic of Chad	3359 (2847,3976)	21.9 (18.5,25.9)	8943 (7465,10658)	21.6 (18,25.7)	1 (-6.7,9.2)
State of Israel	917 (769,1092)	18.1 (15.2,21.6)	1593 (1336,1897)	18 (15.1,21.4)	4.9 (-3.8,13.8)
Slovak Republic	822 (689,969)	21.6 (18.1,25.4)	589 (495,701)	22 (18.5,26.2)	-1.6 (-8.9,6.7)
Republic of Latvia	373 (312,442)	21.1 (17.6,25)	175 (145,207)	21.6 (17.9,25.6)	2 (-8.4,10.1)

Kingdom of Norway	523 (441,618)	18.3 (15.4,21.6)	490 (412,584)	18.3 (15.4,21.8)	-1.6 (-8.5,6.9)
Republic of the Gambia	485 (406,576)	22.4 (18.7,26.6)	815 (690,961)	22 (18.6,25.9)	-2.1 (-9.7,4.5)
Republic of Guinea	3016 (2524,3621)	21.6 (18.1,26)	5167 (4340,6112)	21.7 (18.2,25.6)	-3.7 (-11.4,3.6)
Argentine Republic	7922 (6622,9400)	23.7 (19.8,28.2)	6258 (5230,7384)	24.3 (20.3,28.7)	17.1 (6.7,26.4)
Republic of Cyprus	142 (120,170)	21.6 (18.2,25.7)	146 (121,174)	20.1 (16.7,24)	0 (-7.6,8.6)
Kingdom of the Netherlands	1938 (1605,2299)	20.4 (16.9,24.2)	1732 (1431,2064)	20.2 (16.7,24.1)	0.2 (-7.2,8.6)
Hellenic Republic	997 (838,1178)	19.8 (16.6,23.4)	796 (665,955)	20 (16.7,24)	-10.7 (-18.9,-2.8)
Kingdom of Spain	4337 (3648,5186)	22.6 (19,27)	3757 (3169,4414)	23.3 (19.7,27.4)	-2.3 (-9.3,6)
Arab Republic of Egypt	16318 (13604,19225)	18.1 (15.1,21.3)	22189 (18534,26179)	17.9 (15,21.2)	-1.5 (-8.5,7.8)
Eastern Republic of Uruguay	649 (544,771)	24.2 (20.3,28.7)	416 (345,497)	24.3 (20.2,29.1)	-1.1 (-8.6,6.5)
Swiss Confederation	852 (708,1013)	21 (17.5,25)	868 (721,1033)	20.5 (17.1,24.4)	-0.9 (-9,7.6)
United Kingdom of Great Britain and Northern Ireland	6080 (5084,7186)	15.9 (13.3,18.8)	5654 (4743,6679)	16.9 (14.2,20)	0.4 (-3.2,4.4)
Grand Duchy of Luxembourg	49 (41,59)	20.3 (17,24.2)	64 (54,77)	19.8 (16.6,23.7)	-2.7 (-5.1,-0.3)
Plurinational State of Bolivia	1745 (1469,2074)	15.9 (13.4,19)	1868 (1568,2236)	16 (13.4,19.1)	0.7 (-6.8,9.4)
Republic of Chile	3509 (2938,4110)	23.9 (20,28)	2336 (1952,2760)	24 (20,28.3)	2.8 (-4,8.8)
Republic of Suriname	74 (62,88)	16.9 (14.1,20.1)	69 (58,82)	16.3 (13.6,19.2)	-1.9 (-9.7,6.6)
Republic of Colombia	7914 (6663,9439)	17.9 (15.1,21.3)	5805 (4837,6926)	17.9 (14.9,21.4)	-0.7 (-6.9,6.5)
Commonwealth of the Bahamas	42 (35,51)	16 (13.2,19.1)	30 (25,36)	15.7 (13,18.6)	-0.5 (-9.1,8.6)
Republic of Guyana	212 (175,253)	16.9 (14,20.2)	118 (99,140)	16.4 (13.8,19.5)	-0.8 (-9.6,7.5)
Commonwealth of Dominica	15 (12,18)	16.7 (13.9,19.9)	5 (4,6)	16 (13.4,19.1)	0.6 (-8.4,8.2)
Republic of Trinidad and Tobago	186 (155,223)	16.3 (13.5,19.5)	115 (97,136)	16.1 (13.6,19.1)	-2.2 (-10.5,6.1)
Republic of Ecuador	2306 (1935,2742)	15.9 (13.4,19)	2469 (2044,2936)	16 (13.3,19.1)	-0.6 (-9.4,8)

Republic of Costa Rica	704 (584,843)	18 (14.9,21.6)	477 (396,570)	18.2 (15.1,21.7)	-2.5 (-9.9,4.4)
Republic of Honduras	1527 (1276,1822)	17.8 (14.9,21.2)	1855 (1558,2204)	17.4 (14.6,20.6)	-4.4 (-12.1,2)
Portuguese Republic	1115 (937,1333)	20.1 (16.9,24.1)	767 (640,914)	19.4 (16.2,23.2)	0 (-8.2,9.2)
Republic of Cuba	1409 (1171,1663)	16.4 (13.7,19.4)	791 (661,937)	16.4 (13.7,19.5)	-0.7 (-9.4,8.5)
Republic of Guatemala	2872 (2389,3409)	17.3 (14.4,20.5)	2884 (2402,3424)	20.2 (16.9,24)	-3.8 (-11.1,3.3)
United Mexican States	21966 (18437,26029)	18 (15.1,21.4)	15894 (13410,18794)	17.5 (14.8,20.7)	0.2 (-7,8.2)
Bolivarian Republic of Venezuela	4696 (3904,5597)	17.8 (14.8,21.2)	3532 (2961,4185)	16.3 (13.6,19.3)	1.3 (-7.1,10.3)
Republic of Paraguay	908 (750,1074)	14.6 (12.1,17.3)	902 (750,1071)	14.6 (12.1,17.3)	-5.4 (-12.7,1)
Republic of Turkey	13087 (10950,15492)	18.4 (15.4,21.8)	9119 (7601,10797)	18.8 (15.7,22.3)	-0.4 (-1.8,1.3)
Republic of Haiti	1945 (1622,2319)	16.1 (13.4,19.1)	2588 (2158,3063)	15.8 (13.2,18.7)	-0.7 (-8.7,7.1)
Kingdom of Bahrain	117 (98,138)	18.3 (15.4,21.7)	147 (122,176)	17.4 (14.4,20.7)	1.5 (-6.6,9.8)
Republic of India	194181 (163238,229934)	16.1 (13.6,19.1)	182559 (153502,215016)	17.4 (14.6,20.5)	-2 (-8.9,5.6)
State of Kuwait	303 (251,360)	18.8 (15.6,22.3)	450 (372,534)	18.8 (15.5,22.3)	-1.5 (-8.8,7.1)
Lebanese Republic	830 (697,985)	20.3 (17,24.1)	775 (643,919)	20.4 (16.9,24.2)	-1.7 (-8.4,6.4)
Republic of El Salvador	1501 (1254,1783)	18 (15,21.3)	991 (833,1185)	17.7 (14.9,21.2)	-0.4 (-7,7.2)
Republic of Nicaragua	1208 (1014,1444)	17.7 (14.8,21.1)	1080 (898,1281)	17.6 (14.6,20.8)	1.2 (-5.1,9.3)
Republic of Panama	502 (420,595)	17.7 (14.8,21)	602 (505,710)	17.7 (14.9,20.9)	-0.7 (-7.9,8.1)
Federative Republic of Brazil	22451 (18722,26553)	14.5 (12.1,17.1)	23242 (19457,27531)	14.6 (12.2,17.3)	-0.9 (-8.2,7.8)
Islamic Republic of Iran	13360 (11237,15776)	18 (15.1,21.2)	8971 (7510,10557)	18.1 (15.2,21.3)	-1.5 (-10.2,7)
Republic of Iraq	5933 (4916,7046)	17.9 (14.8,21.2)	7341 (6175,8783)	18.4 (15.5,22.1)	0.5 (-6.7,8.9)
State of Libya	1146 (962,1360)	18.3	675 (560,799)	17.5	0.2 (-3.1,3.6)

		(15.4,21.7)		(14.6,20.8)	
State of Qatar	109 (91,130)	20 (16.7,23.7)	362 (300,429)	19.5 (16.2,23.1)	0.3 (-8.5,8.9)
Kingdom of Saudi Arabia	4438 (3706,5233)	18.1 (15.1,21.3)	3995 (3318,4730)	17.9 (14.9,21.2)	-1.8 (-9.8,5.9)
Republic of Angola	5250 (4407,6212)	21.8 (18.3,25.8)	12522 (10540,14721)	21.6 (18.2,25.4)	-1.6 (-9.1,6.9)
Republic of the Congo	960 (801,1142)	21.9 (18.3,26.1)	1342 (1123,1575)	22 (18.4,25.8)	0.3 (-7.4,8.9)
Republic of Burundi	2707 (2269,3211)	21.7 (18.2,25.7)	4887 (4110,5742)	21.8 (18.3,25.6)	0.8 (-2.6,4.1)
Republic of Djibouti	146 (122,173)	19.5 (16.4,23.2)	267 (222,315)	18.4 (15.4,21.8)	-2.3 (-8.9,7.2)
Kingdom of Sweden	1333 (1098,1617)	22.3 (18.4,27.1)	1261 (1046,1502)	23 (19.1,27.4)	-1.5 (-6,3.1)
Sultanate of Oman	636 (530,759)	18.4 (15.4,22)	709 (587,848)	18.7 (15.4,22.3)	-0.6 (-9.4,7)
Republic of Tunisia	1913 (1595,2290)	18.1 (15.1,21.7)	1447 (1201,1718)	18 (15,21.4)	-0.9 (-9,7.1)
State of Eritrea	1551 (1299,1838)	21.8 (18.3,25.9)	2054 (1720,2424)	21.9 (18.4,25.9)	1.6 (-6.7,10.3)
Republic of Kenya	10497 (8841,12377)	22.2 (18.7,26.2)	12554 (10570,14775)	22.2 (18.7,26.1)	3.2 (-5.5,10.9)
Hashemite Kingdom of Jordan	1170 (976,1388)	18.3 (15.3,21.7)	1849 (1539,2194)	17.8 (14.9,21.2)	-2.2 (-5.4,1.3)
Republic of Yemen	5605 (4660,6697)	17.8 (14.8,21.2)	8276 (6955,9855)	17.8 (14.9,21.2)	0.6 (-7.6,9.6)
Islamic Republic of Afghanistan	3811 (3195,4530)	18 (15.1,21.4)	10067 (8350,12027)	17.3 (14.4,20.7)	-1.7 (-9.2,6.4)
People's Republic of Bangladesh	28367 (23658,33609)	13.9 (11.6,16.5)	19128 (15906,22673)	14.3 (11.9,16.9)	0.7 (-8.1,10)
Kingdom of Bhutan	165 (138,195)	15.5 (12.9,18.3)	93 (77,111)	15.5 (12.9,18.5)	-0.7 (-9.3,6.1)
Islamic Republic of Pakistan	31286 (26369,36853)	15.5 (13.1,18.3)	45664 (38148,53530)	15.3 (12.8,17.9)	-1.3 (-9.1,5.7)
Republic of Equatorial Guinea	213 (178,252)	21.6 (18.1,25.6)	394 (330,464)	21.9 (18.4,25.8)	-1.7 (-9,7.2)
Republic of Malawi	5328 (4455,6345)	22.2 (18.6,26.5)	6021 (5078,7097)	22.1 (18.6,26)	-0.3 (-7.9,7.7)
Union of the Comoros	214 (179,251)	22.6 (18.9,26.5)	183 (153,215)	22.4 (18.8,26.4)	0.2 (-8.1,8.1)

Republic of Madagascar	5544 (4644,6600)	22.2 (18.6,26.4)	9203 (7666,10889)	22.7 (18.9,26.9)	-0.2 (-7.2,7.6)
Gabonese Republic	376 (315,442)	22 (18.4,25.9)	457 (384,539)	22.1 (18.6,26.1)	0.3 (-8.1,8.9)
Federal Democratic Republic of Ethiopia	25648 (21645,30046)	22 (18.5,25.7)	36863 (30933,43703)	22.1 (18.5,26.2)	-0.9 (-2.5,0.6)
Republic of Mozambique	6532 (5464,7757)	22.3 (18.7,26.5)	11551 (9720,13657)	21.8 (18.3,25.8)	-1 (-8.4,8.1)
Republic of Seychelles	13 (11,16)	16.6 (13.8,19.8)	13 (10,15)	16.6 (13.8,19.7)	-0.5 (-8.5,6.9)
Federal Republic of Somalia	4125 (3453,4878)	22.2 (18.6,26.3)	10045 (8407,11745)	21.8 (18.2,25.5)	2.1 (-6.6,10.9)
Republic of Rwanda	3332 (2780,3928)	21.8 (18.2,25.7)	3926 (3291,4640)	22.2 (18.6,26.2)	0.6 (-6.2,9.4)
Republic of Uganda	9827 (8153,11697)	22.2 (18.5,26.5)	16868 (14279,19938)	22.3 (18.9,26.4)	-1 (-7.7,5.9)
Republic of Mauritius	181 (152,216)	16.3 (13.6,19.4)	101 (83,120)	16.5 (13.6,19.6)	-1.3 (-8.4,6.2)
Republic of Zambia	4084 (3387,4876)	22.3 (18.5,26.6)	6353 (5304,7528)	21.9 (18.3,26)	0.8 (-5.7,9.2)
Kingdom of Lesotho	568 (476,676)	22.1 (18.6,26.3)	441 (371,525)	21.8 (18.3,25.9)	0.9 (-3.1,4.5)
Republic of Benin	2465 (2064,2909)	21.6 (18.1,25.5)	5459 (4561,6451)	21.8 (18.2,25.7)	-2.5 (-10.6,6.2)
Republic of Zimbabwe	4136 (3459,4937)	22.2 (18.6,26.6)	4879 (4106,5762)	21.8 (18.3,25.7)	-3.5 (-11.6,4.7)
Kingdom of Eswatini	340 (286,403)	22 (18.4,26)	306 (255,363)	22.1 (18.4,26.2)	-1.8 (-9.1,6.5)
Islamic Republic of Mauritania	919 (763,1098)	22.1 (18.4,26.5)	1424 (1196,1676)	21.9 (18.4,25.8)	2.2 (-7.1,11.4)
Republic of Botswana	490 (411,582)	22 (18.5,26.1)	510 (428,600)	21.9 (18.4,25.8)	7.9 (5.6,10.6)
Republic of Ghana	6330 (5309,7512)	21.9 (18.3,25.9)	10125 (8497,11971)	21.8 (18.3,25.8)	-19.8 (-27.4,-13.6)
Republic of Guinea-Bissau	476 (399,567)	21.6 (18.1,25.7)	745 (626,888)	21.7 (18.2,25.8)	2.5 (-7.2,11.4)
Republic of Liberia	1257 (1062,1495)	23.3 (19.7,27.7)	1714 (1432,2022)	22 (18.4,25.9)	-5.2 (-13.8,2.9)
Republic of Côte d'Ivoire	5767 (4804,6808)	21.7 (18.1,25.6)	9936 (8307,11736)	21.9 (18.3,25.9)	-0.1 (-8.5,8.7)
Republic of Namibia	535 (448,633)	21.7	608 (512,725)	22.1	1.2 (-6.6,11.3)

		(18.2,25.8)		(18.6,26.3)	
Republic of South Africa	11050 (9281,13012)	22 (18.4,25.9)	10403 (8757,12267)	22 (18.5,26)	-0.3 (-7.8,7.6)
Republic of Cameroon	4983 (4190,5867)	21.8 (18.3,25.6)	10759 (9013,12705)	21.6 (18.1,25.5)	0.5 (-7,7.5)
Republic of Cabo Verde	133 (112,158)	22.1 (18.6,26.2)	90 (75,107)	22.2 (18.5,26.4)	-3.3 (-11.1,5.6)
Republic of Mali	4734 (3970,5615)	22.1 (18.5,26.2)	11077 (9346,13156)	21.7 (18.3,25.8)	0.2 (-7.5,8.5)
Republic of the Niger	4638 (3908,5569)	21.7 (18.3,26)	12208 (10190,14315)	21.5 (18,25.3)	-5.6 (-13.2,2.6)
Federal Republic of Nigeria	43516 (36667,51141)	22.2 (18.7,26.1)	87122 (73209,102893)	22 (18.5,26)	0.4 (-7.5,8.8)
Democratic Republic of Sao Tome and Principe	49 (41,58)	22.1 (18.4,26.2)	52 (43,61)	22 (18.3,25.9)	0.9 (-6,9.9)
Republic of Senegal	3636 (3041,4285)	21.9 (18.3,25.9)	5007 (4174,5912)	21.7 (18.1,25.6)	-0.5 (-7.8,7.8)
Principality of Monaco	3 (3,4)	23.7 (19.9,28)	3 (2,3)	19 (15.9,22.6)	2.4 (-6.9,13.4)
Togolese Republic	1664 (1385,1985)	21.6 (18,25.8)	2537 (2132,2990)	21.8 (18.3,25.7)	0.6 (-6.7,8.2)
Republic of Sierra Leone	2141 (1800,2526)	22 (18.5,25.9)	3154 (2641,3759)	21.8 (18.2,26)	-0.8 (-8.3,8.8)
Republic of South Sudan	2722 (2302,3213)	22.2 (18.8,26.2)	4043 (3393,4787)	22.1 (18.5,26.1)	0.5 (-3.8,5.4)
Republic of San Marino	2 (2,3)	19.2 (16,23)	2 (2,3)	21.3 (17.8,25.6)	1.8 (-5.2,10.1)
Republic of Nauru	3 (2,3)	16.4 (13.7,19.5)	2 (2,3)	16.2 (13.5,19.2)	0.6 (-7.8,8.6)
Republic of Sudan	7776 (6456,9243)	18.5 (15.4,22)	9817 (8189,11639)	17.8 (14.9,21.1)	11 (-0.8,20.2)
Republic of Niue	0 (0,0)	15.9 (13.3,18.9)	0 (0,0)	16 (13.5,18.9)	3 (-8.3,14.8)
Republic of Palau	2 (2,3)	16.1 (13.4,19.1)	1 (1,2)	16.4 (13.7,19.4)	-3.9 (-11.2,3.4)

Location	1990 No (95% UI)	1990 ASRs per 100, 000 (95% UI)	2021 No (95% UI)	2021 ASRs per 100, 000 (95% UI)	Percentage change in the ASRs per 100, 000
Australia	36204 (24960,50585)	217.6 (150.1,303.9)	55151 (38177,77960)	223.5 (155,315)	2.7 (-7.3,11.2)
New Zealand	7159 (4995,10036)	212 (148.1,297.7)	10855 (7492,15250)	218.3 (150.3,306.7)	2.9 (-5.1,9.7)
Antigua and Barbuda	80 (55,112)	132.2 (89.7,184.9)	117 (80,163)	133.2 (91.2,187.1)	0.8 (-8,10.4)
Barbados	332 (226,473)	132.9 (90.8,188.9)	376 (254,528)	133.6 (90.2,188.7)	0.5 (-8.2,11)
Belize	252 (175,352)	128.6 (89.1,179.2)	561 (377,783)	127.8 (86.1,179)	-0.6 (-8.9,7.1)
Bermuda	81 (56,112)	136.9 (95.2,190.8)	82 (56,115)	139.2 (94.6,196.9)	1.6 (-6.7,9.9)
Dominican Republic	9365 (6365,13104)	125.1 (85.4,175.4)	14058 (9607,19552)	126.9 (86.8,176.6)	1.5 (-8.6,10.7)
Grenada	114 (78,160)	128.2 (87.4,178.5)	133 (92,185)	130.5 (90.1,182.8)	1.8 (-7.2,10.4)
Jamaica	3186 (2161,4472)	131.6 (89.3,183.9)	3639 (2552,5065)	131.8 (92.2,182.6)	0.2 (-8.2,8.5)
Puerto Rico	4887 (3362,6930)	135.3 (93.1,191.7)	4125 (2833,5707)	136.9 (93.4,190.8)	1.2 (-8.8,11.7)
Saint Kitts and Nevis	54 (37,75)	127.9 (88.1,178)	75 (52,105)	130.1 (89.4,182.3)	1.7 (-8.2,11.9)
Saint Lucia	183 (123,257)	129 (87.3,181.6)	225 (153,315)	131.2 (88.7,184.5)	1.7 (-8.5,10.5)
Saint Vincent and the Grenadines	146 (99,204)	128.7 (86.5,179.3)	145 (99,203)	129.7 (88.6,182.4)	0.7 (-7.6,10.5)
United States Virgin Islands	142 (95,202)	131.9 (88.7,187.3)	104 (71,145)	132.9 (91.1,185.5)	0.7 (-7.3,10.1)
Georgia	9282 (6283,12973)	169.9 (114.8,237.7)	5821 (4080,8209)	170.9 (119.4,242)	0.6 (-7.2,8.9)
Mongolia	3625 (2489,5078)	158.9 (108.6,222)	5523 (3840,7824)	161.2 (112,227.8)	1.5 (-6.9,9.4)
Turkmenistan	6330 (4395,8992)	162.5 (112.1,229.5)	8750 (5872,12284)	166.5 (111.7,233.7)	2.4 (-6.5,11.4)
Bosnia and Herzegovina	8060 (5525,11485)	178 (121.8,253.2)	5632 (3931,7947)	182.3 (127.5,258.5)	2.4 (-6.3,11.3)
Hungary	17661 (12196,24839)	176.3 (121.3,248.9)	16201 (11131,22380)	182.5 (124.4,253.3)	3.5 (-4.8,12.6)

Montenegro	1134 (779,1615)	181.2 (124.7,258.1)	1087 (760,1537)	183.5 (127.7,260.1)	1.3 (-6.2,9.8)
North Macedonia	3510 (2375,4932)	175.4 (118.6,246.4)	3758 (2555,5260)	179.6 (123.1,251.7)	2.4 (-7,12.1)
Romania	39231 (26644,55431)	170.5 (115.9,241.1)	31067 (21342,43185)	176.5 (121.7,244.6)	3.5 (-4.2,12.5)
Central African Republic	4613 (3183,6516)	156 (108.4,218.5)	9291 (6298,13238)	157.4 (106.8,223.7)	0.9 (-8.6,8.8)
Democratic Republic of the Congo	65705 (45693,92602)	159.3 (110.2,224.6)	158607 (107451,223871)	165.1 (112.3,232.4)	3.6 (-4.4,14.2)
Democratic People's Republic of Korea	27284 (18771,38170)	129.8 (89.3,181.9)	34596 (24083,48528)	133.9 (93.5,187.1)	3.2 (-5,11.9)
Taiwan (Province of China)	33911 (23794,48007)	164.2 (115.2,231.9)	38754 (26701,54538)	172.2 (119.4,241.7)	4.9 (-3.1,13.3)
Republic of Moldova	7487 (5081,10481)	167.9 (114.1,235.3)	5836 (3954,8252)	172.5 (116.8,244.3)	2.7 (-5.7,10.7)
Russian Federation	249540 (170445,349118)	168.6 (114.9,235.9)	237330 (163785,332074)	173.9 (119.7,244)	3.1 (-0.2,6.5)
Ukraine	87026 (59098,122686)	170.9 (115.9,240.4)	68852 (47411,95561)	172.7 (119.1,240.3)	1 (-6.5,10.2)
United Republic of Tanzania	45026 (31290,62592)	162.7 (113.9,225.1)	103772 (71114,146584)	167.6 (115.1,236.8)	3 (-5.5,12.7)
Brunei Darussalam	695 (488,969)	255.6 (178.5,358.1)	1178 (806,1646)	257.7 (176.3,361.1)	0.8 (-8.8,9.1)
Japan	336208 (234691,465749)	274.9 (191.7,381.4)	340640 (236444,476164)	299.1 (207.2,420)	8.8 (6.2,11.8)
Republic of Korea	118809 (81838,165611)	261.8 (180.5,365)	138184 (95735,191645)	283.8 (196,395.6)	8.4 (-0.8,16.9)
Canada	55374 (38372,76807)	206.8 (143.1,286.5)	73932 (51632,104753)	209.6 (146.9,296)	1.3 (-6.9,11.1)
Greenland	108 (75,154)	188.7 (131.2,268.6)	105 (73,148)	190.4 (131.3,267.9)	0.9 (-7.9,8.6)
United States of America	495965 (341955,691923)	199.8 (137.8,278.9)	639136 (443188,891390)	203.3 (141,283.7)	1.8 (-0.8,4.1)
Palestine	3126 (2133,4413)	143.4 (98.3,201.4)	7797 (5338,10934)	145.9 (99.9,204.5)	1.7 (-7.3,12.1)
Syrian Arab Republic	19203 (13129,26783)	142.7 (98.2,198.6)	19995 (13733,28294)	141.1 (96.7,200.1)	-1.1 (-9.2,10.9)
United Arab Emirates	3097 (2113,4329)	156.2 (106.7,217.7)	15980 (10922,22626)	157.2 (107.8,220.9)	0.6 (-8.1,10.6)
American Samoa	68 (47,97)	133.7	66 (46,95)	132.7	-0.8 (-10.1,7.1)

		(92.2,190.3)		(91.1,189.4)	
Cook Islands	27 (18,38)	137 (93.2,194.7)	23 (16,33)	135.9 (92.4,189.7)	-0.8 (-9.5,9.2)
Guam	196 (133,274)	139 (94,194.3)	213 (143,300)	136.8 (92.2,192.8)	-1.6 (-9.7,6.5)
Northern Mariana Islands	64 (44,91)	138.1 (94.9,193.9)	67 (46,93)	138.1 (94.9,193)	0 (-6.9,9.7)
Solomon Islands	456 (307,638)	126.8 (84.7,177.2)	903 (618,1270)	126.1 (86.2,176.8)	-0.5 (-9.4,8.1)
Tokelau	2 (1,3)	131.5 (90.3,186.1)	2 (1,3)	134.1 (92.2,189.3)	2 (-6.2,11.3)
Tuvalu	12 (8,17)	122.9 (84.6,171.2)	16 (11,23)	129 (87.1,180.9)	5 (-4.4,15.1)
Lao People's Democratic Republic	5343 (3722,7540)	120.6 (84.4,170)	9593 (6535,13525)	126.1 (85.9,177.6)	4.6 (-4.1,12.9)
Malaysia	24570 (16656,34695)	133.7 (90.5,189.3)	44217 (30560,62353)	138.2 (95.5,194.9)	3.4 (-6.1,13.5)
Ireland	6924 (4824,9672)	194.1 (135.2,271.3)	9519 (6520,13219)	200.9 (138.5,279.3)	3.5 (-6.3,13.9)
Burkina Faso	16387 (11349,23116)	159.8 (110.3,224.7)	39778 (27898,55873)	163.7 (114.9,228.4)	2.4 (-5.9,11.5)
Plurinational State of Bolivia	8185 (5523,11500)	121 (81.9,168.8)	411 (280,587)	126 (85.9,179)	0.9 (-8.8,9.7)
Bolivarian Republic of Venezuela	27679 (19114,38459)	140.6 (97.2,194.8)	86247 (59117,122373)	136 (93,192.7)	-0.1 (-8.4,8.9)
Principality of Andorra	93 (64,131)	171.7 (118.3,243.1)	9308 (6379,13011)	181.4 (123.9,254)	0.1 (-7.8,9.8)
Republic of Italy	99228 (67921,138375)	183.8 (125.6,256.6)	19176 (13069,26861)	151.7 (103.3,212.3)	10.4 (0.8,23)
Commonwealth of Dominica	96 (66,135)	129.6 (88.5,181.3)	15683 (10695,22195)	116.5 (79.5,164.7)	3.3 (-6.6,12.5)
Republic of Chile	26616 (18531,37107)	196.1 (136.6,273.6)	3587 (2472,5031)	161.4 (112.1,226)	0.1 (-8.2,8.7)
Republic of Ecuador	13090 (9061,18572)	125.3 (86.7,178)	7748 (5354,10925)	151.9 (104.7,214.4)	0 (-8.3,9.9)
State of Kuwait	2816 (1945,3993)	157.9 (109.1,223.3)	9669 (6538,13341)	177.7 (119.7,245)	2.4 (-6.4,10.4)
Lebanese Republic	4856 (3380,6774)	157.9 (110.2,220.3)	57738 (39908,80383)	163.8 (114.2,227.4)	1.8 (-6.8,11.2)
Republic of Malawi	17058 (11800,23896)	161.5 (112,226)	89582 (61198,125927)	169.7 (116.2,238.2)	1.4 (-7.2,10.3)
Republic of the Union of Myanmar	51317 (35234,72162)	121.4	4160 (2926,5843)	168.4	-0.4 (-8.8,8.5)

		(83.2,170.6)		(118.5,236.3)	
Republic of the Congo	4165 (2850,5884)	161.4 (111.1,226.6)	7672 (5137,10759)	156.1 (104.9,218.7)	-0.5 (-8.7,8.5)
Republic of Tajikistan	9178 (6341,13065)	162.4 (111.6,230.3)	15568 (10764,22121)	166 (115.1,235.1)	1.7 (-7,11.5)
Republic of Sierra Leone	7143 (4868,9975)	162.3 (111.2,226.2)	50068 (35040,70057)	169.9 (118.7,236.8)	0.6 (-7.6,8.5)
Republic of Namibia	2468 (1712,3435)	165 (114.9,229.1)	64669 (44726,89944)	180.9 (125.2,251.8)	2.3 (-5,11.9)
Independent State of Samoa	233 (160,325)	130.8 (89.7,182.8)	145 (99,204)	138.2 (94.3,194.6)	2.9 (-6.2,11.7)
Democratic Socialist Republic of Sri Lanka	24102 (16701,33763)	136.8 (94.7,191.2)	931 (651,1301)	121.3 (84.9,169.6)	0.2 (-9.7,9.7)
Eastern Republic of Uruguay	6041 (4097,8308)	196.2 (133.1,270.2)	62565 (42927,87689)	137.5 (94.4,193.5)	1.2 (-7.6,11.5)
Republic of the Philippines	84268 (56895,118949)	127.2 (86.1,179.7)	1842 (1293,2602)	126.9 (89.2,179.6)	2.7 (-1.4,6.7)
Republic of Poland	65857 (45233,91745)	174.9 (120.2,243.6)	42570 (29089,59967)	164.8 (113.2,232.4)	3.1 (-5.6,13.2)
Grand Duchy of Luxembourg	609 (415,842)	166.5 (114,231.3)	4525 (3081,6351)	177.5 (121.7,249.6)	2.3 (-7.5,11.2)
Republic of Austria	12200 (8282,16943)	165.1 (112,229.3)	6936 (4738,9759)	148.6 (101.5,208.9)	0.7 (-7.7,10.8)
Republic of Madagascar	20878 (14562,29162)	164.1 (114.2,228.7)	247435 (166839,346062)	114.3 (77,160.1)	3.7 (-5.3,13.2)
Republic of San Marino	38 (27,54)	168.4 (116.2,235.2)	71764 (48698,101559)	125.8 (85.4,178)	0.3 (-7.3,9.8)
Republic of Yemen	19751 (13801,28035)	134.4 (93.8,190.8)	47312 (32150,66419)	130.1 (88.4,182.7)	1 (-2.4,4)
Republic of the Gambia	1746 (1170,2444)	166.8 (112.6,233.7)	101268 (69594,141723)	165.2 (112.7,232.3)	1.6 (-7.6,12.3)
Kingdom of Tonga	135 (93,189)	130.6 (90.1,182.9)	15544 (10639,21490)	177.3 (121.2,247.7)	0.3 (-7.4,8.4)
Kingdom of Lesotho	2608 (1784,3641)	159.3 (108.7,223.1)	80710 (56601,110405)	192.8 (134.5,265.4)	3.5 (-4.8,13.6)
Kingdom of Belgium	15721 (10731,21815)	165.3 (112.9,230.1)	7801 (5346,10900)	155.5 (106.7,218.3)	10.5 (0.4,21)
People's Republic of China	1392966 (943870,1971071)	115.8 (78.6,163.7)	17174 (11706,24280)	162.9 (111.2,230.1)	2.9 (-0.1,6.4)
Republic of Serbia	16648 (11479,23413)	174.9 (120.6,246.1)	4988 (3417,6919)	173.1 (118.2,240.4)	5.1 (-4.5,15.2)

Federative Republic of Brazil	174993 (121003,245475)	113.2 (78.2,158.6)	15431 (10485,21798)	161.5 (109.7,227.9)	4.1 (-4.9,14.7)
Republic of Malta	608 (421,852)	166.7 (115.3,233.3)	2503 (1745,3484)	158.7 (110.5,221.9)	2.6 (-6.3,12.1)
Republic of Uzbekistan	35925 (24469,50449)	163.6 (111.7,230.4)	14263 (9709,20193)	136.2 (92.7,192.6)	2.1 (-1.7,5.7)
Kingdom of Morocco	36990 (24926,51673)	139.6 (94,195.9)	37255 (25067,52394)	116.9 (78.7,164.4)	2.6 (-5.2,11.7)
Republic of Ghana	26328 (18381,36764)	164.8 (114.4,229.5)	956 (647,1332)	123.2 (83.4,171.4)	2.4 (-6,11.2)
State of Libya	6383 (4458,9120)	144.8 (101.8,205.8)	13943 (9445,19713)	167.8 (113.2,238.4)	1.9 (-6.7,11.1)
Republic of Sudan	28650 (19588,41205)	134.8 (92.2,192.4)	183526 (125202,257161)	110.3 (75.4,154.6)	2.5 (-6.1,13.6)
Democratic Republic of Timor-Leste	1037 (696,1448)	124.8 (84.4,174.2)	44504 (30378,61385)	164.2 (112.5,227.7)	0 (-9.3,9.8)
Republic of Mauritius	1511 (1032,2125)	134.1 (91.7,188.3)	17122 (11851,23751)	165.5 (115.3,231.2)	4.4 (-4.8,13.8)
Republic of Slovenia	3526 (2421,4936)	182.8 (125.9,256.5)	65019 (43430,90405)	145.2 (97.3,202)	1.4 (-7.4,10.9)
Slovak Republic	9236 (6303,12725)	176.8 (120.6,244.1)	10929 (7592,15387)	175.7 (121.8,249.1)	1.4 (-0.7,3.3)
Kingdom of Thailand	76503 (53266,109395)	131 (91,187.7)	288 (193,407)	130.9 (87.8,184.5)	2.9 (-4.8,12.1)
Republic of Peru	28168 (19267,39385)	124.7 (85.5,174.6)	70109 (47445,98658)	145 (98.1,204.3)	0 (-7.9,9.6)
Togolese Republic	6382 (4326,9032)	162 (109.6,228)	6418 (4433,9111)	198.6 (137.5,283.6)	2.3 (-6.4,12.6)
Republic of Paraguay	4913 (3367,6862)	116.2 (79.8,162)	21944 (15139,30974)	135.4 (93.7,191.1)	2.3 (-5.8,11.4)
Republic of Guyana	1016 (699,1428)	123.3 (85.1,172.6)	3673 (2526,5180)	190.9 (129.3,268.9)	2.7 (-6,11.9)
Republic of Finland	7449 (5070,10523)	155.4 (105.7,220.8)	27087 (18603,38384)	163 (111.9,229.6)	3.8 (-5.7,14.9)
Republic of Guinea	10247 (7152,14497)	160.9 (112.7,227.5)	193673 (133619,271797)	167.1 (114.8,234.2)	4.6 (-0.4,9.6)
Republic of South Africa	64565 (44800,91297)	166.3 (115.6,234.2)	18545 (12872,25998)	187 (131.1,261.6)	2.1 (-6.6,11.4)
Republic of Cyprus	1277 (867,1789)	164.9 (111.8,231.1)	342925 (230158,484117)	169.4 (116.7,234.6)	0.1 (-8.9,9.3)
Kingdom of Denmark	8410 (5849,11672)	172.4	1039 (719,1435)	280.9 (193.9,388)	1.4 (-7.9,10.2)

		(119.7,238.6)			
Republic of El Salvador	7583 (5418,10626)	136.1 (97.1,190.5)	15599 (10775,21490)	122 (81.9,172)	-1 (-10.1,8.1)
People's Republic of Bangladesh	125391 (85921,176640)	108.1 (74,152)	130817 (90463,181611)	168.4 (116.2,233.5)	1.9 (-6.7,11.5)
Republic of Costa Rica	4671 (3212,6577)	148.2 (101.8,208.6)	9025 (6119,12601)	139.5 (94.5,194.8)	-0.4 (-8.7,8.2)
Kingdom of Norway	6123 (4208,8619)	151.9 (104.3,213.7)	3180 (2157,4428)	168.5 (114.2,234.2)	3.7 (-4.8,13.1)
People's Democratic Republic of Algeria	37870 (25640,53393)	142.3 (96.5,201.1)	23353 (15913,32436)	166.5 (113.2,232.4)	0.6 (-9,10.4)
State of Qatar	817 (567,1148)	174.1 (120.8,244.9)	9636 (6517,13458)	141.5 (95.9,197.8)	2.4 (-6.9,10.8)
Gabonese Republic	1704 (1170,2351)	164.7 (112.5,227.3)	18384 (12565,25850)	191.3 (130.9,270)	-0.9 (-9,7.9)
Federal Republic of Somalia	14120 (9692,19971)	162.9 (111.7,230)	1756 (1199,2439)	130.7 (89.3,181.4)	1.7 (-2,5.1)
Kingdom of the Netherlands	24409 (16923,34472)	168.7 (116.8,238)	156034 (105617,220041)	143 (96.7,202.4)	0.5 (-7.2,11.4)
Republic of India	1168136 (803220,1647739)	130.1 (89.6,183.1)	9667 (6559,13462)	167.4 (114.2,232.3)	-1.2 (-10.6,7.6)
Republic of Benin	8437 (5903,11898)	160.9 (112.7,225.4)	121955 (82458,170704)	147.6 (99.7,207)	2.3 (-6.8,11.3)
Republic of Colombia	47678 (32948,67939)	140.8 (97.2,200)	15 (10,21)	126.1 (86,177.8)	1.8 (-8.3,11.2)
Republic of Burundi	9602 (6440,13646)	160.3 (107.9,225.4)	1308 (913,1831)	170.1 (118.7,238.1)	3.3 (-6.5,13.3)
Republic of Albania	5855 (4003,8261)	171.8 (117.8,243)	38420 (26189,54091)	162.2 (111.3,227.5)	0.6 (-8,11.5)
Republic of Vanuatu	205 (141,284)	127.4 (87.7,176.7)	3015 (2093,4222)	176.9 (122.6,248)	3.1 (-4.7,13)
Republic of Haiti	7874 (5531,11110)	115.8 (80.8,162.7)	31313 (21435,44203)	163.3 (112.9,229.6)	4.3 (-4.4,13.7)
Portuguese Republic	15511 (10693,21735)	158.5 (109.3,222.4)	5693 (3911,7937)	180.6 (123.6,251.2)	2.1 (-6.2,11.8)
Republic of Mozambique	22813 (15366,31712)	158.9 (106.9,221.4)	2 (1,3)	131.6 (89.9,184)	-1.8 (-10.2,7.1)
Islamic Republic of Afghanistan	13486 (9305,18960)	127.7 (88,180.1)	392 (261,559)	173.6 (116,247.1)	2.5 (-6.2,11.6)
Republic of Liberia	4225 (2904,5978)	161.2 (110.9,226.9)	1887143 (1285675,2649048)	131.9 (89.8,185.2)	3.4 (-4.4,12)
Kingdom of Eswatini	1426 (961,1991)	163.6 (110.5,228.3)	59138 (40825,84470)	152.8 (104.9,218.5)	2.3 (-5.6,10.5)

Republic of Rwanda	12502 (8564,17492)	160.6 (109.4,224.6)	153 (106,215)	121.3 (83.7,170.1)	2.2 (-6,10.8)
French Republic	90966 (61087,125628)	163.8 (110.4,227.1)	2156 (1465,3032)	166 (112.3,233.2)	3.1 (-5.3,12.3)
Kingdom of Cambodia	13169 (8820,18261)	119.6 (80.8,166)	23381 (16201,32782)	165.7 (114.8,232.7)	3.4 (-0.8,7.4)
Republic of Equatorial Guinea	717 (495,1013)	156.6 (108.6,221.6)	137543 (95424,192945)	137.3 (95.2,193.1)	2.3 (-5.4,11.1)
Republic of Uganda	30437 (21032,43111)	163.1 (113.5,229.5)	28088 (19509,39427)	168.1 (117.5,235.9)	2 (-7.2,14.8)
Republic of Guinea-Bissau	1724 (1190,2448)	158 (108.1,222.9)	1700739 (1146590,2397647)	124.2 (83.7,175.1)	2.9 (-5.6,11.2)
Islamic Republic of Pakistan	138275 (94026,191479)	117.4 (79.9,162.8)	22078 (14960,31395)	126.4 (85.8,179.8)	1.8 (-7.2,12.4)
Socialist Republic of Viet Nam	92039 (63221,129977)	129.2 (88.8,182.5)	11780 (7913,16664)	167.2 (112.3,236)	0.7 (-7.9,10.2)
Republic of Indonesia	229457 (157216,322857)	184.9 (128.8,261.3)	23975 (16662,33538)	166.2 (115.6,231)	1.6 (-8.5,11.1)
Kingdom of Spain	69217 (48165,97866)	152.6 (103.1,212.2)	7922 (5419,11241)	171.6 (117,243.7)	3.4 (-5.3,13.1)
Sultanate of Oman	3187 (2159,4453)	163.1 (114.5,227.6)	57 (39,80)	167.3 (115.2,235.1)	0 (-3.9,4.1)
Republic of Cameroon	18253 (12805,25571)	118.6 (81.5,166.7)	27115 (18999,38138)	169 (117.4,236.9)	0.4 (-1.6,2.3)
Kingdom of Bahrain	817 (557,1146)	153.1 (105,214.6)	24 (16,34)	136.1 (92,191.8)	2.8 (-6.1,12.6)
Republic of Zimbabwe	18444 (12568,25882)	166.6 (114.4,234.4)	772 (515,1086)	144.4 (97.2,203.4)	1.9 (-7.5,10.6)
Republic of Niue	3 (2,4)	130.8 (90.2,184.5)	126162 (85751,178256)	147.8 (100.6,208.8)	2.6 (-5.2,10.2)
Republic of Djibouti	755 (515,1053)	170.3 (117.3,237.6)	87303 (59403,122255)	196.3 (133.8,274.6)	0.8 (-8.6,11)
Republic of Guatemala	11906 (8220,16751)	132.4 (91.5,186.7)	74 (51,104)	126.6 (88.6,179.3)	1.2 (-6.7,11.4)
Federal Democratic Republic of Nepal	23950 (16558,33224)	115.6 (79.8,161)	661 (458,917)	196.9 (136.2,273.2)	4.1 (-5.7,14.1)
Republic of Lithuania	6256 (4243,8800)	174.1 (117.9,245.2)	405272 (277499,571892)	164.6 (112.9,231.7)	0.7 (-8.7,9.9)
Republic of South Sudan	10447 (7179,14861)	167.1 (114.7,236.3)	14416 (9861,20196)	135.7 (92.9,190.6)	1.8 (-6.1,12)
Republic of Armenia	5939 (4052,8353)	170.3 (116.4,239.1)	719 (492,1003)	125.5 (85.8,175.1)	2.3 (-5.6,11.1)

Kingdom of Bhutan	802 (548,1135)	119.1 (81.2,169.7)	34642 (23757,48771)	166.4 (114.6,234.3)	3.6 (-4.7,12.1)
Republic of Latvia	4421 (2961,6263)	172.2 (115.7,243.9)	135 (91,190)	128.3 (87,180.8)	4.3 (-4.2,14.3)
Republic of Seychelles	99 (69,138)	133.7 (93.2,186.2)	1012 (695,1423)	178.5 (122.8,251.3)	1.4 (-1.2,4)
Republic of Singapore	8347 (5735,11712)	269.8 (184.9,377.5)	32106 (22340,45639)	168.5 (117.3,239.3)	-0.7 (-10.5,8.2)
Republic of the Marshall Islands	62 (42,85)	127.1 (86.9,175)	183348 (123704,257839)	142.2 (95.9,199.9)	0.1 (-5.1,5.5)
Republic of Kazakhstan	27661 (19048,39202)	164.7 (113.8,233.5)	4411 (3032,6136)	177.3 (121.5,247.7)	3 (-6.4,11.9)
Islamic Republic of Mauritania	3613 (2484,5146)	165.5 (114.1,235.2)	17144 (11666,24099)	146.6 (99.6,206.8)	2.9 (-5.9,11.6)
Kingdom of Saudi Arabia	24723 (17116,34818)	148.3 (103.1,209)	11664 (8068,16478)	165.7 (114.8,233.7)	0.1 (-8.4,9.7)
Union of the Comoros	822 (572,1169)	165.9 (114.9,234.8)	14682 (10186,20530)	165.8 (115.4,230.2)	1.2 (-7.7,10.6)
Republic of Bulgaria	14537 (9805,20311)	173.3 (117.2,242.3)	14908 (10362,20975)	123.9 (86.2,174)	2.3 (-7.2,13.2)
Republic of Azerbaijan	12420 (8386,17541)	164.1 (110.9,232.1)	54316 (37638,75615)	161.8 (111.9,225.7)	3.2 (-4.5,10.3)
Federal Republic of Germany	124845 (83870,175275)	165.3 (111,232.3)	15183 (10277,21413)	165.3 (111.5,233.2)	1.5 (-8.3,10.8)
Republic of the Niger	14043 (9754,19755)	161.9 (113.1,226.2)	56435 (38499,79141)	167 (114.3,232.9)	0.3 (-7.8,9.4)
Republic of Mali	14937 (10284,20997)	161 (110.8,225.6)	44251 (30327,62061)	130.4 (89,183.2)	-0.2 (-8,9.5)
Republic of Nauru	14 (10,19)	128.2 (89.4,178.9)	78396 (54902,109955)	167.9 (118.2,234.4)	-1.7 (-10.5,7.1)
Czech Republic	18029 (12491,25101)	180.3 (125,251.8)	56279 (38561,78842)	132 (90.6,184.9)	2.1 (-7.2,12.1)
Republic of Panama	3546 (2394,4955)	143.8 (97.2,201.6)	6257 (4321,8866)	146.3 (101.1,207.4)	1.7 (-7.7,9.8)
Republic of Fiji	1021 (705,1423)	128.5 (88.2,179.2)	8533 (5741,11754)	117.4 (79,161.8)	-0.9 (-9.9,7.9)
Republic of Kiribati	96 (66,134)	121 (82.9,168.9)	13726 (9412,19165)	125.4 (86.1,174.8)	3.6 (-7.2,14.6)
Principality of Monaco	46 (31,64)	168.4 (115.6,236)	149400 (101109,210256)	129 (87.3,181.6)	-2.2 (-10.5,5.1)
Republic of Estonia	2642 (1842,3672)	173.5 (120.9,240.9)	37364 (25892,52416)	142 (98.5,199.8)	2.3 (-7,12.4)
Republic of Croatia	8640 (5910,11996)	181.6	14104 (9652,19849)	149.9	6.3 (-2.6,17)

		(123.8,252.2)		(102.6,211.2)	
Republic of Belarus	17589 (12181,24668)	172.3 (119,242.1)	2807 (1915,4027)	172.9 (118.2,247.2)	1.4 (-6.9,10.4)
Republic of Honduras	6853 (4782,9638)	136.3 (95.3,190.8)	2009 (1388,2809)	164.4 (114.2,229.1)	4.3 (-6.1,16.7)
Federal Republic of Nigeria	155454 (106316,218498)	163.9 (112.4,230.6)	8901 (5936,12446)	162.5 (108.4,226.9)	2.4 (-5.9,12)
Commonwealth of the Bahamas	346 (238,479)	130 (89.2,179.9)	89120 (60590,125116)	140.6 (95.4,198.1)	2.3 (-6,12.1)
Arab Republic of Egypt	81337 (56168,114586)	139.8 (96.4,196.4)	85 (59,122)	130.2 (90.1,185.4)	9 (6.6,11.5)
Republic of Suriname	505 (348,707)	126.2 (87,176.7)	7266 (4864,10244)	155 (104.3,218.7)	0.7 (-7.6,10.1)
Kingdom of Sweden	14970 (10197,21127)	185.3 (126.1,262.1)	290211 (200628,405965)	117.5 (81.3,164.4)	2.3 (-6.1,11.1)
Republic of Iceland	486 (336,687)	193.3 (133.9,273.2)	1206 (829,1731)	128.4 (88.2,184.2)	-0.5 (-9,9)
Republic of Tunisia	12613 (8634,17545)	145.7 (99.6,202.6)	23310 (15653,32407)	128.2 (86.2,178.4)	3 (-6.1,12.5)
Islamic Republic of Iran	86572 (58947,120628)	143.9 (97.8,200.6)	2289 (1615,3245)	174.7 (123.3,247.3)	0.5 (-8.5,9.6)
State of Eritrea	5944 (4111,8298)	160 (111.7,222.6)	36926 (25392,52667)	202.8 (139.6,289.4)	3.6 (-5.6,13.5)
United Mexican States	125303 (86065,174717)	139.4 (95.9,194)	101036 (69889,141835)	187.7 (129.6,263.8)	1.6 (-8.2,10)
Swiss Confederation	10972 (7484,15494)	167.3 (114.4,236.4)	17600 (12253,24508)	168.1 (117.3,234.1)	3 (-5.6,12.8)
Republic of Angola	17892 (12655,25001)	161.4 (113.9,226.1)	50 (35,70)	167.5 (114.9,234)	1.7 (-6.2,9.9)
Democratic Republic of Sao Tome and Principe	216 (152,306)	168.4 (118.1,237)	137 (95,192)	170.1 (116.1,239.3)	-0.6 (-11.3,7.2)
Hellenic Republic	16603 (11316,23540)	166.2 (113.5,236.4)	2190 (1509,3016)	181 (124.1,249)	1.2 (-7.5,11.6)
Republic of Cuba	14633 (9974,20551)	134.7 (91.8,189.1)	498 (339,694)	129.8 (88.2,180.9)	3.2 (-7,12.7)
Argentine Republic	64278 (43927,89923)	193.2 (132.1,270.2)	47867 (33123,68346)	134.8 (93.4,191.5)	3.3 (-5.3,13.3)
Federated States of Micronesia	140 (96,197)	127.5 (87,179.5)	14041 (9780,19662)	169.3 (117.5,235.9)	2.5 (-6.7,11.5)
State of Israel	7464 (5219,10361)	149.7 (104.7,207.7)	7298 (4986,10302)	187.1 (127.5,264.5)	0.6 (-8.8,9.3)
Republic of Zambia	13876 (9592,19187)	162.3	142 (98,202)	130.7	5.6 (-5,16.1)

		(112.5,224.9)		(89.5,185.5)	
Kyrgyz Republic	7570 (5281,10576)	163.5 (114.4,229.6)	3175 (2193,4366)	161.2 (111.2,222.2)	3.7 (-5.4,12.6)
Republic of Turkey	85264 (58627,119175)	143 (98.5,200.6)	15254 (10484,21344)	182.1 (125.7,254.1)	3.8 (-6.1,13.3)
Republic of Cabo Verde	632 (439,886)	169.9 (117.5,236.9)	9415 (6499,13233)	166.8 (115.3,234.3)	0.2 (-8.8,8.5)
Republic of Kenya	41838 (28594,58781)	167.8 (114.8,235.1)	60404 (40600,84566)	167.6 (112,234.3)	2 (-6.2,10.9)
Republic of Iraq	25205 (17101,35300)	129.1 (88.1,179.2)	50561 (35257,70976)	166.5 (116.2,231.6)	1.1 (-7.6,9.6)
Republic of Palau	21 (14,29)	132 (90.8,187.7)	57588 (39254,81168)	165.1 (112.3,232.7)	2 (-0.7,4.9)
Hashemite Kingdom of Jordan	5876 (4019,8403)	148.6 (101.5,212.8)	4279 (2930,6026)	168.8 (115.9,237.5)	1.2 (-0.5,3.1)
Republic of Nicaragua	5731 (3910,8071)	138.5 (94.2,195.8)	9959 (6741,14056)	143.6 (97.2,202.8)	7.2 (3.1,11.5)
Republic of Trinidad and Tobago	1609 (1098,2240)	129.8 (88.6,180.7)	30838 (21382,43324)	140.8 (97.4,197.9)	0.5 (-7.7,9.9)
United Kingdom of Great Britain and Northern Ireland	70242 (48077,98631)	129 (88.3,181.1)	34398 (23413,48405)	165.6 (112.8,232.8)	1 (-6.7,10.3)
Republic of Maldives	305 (204,436)	130.7 (87.6,187.2)	686 (462,963)	168.9 (113.8,236.8)	3.3 (-6.4,12.5)
Independent State of Papua New Guinea	5437 (3703,7659)	125.4 (85.7,177)	17685 (12069,24824)	166.2 (113.2,232.5)	2.2 (-6.8,11.6)
Republic of Chad	10350 (7167,14469)	159.8 (110.9,224.5)	97378 (66684,136767)	169.1 (115.8,237.7)	1.1 (-8.6,9.9)
Republic of Botswana	2326 (1623,3264)	164.6 (115.2,228.2)	4252 (2959,5888)	167.6 (116.8,232.6)	1.7 (-7,12.1)
Federal Democratic Republic of Ethiopia	87689 (60603,123244)	159.7 (110.5,224.4)	1674 (1138,2339)	136.3 (92.5,190.6)	3.9 (-5.6,13.2)
Republic of Côte d'Ivoire	21509 (14578,29995)	164.1 (111.4,229.5)	52698 (36207,73556)	141.2 (97,197)	2.1 (-7.5,10.7)
Republic of Senegal	13329 (9205,18761)	163.5 (112.8,228.8)	23384 (15957,32676)	163.2 (111,228.8)	1.4 (-7.2,9.1)

location	Prevalence (95% CI)	Incidence (95% CI)	DALYs (95% CI)
Global	0.07 (0.07,0.08)	0.13 (0.11,0.14)	0.08 (0.08,0.09)
Low SDI	0.08 (0.07,0.08)	0.08 (0.07,0.08)	0.11 (0.1,0.12)
Low-middle SDI	0.08 (0.07,0.08)	0.15 (0.14,0.16)	0.1 (0.09,0.1)
Middle SDI	0.17 (0.16,0.18)	0.09 (0.07,0.11)	0.18 (0.17,0.19)
High-middle SDI	0.11 (0.1,0.12)	0.06 (0.02,0.1)	0.12 (0.11,0.13)
High SDI	0.06 (0.05,0.08)	-0.02 (-0.05,0.01)	0.06 (0.04,0.07)
East Asia	0.24 (0.22,0.27)	-0.05 (-0.1,0)	0.25 (0.23,0.27)
Southeast Asia	0.1 (0.09,0.11)	0.02 (0.01,0.04)	0.12 (0.11,0.13)
Oceania	-0.03 (-0.03,-0.02)	-0.02 (-0.04,0)	-0.02 (-0.02,-0.02)
Central Asia	0.05 (0.04,0.06)	0.04 (0.03,0.05)	0.06 (0.05,0.07)
Central Europe	0.11 (0.11,0.11)	0 (-0.01,0.01)	0.12 (0.12,0.13)
Eastern Europe	0.09 (0.08,0.1)	0.06 (0.06,0.07)	0.1 (0.09,0.12)
High-income Asia Pacific	0.25 (0.23,0.27)	0.17 (0.14,0.19)	0.25 (0.23,0.28)
Australasia	0.08 (0.08,0.09)	0.13 (0.1,0.16)	0.09 (0.08,0.09)
Western Europe	0.09 (0.08,0.09)	0.01 (-0.01,0.03)	0.09 (0.08,0.09)
Southern Latin America	0.05 (0.04,0.05)	0.02 (-0.02,0.06)	0.05 (0.04,0.05)
High-income North America	0.1 (0.08,0.13)	0.07 (0.04,0.1)	0.09 (0.06,0.11)
Caribbean	-0.01 (-0.02,-0.01)	-0.06 (-0.07,-0.05)	-0.01 (-0.02,-0.01)
Andean Latin America	0.1 (0.1,0.11)	-0.02 (-0.04,0.01)	0.12 (0.11,0.12)
Central Latin America	0.06 (0.05,0.06)	-0.01 (-0.03,0)	0.06 (0.06,0.06)
Tropical Latin America	0.03 (0.03,0.04)	0.03 (0.01,0.05)	0.05 (0.04,0.05)
North Africa and Middle East	0.07 (0.07,0.08)	-0.01 (-0.02,0.01)	0.08 (0.07,0.08)
South Asia	0.01 (0,0.02)	0.1 (0.07,0.13)	0.03 (0.03,0.04)
Central Sub-Saharan Africa	0.07 (0.06,0.08)	-0.03 (-0.03,-0.03)	0.11 (0.09,0.12)
Eastern Sub-Saharan Africa	0.07 (0.06,0.07)	-0.01 (-0.01,-0.01)	0.1 (0.09,0.11)
Southern Sub-Saharan Africa	0.06 (0.04,0.07)	-0.01 (-0.01,0)	0.05 (0.03,0.07)
Western Sub-Saharan Africa	0.03 (0.02,0.03)	0.01 (0,0.01)	0.05 (0.05,0.06)

Location	Year	Val	SDI	Frontier	Difference	Trend
Afghanistan	2021	130.4034	0.3372	108.4295	21.97387	Increase
Albania	2021	177.5461	0.70685	108.3788	69.16731	Increase
Algeria	2021	145.247	0.659501	108.3519	36.89507	Increase
American Samoa	2021	132.6652	0.723728	108.4778	24.18736	Decrease
Andorra	2021	170.0793	0.869444	108.4591	61.62028	Decrease
Angola	2021	163.7542	0.453722	108.3423	55.41185	Increase
Antigua and Barbuda	2021	133.1906	0.749887	108.4138	24.77684	Increase
Argentina	2021	196.2566	0.723123	108.4168	87.8398	Increase
Armenia	2021	173.1395	0.701833	108.2397	64.89979	Increase
Australia	2021	223.5158	0.844253	108.2645	115.2513	Increase
Austria	2021	167.7508	0.853837	108.3119	59.43888	Increase
Azerbaijan	2021	168.1384	0.694851	108.2407	59.89767	Increase
Bahamas	2021	129.7904	0.805021	108.3143	21.4761	Decrease
Bahrain	2021	158.7021	0.753043	108.3412	50.36085	Increase
Bangladesh	2021	110.2609	0.492421	108.4994	1.761575	Increase
Barbados	2021	133.5623	0.746749	108.3073	25.25496	Increase
Belarus	2021	177.3006	0.784485	108.288	69.01258	Increase
Belgium	2021	166.2241	0.853654	108.1742	58.04985	Increase
Belize	2021	127.8496	0.610229	108.3642	19.48542	Decrease
Benin	2021	166.2208	0.373487	108.2629	57.95796	Increase
Bermuda	2021	139.1652	0.821365	108.3076	30.8576	Increase
Bhutan	2021	121.3216	0.473062	108.4218	12.89985	Increase
Bolivia (Plurinational State of)	2021	123.9215	0.599011	108.3106	15.61084	Increase
Bosnia and Herzegovina	2021	182.2513	0.723078	108.3701	73.88115	Increase
Botswana	2021	168.3644	0.642722	108.2639	60.10056	Increase
Brazil	2021	114.3472	0.653044	108.3916	5.955655	Increase
Brunei Darussalam	2021	257.7227	0.810234	108.3465	149.3762	Increase
Bulgaria	2021	175.7374	0.768151	108.3375	67.39985	Increase
Burkina Faso	2021	163.6868	0.285118	108.2402	55.44654	Increase
Burundi	2021	165.6769	0.289374	108.3028	57.37409	Increase
Cabo Verde	2021	178.4988	0.533535	108.3407	70.15819	Increase
Cambodia	2021	126.3913	0.473621	108.3745	18.01685	Increase
Cameroon	2021	166.9763	0.479691	108.3265	58.64981	Increase
Canada	2021	209.5559	0.873171	108.2715	101.2844	Increase
Central African Republic	2021	157.3805	0.309168	108.487	48.89357	Increase
Chad	2021	163.3092	0.240436	108.4366	54.87267	Increase
Chile	2021	202.8089	0.771515	108.2991	94.50973	Increase
China	2021	124.1893	0.72163	108.5056	15.68376	Increase
Colombia	2021	145.0215	0.655443	108.361	36.66047	Increase
Comoros	2021	170.1199	0.475979	108.3164	61.80353	Increase
Congo	2021	166.7876	0.583075	108.2383	58.54935	Increase
Cook Islands	2021	135.8949	0.77911	108.3537	27.54116	Decrease
Costa Rica	2021	148.5954	0.70034	108.3695	40.22587	Increase
Croatia	2021	187.1057	0.798341	108.2791	78.8266	Increase
Cuba	2021	135.7019	0.66873	108.3034	27.39852	Increase
Cyprus	2021	166.0292	0.835631	108.3527	57.67646	Increase
Czechia	2021	186.9752	0.82845	108.4596	78.51561	Increase
Côte d'Ivoire	2021	169.8959	0.425942	108.3141	61.5818	Increase
Democratic People's Republic of Korea	2021	133.9264	0.569855	108.3454	25.58105	Increase
Democratic Republic of the Congo	2021	165.0544	0.38318	108.2265	56.82789	Increase
Denmark	2021	177.6991	0.896424	108.3966	69.30245	Increase
Djibouti	2021	174.7465	0.487958	108.3154	66.43106	Increase
Dominica	2021	130.2103	0.746967	108.5253	21.68501	Increase

Dominican Republic	2021	126.938	0.619388	108.3519	18.58617	Increase
Ecuador	2021	128.2083	0.661017	108.348	19.86037	Increase
Egypt	2021	143.0424	0.606787	108.3347	34.70775	Increase
El Salvador	2021	139.509	0.563775	108.3579	31.15105	Increase
Equatorial Guinea	2021	172.9383	0.657857	108.4293	64.50904	Increase
Eritrea	2021	165.6654	0.403864	108.3342	57.33125	Increase
Estonia	2021	181.0072	0.844918	108.2529	72.75433	Increase
Eswatini	2021	164.4236	0.58546	108.3246	56.09901	Increase
Ethiopia	2021	167.1373	0.358823	108.275	58.86224	Increase
Fiji	2021	128.4134	0.675052	108.4044	20.00894	Decrease
Finland	2021	155.4886	0.859831	108.3181	47.17047	Increase
France	2021	165.1595	0.838365	108.3007	56.85879	Increase
Gabon	2021	168.4523	0.634691	108.3145	60.13778	Increase
Gambia	2021	167.6347	0.409714	108.2657	59.36901	Increase
Georgia	2021	170.9284	0.732474	108.3812	62.54716	Increase
Germany	2021	168.3769	0.902957	108.225	60.15194	Increase
Ghana	2021	167.6461	0.56493	108.376	59.27009	Increase
Greece	2021	165.2975	0.791854	108.3274	56.97008	Decrease
Greenland	2021	190.3842	0.82621	108.3376	82.04657	Increase
Grenada	2021	130.4819	0.668993	108.2304	22.25146	Increase
Guam	2021	136.781	0.803982	108.4063	28.37476	Decrease
Guatemala	2021	135.3684	0.539972	108.3565	27.01185	Increase
Guinea	2021	163.1976	0.336401	108.33	54.86759	Increase
Guinea-Bissau	2021	161.3862	0.35311	108.38	53.00618	Increase
Guyana	2021	123.1986	0.650812	108.2301	14.96853	Decrease
Haiti	2021	116.5396	0.448278	108.2578	8.281834	Increase
Honduras	2021	136.2493	0.513037	108.2589	27.99038	Decrease
Hungary	2021	182.5401	0.790755	108.2266	74.31354	Increase
Iceland	2021	196.91	0.876362	108.4555	88.45447	Increase
India	2021	131.9046	0.575402	108.4472	23.45741	Increase
Indonesia	2021	122.0318	0.656868	108.2527	13.77915	Increase
Iran (Islamic Republic of)	2021	147.7806	0.697207	108.3738	39.40673	Increase
Iraq	2021	132.0195	0.662626	108.3447	23.67481	Increase
Ireland	2021	200.9013	0.873754	108.2709	92.63042	Increase
Israel	2021	149.8649	0.809012	108.2708	41.59411	Increase
Italy	2021	187.699	0.805774	108.3576	79.34141	Increase
Jamaica	2021	131.7831	0.683263	108.3577	23.42537	Increase
Japan	2021	299.1432	0.871242	108.3096	190.8337	Increase
Jordan	2021	151.7181	0.725307	108.1677	43.55036	Increase
Kazakhstan	2021	168.5489	0.725144	108.3542	60.19471	Increase
Kenya	2021	169.704	0.523768	108.3715	61.33242	Increase
Kiribati	2021	121.304	0.527187	108.4054	12.89866	Increase
Kuwait	2021	155.0363	0.846651	108.3223	46.71395	Decrease
Kyrgyzstan	2021	167.2461	0.603979	108.2702	58.97594	Increase
Lao People's Democratic Republic	2021	126.1165	0.489136	108.3801	17.73638	Increase
Latvia	2021	176.8706	0.830664	108.318	68.55262	Increase
Lebanon	2021	162.4796	0.744746	108.3018	54.17779	Increase
Lesotho	2021	161.2058	0.510393	108.3369	52.86891	Increase
Liberia	2021	167.4378	0.352442	108.2833	59.15446	Increase
Libya	2021	143.5507	0.725771	108.266	35.28469	Decrease
Lithuania	2021	177.3031	0.856484	108.2748	69.02832	Increase
Luxembourg	2021	169.4246	0.884429	108.2784	61.14624	Increase
Madagascar	2021	166.4672	0.400247	108.4346	58.0326	Increase
Malawi	2021	165.5621	0.384554	108.38	57.18209	Increase

Malaysia	2021	138.1526	0.742524	108.2718	29.88073	Increase
Maldives	2021	144.3666	0.650887	108.2411	36.12553	Increase
Mali	2021	164.8247	0.26858	108.2377	56.58704	Increase
Malta	2021	168.9308	0.801585	108.315	60.61581	Increase
Marshall Islands	2021	126.6416	0.574091	108.2714	18.3702	Decrease
Mauritania	2021	171.5771	0.498945	108.4399	63.13717	Increase
Mauritius	2021	136.3344	0.71826	108.3645	27.96995	Increase
Mexico	2021	142.1687	0.664575	108.4231	33.74561	Increase
Micronesia (Federated States of)	2021	128.3002	0.587535	108.2814	20.01881	Increase
Monaco	2021	167.2562	0.908263	108.3142	58.94203	Decrease
Mongolia	2021	161.2405	0.617622	108.3311	52.90941	Increase
Montenegro	2021	183.4548	0.795801	108.3297	75.12512	Increase
Morocco	2021	141.174	0.562698	108.2559	32.91818	Increase
Mozambique	2021	161.7823	0.326463	108.2266	53.55575	Increase
Myanmar	2021	125.8055	0.533901	108.2046	17.60091	Increase
Namibia	2021	168.7678	0.617565	108.3025	60.46539	Increase
Nauru	2021	126.0637	0.625178	108.2722	17.79152	Decrease
Nepal	2021	116.8807	0.433175	108.1703	8.710387	Increase
Netherlands	2021	169.0383	0.888464	108.2713	60.76706	Increase
New Zealand	2021	218.2545	0.849442	108.3273	109.9272	Increase
Nicaragua	2021	141.4978	0.523958	108.4623	33.03547	Increase
Niger	2021	164.2301	0.168073	159.7709	4.459107	Increase
Nigeria	2021	164.6136	0.503391	108.2828	56.33083	Increase
Niue	2021	131.5998	0.726222	108.3679	23.23197	Increase
North Macedonia	2021	179.615	0.75063	108.326	71.28897	Increase
Northern Mariana Islands	2021	138.0736	0.771535	108.2092	29.86438	Decrease
Norway	2021	151.8718	0.916133	108.3757	43.49606	Increase
Oman	2021	156.127	0.773392	108.25	47.87706	Increase
Pakistan	2021	117.5222	0.504029	108.2356	9.286552	Increase
Palau	2021	136.0782	0.754047	108.2304	27.84775	Increase
Palestine	2021	145.8699	0.631012	108.3711	37.49887	Increase
Panama	2021	146.327	0.708865	108.3385	37.98845	Increase
Papua New Guinea	2021	125.3757	0.417797	108.3194	17.05637	Decrease
Paraguay	2021	117.4281	0.635718	108.3119	9.116264	Increase
Peru	2021	130.1	0.662054	108.2282	21.87182	Increase
Philippines	2021	128.9797	0.651219	108.1947	20.78498	Increase
Poland	2021	180.8635	0.812043	108.3264	72.53716	Increase
Portugal	2021	161.4668	0.744152	108.3899	53.07682	Increase
Puerto Rico	2021	136.9129	0.825526	108.3275	28.58541	Increase
Qatar	2021	180.6418	0.846861	108.3078	72.33397	Increase
Republic of Korea	2021	283.8044	0.886675	108.3035	175.5009	Increase
Republic of Moldova	2021	172.53	0.732215	108.4138	64.11622	Increase
Romania	2021	176.5415	0.768454	108.2644	68.277	Increase
Russian Federation	2021	173.8836	0.808536	108.2086	65.67496	Increase
Rwanda	2021	166.5201	0.435589	108.4065	58.11366	Increase
Saint Kitts and Nevis	2021	130.0573	0.754987	108.3382	21.7191	Increase
Saint Lucia	2021	131.2461	0.67251	108.4546	22.79146	Increase
Saint Vincent and the Grenadines	2021	129.6611	0.637196	108.3192	21.34192	Increase
Samoa	2021	130.9395	0.593393	108.4344	22.50511	Increase
San Marino	2021	167.4562	0.888005	108.3267	59.12958	Decrease
Sao Tome and Principe	2021	173.5589	0.505414	108.3421	65.2168	Increase
Saudi Arabia	2021	152.7572	0.815143	108.3098	44.44734	Increase
Senegal	2021	168.0567	0.408054	108.3265	59.73023	Increase
Serbia	2021	182.1472	0.792416	108.2856	73.86164	Increase

Seychelles	2021	138.1516	0.730151	108.2856	29.86603	Increase
Sierra Leone	2021	166.0261	0.358666	108.3106	57.7155	Increase
Singapore	2021	280.9487	0.856098	108.3469	172.6017	Increase
Slovakia	2021	181.4171	0.810611	108.3067	73.11037	Increase
Slovenia	2021	190.8628	0.842431	108.2327	82.63019	Increase
Solomon Islands	2021	126.0744	0.42936	108.3613	17.71301	Decrease
Somalia	2021	162.2023	0.077688	161.418	0.78433	Decrease
South Africa	2021	169.076	0.679627	108.278	60.79808	Increase
South Sudan	2021	165.4847	0.278371	108.317	57.16771	Decrease
Spain	2021	192.8078	0.769284	108.2379	84.56992	Increase
Sri Lanka	2021	140.7999	0.701535	108.4078	32.39214	Increase
Sudan	2021	137.5164	0.54195	108.3944	29.12196	Increase
Suriname	2021	125.5427	0.633666	108.2086	17.33411	Decrease
Sweden	2021	191.3208	0.88688	108.3162	83.00459	Increase
Switzerland	2021	169.3472	0.933059	108.2593	61.08783	Increase
Syrian Arab Republic	2021	141.1282	0.623004	108.3443	32.78388	Decrease
Taiwan (Province of China)	2021	172.2441	0.874747	108.3844	63.85973	Increase
Tajikistan	2021	162.9377	0.541511	108.345	54.59273	Increase
Thailand	2021	135.9777	0.682548	108.1852	27.79253	Increase
Timor-Leste	2021	126.8561	0.444668	108.1949	18.66122	Increase
Togo	2021	165.8278	0.408534	108.3433	57.48449	Increase
Tokelau	2021	134.1355	0.686426	108.3442	25.79136	Increase
Tonga	2021	130.7221	0.62635	108.5042	22.21792	Increase
Trinidad and Tobago	2021	130.7327	0.768763	108.3075	22.42521	Increase
Tunisia	2021	146.595	0.682432	108.3466	38.24834	Increase
Turkmenistan	2021	166.4597	0.682161	108.3162	58.14347	Increase
Tuvalu	2021	128.97	0.576621	108.2569	20.7131	Increase
Türkiye	2021	147.6445	0.712693	108.3866	39.25797	Increase
Uganda	2021	167.8897	0.423261	108.3022	59.5875	Increase
Ukraine	2021	172.6593	0.760774	108.5294	64.12988	Increase
United Arab Emirates	2021	157.1745	0.849318	108.3425	48.83202	Increase
United Kingdom	2021	140.6417	0.859	108.3665	32.27517	Increase
United Republic of Tanzania	2021	167.6033	0.446568	108.3505	59.25287	Increase
United States Virgin Islands	2021	132.8581	0.821831	108.2349	24.62316	Increase
United States of America	2021	203.3248	0.862448	108.3797	94.94509	Increase
Uruguay	2021	198.6337	0.719283	108.3927	90.241	Increase
Uzbekistan	2021	165.1214	0.662622	108.2226	56.89882	Increase
Vanuatu	2021	125.9564	0.473101	108.2721	17.68429	Decrease
Venezuela (Bolivarian Republic of)	2021	142.0459	0.596513	108.3388	33.70716	Increase
Viet Nam	2021	137.3283	0.627934	108.2912	29.03719	Increase
Yemen	2021	134.8254	0.450376	108.25	26.57537	Increase
Zambia	2021	166.4445	0.505949	108.3239	58.12052	Increase
Zimbabwe	2021	162.9864	0.473819	108.3671	54.61928	Decrease
